# Polyhydroxyalkanoate degradation in soils: mechanisms, controls, biological responses, and knowledge gaps

**DOI:** 10.3389/fmicb.2026.1832091

**Published:** 2026-07-02

**Authors:** Tanvi Govil, Priya Saxena, Kritika Thakur, Yash Sharma, Matthew Allen, Jasmeet Kaur, Dipayan Samanta, Rajesh K. Sani, David R. Salem, Ajay Sharda, Vaishali Sharda

**Affiliations:** 1 Karen M. Swindler Department of Chemical and Biological Engineering, South Dakota School of Mines and Technology, Rapid City, SD, United States; 2 Department of Chemistry, Biology, and Health Sciences, South Dakota School of Mines and Technology, Rapid City, SD, United States; 3 Biological and Agricultural Engineering, Kansas State University, Manhattan, KS, United States

**Keywords:** agroecosystem functioning, biodegradation, microbial depolymerization, polyhydroxyalkanoates, soil biogeochemistry

## Abstract

Polyhydroxyalkanoates (PHAs) are increasingly proposed for agricultural applications due to their biobased origin and microbial degradability. They are often assumed to serve as environmentally compatible substitutes for conventional plastics. However, PHA behavior in soil is controlled by the interactions between polymer composition, structure, environmental transport conditions, and microbial activity. PHA degradation proceeds primarily via depolymerase-driven surface-controlled erosion, in which initial mass loss is governed by surface wetting, crystallinity, morphology, exposed surface area, and enzymatic access to ester bonds, which determine the initial rate of breakdown. As degradation progresses, surface disruption allows water, oxygen, and microorganisms to penetrate deeper into the polymer, coupling material breakdown with local microbial activity and nutrient demand. This coupling is central to understanding soil and plant responses. At field-relevant exposure levels, PHA degradation has generally been reported to cause limited or neutral effects on crop performance and bulk soil properties. However, when PHA inputs are high or spatially concentrated, rapid microbial use of PHA-derived carbon can increase demand for nitrogen, phosphorus, and sulfur in localized soil zones. Under these conditions, nutrients may become temporarily immobilized in microbial biomass, reducing plant access and creating short-term plant–microbe competition. Thus, reported growth inhibition is better understood as an indirect soil-mediated response rather than evidence of direct polymer toxicity. This review critically examines laboratories, mesocosms, and field studies to identify the abiotic and biotic factors that control PHA degradation in soils. It evaluates how polymer structure, formulation, processing history, soil environment, and microbial community interact to produce variability in degradation outcomes. The review further identifies the conditions under which PHA degradation may influence soil nutrient dynamics and plant performance. These insights provide a framework for more realistic assessment and responsible design of PHA materials for agricultural systems.

## 1 Introduction

Synthetic plastics are versatile and competent for various industrial, consumer, and agricultural applications (Briassoulis et al., 2020). In agriculture, plastic use continues to rise because these materials, used in many forms such as mulch films, pots, containers, and trays, can help improve crop yields by conserving soil water, increasing soil temperature, maintaining a conductive microclimate, and suppressing weed growth, among other benefits (Briassoulis, 2023). Similarly, the greenhouse and tunnel covers can extend the growing seasons, silage wraps and nettings can help preserve forage quality, and drip irrigation tubing and irrigation pipes can help deliver water efficiently to the roots (Briassoulis, 2023). These functions have driven massive global adoption of plastics in agriculture. In the United States alone, agriculture consumes roughly 1.56 million tons of plastic products, roughly 2.7% of total plastic use nationally, across mulch films, containers, silage and bale wraps, and other Agri plastics (Malarkey and Babbitt, 2025). For global estimates, plastic mulch films usage alone ranges from approximately 2.1–4.0 million tons annually, with growing demand driven by intensification, climate pressure, and the need for higher crop productivity (Khalid et al., 2023). Yet the recovery and recycling of agri-plastic waste remain challenging and extremely low.

Studies consistently report that agricultural plastics return heavily soiled (30–80%) with soil, mud, fertilizers, pesticides, moisture, and crop residues, making both cleaning and transportation to recycling facilities economically inefficient relative to their recycling value (Sarpong et al., 2024; Savenkova et al., 2002). Thin films, such as mulch, silage wrap, and greenhouse covers, also degrade, tear, and fragment during use. As a result, one-third or more agricultural plastics are never recovered from the field or never enter a recycling stream, and are often burned, buried, stockpiled, or left to fragment in the fields themselves (Madrid et al., 2022). Apart from that, removing non-biodegradable synthetic plastic mulch film from soil usually needs to be performed manually by labor. This considerably influences an increase in operating costs ($70–100 per acre; Othman et al., 2022; Zhou et al., 2023). Field-level studies confirm that following mulch use and attempted removal, substantial quantities of macro-sized visible plastic residues (> 1 cm; 89–206 fragments per hectare) and microplastic fragments (< 2 mm; up to 13 mg kg^−1^) can remain behind in the soil (Steinmetz and Schröder, 2022; Long et al., 2023). Plastic mulches, specifically in the case of non-biodegradable polyolefins such as polyethylene-based (PE), ethylene-vinyl acetate (EVA), and polyvinyl chloride (PVC), introduce various additives, such as plasticizing agents, which may pollute the soil (Bandopadhyay et al., 2020b). Over time, plastic film residues in the soil accumulate to a level that begins to affect seed germination and crop growth, putting the safety of the produced food at risk (Wang et al., 2022). Together, these reasons have accelerated an encouraging policy, industry, and end-users’ interest in adopting soil-biodegradable mulch films (BDMs), which promise agronomic benefits and are projected to biodegrade under field conditions without long-term plastic pollution (Madrid et al., 2022).

With bioplastics being required to biodegrade naturally in a controlled way, ideally, polymeric materials comprising BDMs should be part of biomass’s natural life cycle; neither their production nor degradation should have any negative ecological impact. Their biodegradation in the environment should result in harmless natural products. The lower energy of activation for hydrolysis of ester linkages by microbial hydrolases such as esterases (lipase and cutinase) is essential to the biodegradability of BDMs compared to fossil-derived plastics (Bandopadhyay et al., 2020a). Most commercially accepted biopolymers for agricultural use today are formulated from compostable polylactic acid (PLA), starch, polybutylene adipate terephthalate (PBAT), and their blends (e.g., PLA/PBAT/starch; Bandopadhyay et al., 2020a, 2020b). However, several studies show that these materials degrade poorly or inconsistently in agricultural soils, which undermines their presumed environmental benefit. For example, hydrolysis of PLA is accelerated at temperatures typically above 50–60 °C, but at ambient soil temperatures (20–30 °C in most temperate climates), PLA degradation is extremely slow (Van de Perre et al., 2024). A study reports that in a soil microcosm study at ~30 °C, only 10% of PLA was mineralized in 150 days, and even with microbial bio-augmentation and stimulation, mineralization reached only ~22–24% over that time (Satti et al., 2018). This indicates that over a typical growing season or even several seasons, most PLA can remain largely intact in soil. A field burial study of a commercial starch/PBAT mulch film over 478 days showed that the film’s surface area, owing to its aliphatic segments, decreased by 57% (pristine film) and 66% (UV-aged film) (Convertino et al., 2024), but its aromatic terephthalate moieties (not easily cleaved by typical soil enzymes) tended to accumulate. Comparatively, in starch-based polymer blends, soil microbes rapidly consume the starch portion, which may trigger physical breakdown (fragmentation), but the remaining synthetic matrix often exhibits minimal mineralization and can remain in the soil for months, far longer than their certification labels imply, where temperatures are low, moisture fluctuates, and the key hydrolytic enzymes are scarce (Francioni et al., 2022).

Against the backdrop of PLA-, PBAT-, and starch-based films that fragment rather than truly mineralize, polyhydroxyalkanoates (PHAs), including polyhydroxybutyrate (PHB) and its copolymer polyhydroxyvalerate (PHV), are often positioned as the biologically natural alternative (Fukala and Kučera, 2024). PHA biopolymers are considered a perfectly biocompatible polyester for agricultural usage, a true microbial polyester whose depolymerization pathways are already present in soil ecosystems. PHA disintegrates without causing environmental damage since its biodegradation products are water and CO_2_ (Gasparyan et al., 2023). Numerous soil and rhizosphere microorganisms are known to secrete extracellular PHA depolymerases capable of cleaving PHB, poly(3-hydroxybutyrate-co-3-hydroxyvalerate) (PHBV), and other copolymers under ambient conditions. Laboratory studies consistently report measurable weight loss, CO_2_ evolution, and microbial colonization on PHA-based films (Chang et al., 2025; Gibbs et al., 2025). However, its field behavior shows greater variability than theory. While some reports describe substantial degradation of PHA or PHBV films in moist, biologically active soils, other reports describe slow, uneven, or incomplete degradation, driven by differences in soil moisture, polymer crystallinity, carbon limitations, microbial depolymerase abundance, and local agronomic practices. Reports suggest that PHA biodegradation proceeds only after long microbial acclimation phases under real agricultural conditions (Chang et al., 2025). There is clear evidence that the abundance of PHA-degrading microorganisms can differ by 100-to 1,000-fold between soils (Paloyan et al., 2025), and without a field-level understanding of the factors that truly govern PHA biodegradability, it risks repeating the same environmental shortcomings as the very materials it seeks to replace. Furthermore, other studies raise practical concerns that incomplete degradation can produce localized hotspots, altered soil C:N ratios, shifts in fungal and bacterial composition, and temporary immobilization of nitrogen, with potential downstream effects on seedling vigor, root colonization, and soil nutrient availability (Kaur and Chauhan, 2024). Therefore, if PHAs are truly to serve agriculture, the critical questions are no longer whether PHAs are theoretically biodegradable, but whether they can reliably biodegrade in the diverse soil environments where agriculture operates.

This review aims to place existing studies on PHA biodegradation in soils into a realistic environmental context, determining the available evidence implies for PHA behavior once these materials are introduced into soil systems, including agricultural and plant-associated settings. Rather than treating biodegradation as an intrinsic material property, the review examines how degradation outcomes emerge from interactions between polymer structure, soil conditions, and microbial activity. A further objective is to identify unresolved uncertainties that limit confident interpretation of current results and constraints on large-scale deployment. The review is therefore organized around a set of focused questions, including but not limited to the following:

1. How does PHA degradation initiate and progress in soils once materials are introduced into realistic environments, and what evidence supports surface-controlled erosion as the dominant degradation pathway?
2. Which polymer properties and processing choices, such as crystalline, copolymer composition, blending, thickness, and geometry, most strongly determine whether PHA degradation is rapid, slow, or incomplete in soils?
3. How do soil conditions (e.g., moisture, oxygen availability, temperature, and placement within the soil profile) interact with microbial communities to regulate PHA depolymerization and mineralization?
4. To what extent do current laboratory and short-term soil burial studies capture the variability observed under field conditions, and where do methodological limitations restrict extrapolation to real soil systems?
5. What is known about the formation, persistence, and fate of intermediate degradation products (e.g., monomers, oligomers, and crystalline residues), and how well are these stages resolved in existing studies?
6. What soil and plant responses have been reported during PHA degradation, and how consistently can these responses be attributed to biologically mediated processes such as microbial carbon utilization and nutrient competition rather than direct material effects?

## 2 Methodology: systematic review framework using PRISMA

### 2.1 Rationale and scope of the review

**Figure 1:**
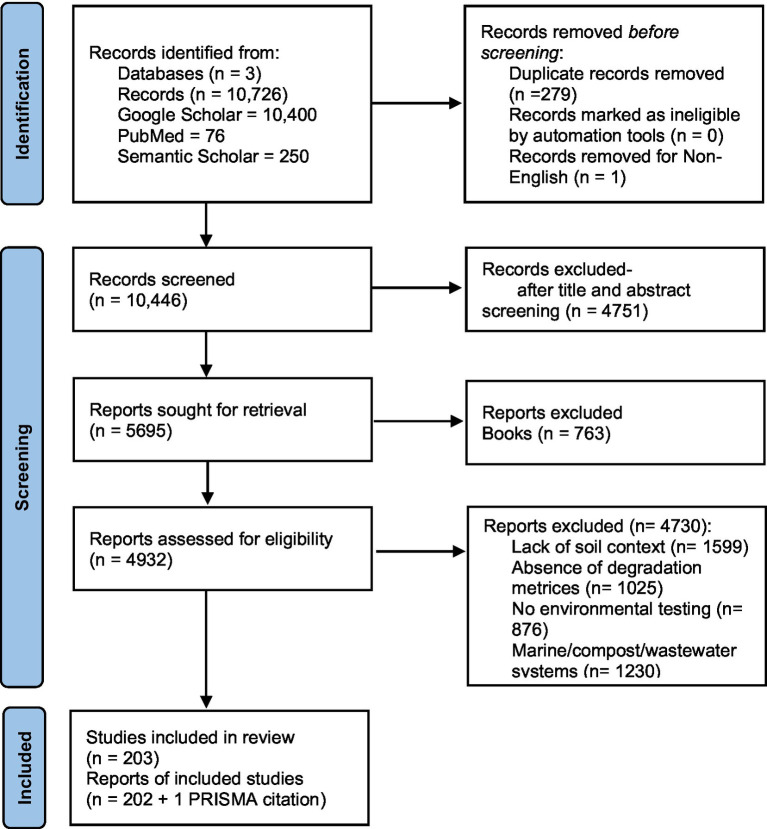
PRISMA flowchart for literature (1) identification, (2) screening, and (3) inclusion.

This systematic review was conducted in accordance with the PRISMA 2020 (Preferred Reporting Items for Systematic Reviews and Meta-Analyses) guidelines to ensure transparency, reproducibility, and methodological rigor in synthesizing literature related to polyhydroxyalkanoate degradation in soil environments (Tugwell and Tovey, 2021). The objective of this review was to critically evaluate experimental evidence describing soil-mediated degradation of polyhydroxybutyrate, polyhydroxyalkanoates, and polyhydroxybutyrate-co-valerate, with emphasis on measurable degradation endpoints, including mass loss, carbon dioxide evolution, mineralization rates, and molecular weight reduction. The review specifically focused on soil-based degradation systems, including laboratory soil burial experiments and field soil studies. Compost-only, aquatic-only, marine-only, and wastewater-focused degradation studies were excluded unless directly linked to soil burial transitions. The temporal scope of the review covered publications from January 2000 to January 2026 to capture contemporary advancements in biodegradable polymer degradation under realistic soil conditions (Figure 1).

### 2.2 Search strategy and information sources

A structured Boolean search strategy was implemented to minimize the retrieval of irrelevant literature related to polymer synthesis, biomedical applications, or microbial PHA production systems. The following query was applied consistently across databases: (polyhydroxybutyrate OR polyhydroxyalkanoate OR PHBV) AND (soil burial OR soil degradation OR soil biodegradation) AND (mass loss OR CO₂ evolution OR mineralization OR molecular weight). Searches were conducted across three interdisciplinary scholarly platforms: Google Scholar, PubMed, and Semantic Scholar. The initial search identified a total of 10,726 records, comprising 10,400 records from Google Scholar, 76 records from PubMed, and 250 records from Semantic Scholar. Google Scholar provided the broadest interdisciplinary coverage, while PubMed contributed microbiologically focused degradation studies. Semantic Scholar facilitated cross-domain retrieval of polymer, soil science, and environmental engineering publications.

### 2.3 Study selection and screening process

All 10,726 records were exported into reference management software for consolidation and duplicate removal. After removal of 279 duplicate records across databases, and non-English articles (*n* = 1), 10,446 unique records remained and were advanced to title and abstract screening. During the screening stage, 4,751 records were excluded based on predefined screening criteria when their title and abstracts clearly indicated that they were outside the scope of the review. These exclusions included records focused primarily on biomedical applications, polymer synthesis without environmental testing, microbial PHA production, non-soil degradation environments, or articles that did not report degradation-relevant outcomes. Records for which relevance could not be determined from the title and abstract alone were retained for full-text assessment.

Following title and abstract screening, 5,695 records were retained for further evaluation. At this stage, 763 records identified as books or book-like records were removed, leaving 4,932 reports for full-text eligibility assessment. Full-text assessment was then conducted using predefined eligibility criteria to determine whether each report met the inclusion criteria for soil-based PHA degradation. At this stage, 4,730 reports were excluded with documented reasons, including lack of soil context (*n* = 1,599), absence of measurable degradation metrics (*n* = 1,025), focus on polymer synthesis without environmental testing (*n* = 876), or exclusive investigation of marine, compost, or wastewater systems without relevance to soil degradation (*n* = 1,230).

Following the eligibility assessment, 202 primary research studies met all inclusion criteria and were included in the final evidence synthesis. One additional reference was retained for PRISMA methodology, resulting in 203 total references in the revised manuscript. The revised PRISMA flow diagram now separates identification, duplicate removal, title/abstract screening, full-text eligibility assessment, exclusion reasons, and final inclusion.

### 2.4 Eligibility criteria, data extraction, and thematic synthesis

Studies were included if they:

1. Investigated PHB, PHA, or PHBV materials
2. Conducted degradation experiments in soil matrices
3. Reported at least one quantifiable degradation metric (mass loss, CO_2_ evolution, mineralization rate, or molecular weight reduction)
4. Provided sufficient experimental detail regarding soil physicochemical conditions

Studies were excluded if they were review articles without new data, non-English publications, conference abstracts, or lacked measurable degradation outcomes. This strict filtering ensured that the final dataset of 202 included studies represented experimentally validated soil-based PHA degradation research.

### 2.5 Quality assessment of included studies

Methodological robustness of the included studies was assessed qualitatively during full-text evaluation. Studies were examined for clarity of experimental design, presence of replication, reporting of statistical analysis, description of soil physicochemical parameters, and distinction between abiotic and biotic degradation processes. Particular attention was given to studies that clearly quantified degradation metrics such as mass loss, CO_2_ evolution, mineralization rates, or molecular weight reduction under well-defined soil conditions. Greater interpretive weight was assigned to studies integrating polymer physicochemical characterization with microbial or soil biochemical analyses, as these provided stronger mechanistic insight into soil-mediated degradation processes. Field-based investigations were also considered especially valuable due to their ecological relevance compared to controlled laboratory soil microcosms.

### 2.6 Limitations of the review process

Despite the use of a restrictive and targeted search strategy, certain limitations should be acknowledged. Broad indexing platforms required extensive screening to remove interdisciplinary records not directly related to soil-based degradation, while biomedical-focused databases provided comparatively narrower environmental coverage. Variability in experimental design, soil characterization, incubation conditions, and degradation measurement methods limited direct cross-study comparability and precluded quantitative meta-analysis. Additionally, the predominance of laboratory soil microcosm studies relative to long-term field investigations may constrain ecological generalizability. Restriction to English-language publications may also have excluded region-specific field studies. These limitations highlight the need for standardized testing protocols and harmonized reporting frameworks in soil biodegradation research.

## 3 Lamellar structure and chain organization of PHA: formation, architecture, and consequences

As a bio-based thermoplastic synthesized by microbes under carbon-limiting conditions, PHAs have gained popularity over the years for being entirely biodegradable, produced from renewable resources, and biocompatible alternatives to conventional plastics. PHAs are polyesters typically composed of on the order of 10^3^–10^4^ monomer units and accumulate intracellularly as discrete inclusions, generally 0.2–0.5 μm in diameter when carbon is abundant but key nutrients (N, P, O, and S) are limiting. Under these conditions, PHA accumulation enables cells to maintain redox balance, survive nutrient scarcity, and buffer against osmotic or oxidative stress (Govil et al., 2020). At the basic level, PHAs are classified into short-chain-length (scl-PHA) and medium-chain-length (mcl-PHA) materials, based on the number of carbon atoms in the monomer’s side chain. Scl-PHAs, such as poly(3-hydroxybutyrate) (PHB), poly(3-hydroxyvalerate) (PHV), and their copolymers (e.g., PHBV, PHB-co-PHV, or copolymers with 4-hydroxybutyrate (4HB)), are built from monomers with short side chains containing 3–5 carbon atoms. In contrast, mcl-PHAs consist of monomers with longer side chains, typically containing 6–14 (or more) carbon atoms, for instance, 3-hydroxyhexanoate (3-HHx), 3-hydroxyoctanoate (3-HO), up to 3-hydroxydodecanoate (3-HDD) (Koller et al., 2025; Reddy et al., 2022). Through microbial biosynthesis and metabolic engineering, researchers have produced a wide array of other PHAs, including scl-homopolymers (PHB, PHV, and P4HB), scl-copolymers (PHBV, PHB-co-4HB, and PHV-co-3HB), mcl-homopolymers (P3HHx, P3HO, and P3HDD), and mixed scl/mcl copolymers or block polymers (e.g., PHB-co-P3HHx and PHB-P3HO block copolymers). This structural diversity influences not only the thermomechanical properties of the PHA material but also its biodegradation behavior (Koller et al., 2025).

Bacteria accumulate PHAs under carbon excess and nutrient limitation to maintain redox balance and enhance stress tolerance (Govil et al., 2020). This biological origin is important because it dictates how the polymer chains are built and how they pack together (Maestro and Sanz, 2017; Muneer et al., 2020; Bresan et al., 2016). During biosynthesis, PHA synthase (PhaC) enzymes add monomers one at a time in a highly controlled, stereospecific manner, producing isotactic polymer chains in which all side groups are arranged in a uniform orientation along the backbone (Mondol et al., 2025). For PHAs composed of monomers with short side chains, this stereoregular architecture promotes dense chain packing and high crystallinity, yielding materials with relatively uniform molecular organization (Koller et al., 2025). A clear example is poly(3-hydroxybutyrate) (P(3HB)), which is built from 3-HB monomers containing only a short methyl side group. Because these side groups are small and identical, PHB chains can align closely and pack in a highly regular manner, similar to neatly stacked sheets. This efficient packing allows 3-HB chains to crystallize into dense lamellar structures that form a rigid internal scaffold within the PHA granule. As a result, P(3HB) granules contain large, continuous crystalline regions that are physically difficult for water and depolymerase enzymes to penetrate.

**Figure 2:**
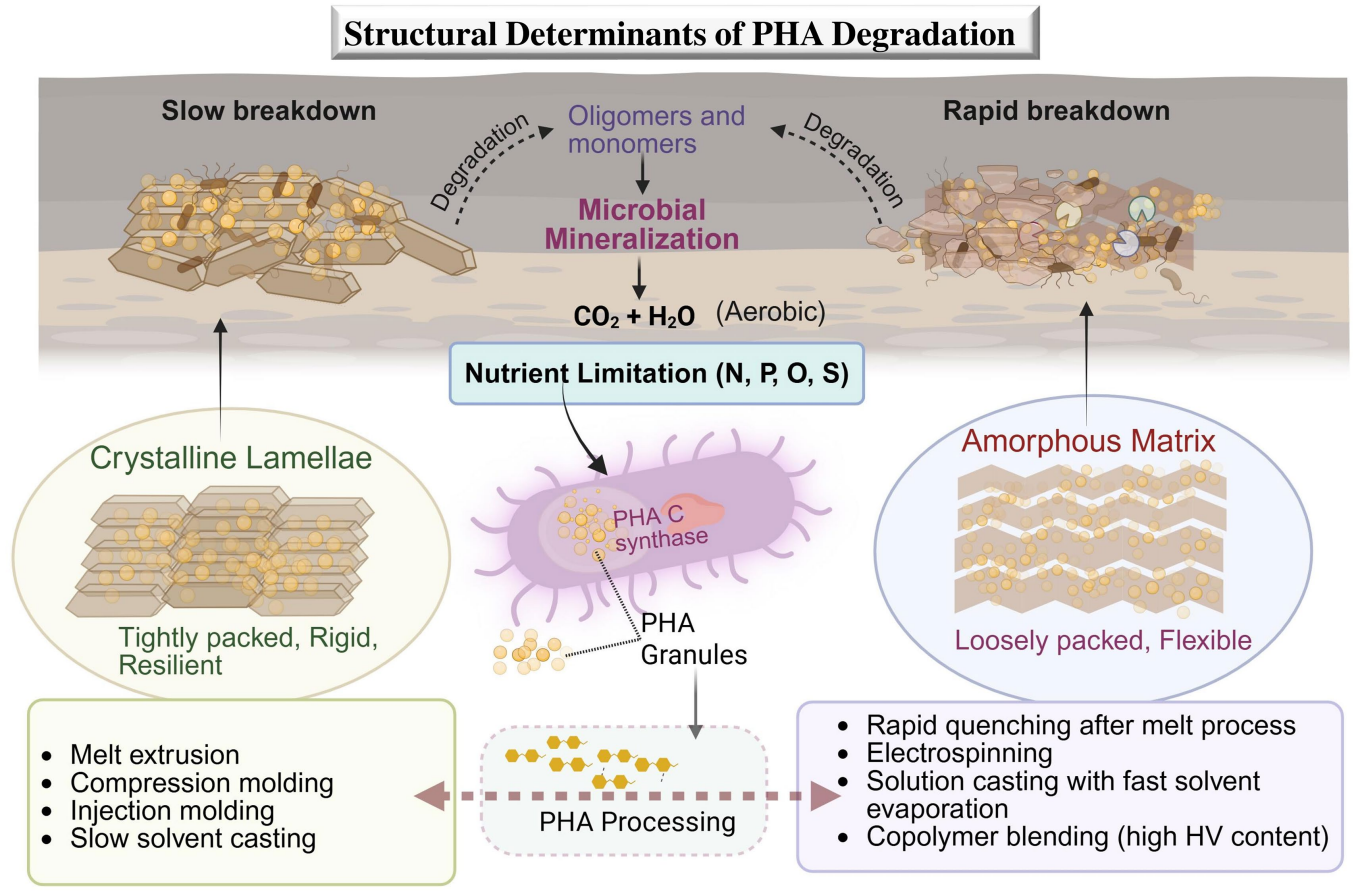
Structural determinants of polyhydroxyalkanoates (PHA) degradation. This diagram illustrates how quickly PHA breaks down, which depends largely on its internal structure. PHA crystallinity slows degradation, whereas amorphous morphologies accelerate microbial breakdown to CO₂ and H₂O under aerobic conditions.

However, not all chain segments can pack into ordered crystalline lamellae. When longer or more flexible side chains are introduced into the PHA backbone (e.g., HV units in PHBV), they disrupt regular alignment and are therefore excluded from lamellar packing, forming the surrounding amorphous matrix of the PHA granule (Volova et al., 2023). As a result, crystalline lamellae become smaller and less continuous, producing materials with lower crystallinity and more amorphous regions. While the crystalline lamellae provide stability and rigidity, the amorphous regions are more mobile and physically accessible, allowing increased water penetration and enzyme access (Figure 2). This internal organization is further regulated at the granule surface by small amphiphilic proteins, including phasins (PhaPs), PHA depolymerase (PhaZ), and other granule-associated regulatory proteins such as PhaR. Phasins coat the hydrophobic PHA core, control granule number and size, and limit excessive crystallite growth or granule coalescence. These features are specific to native intracellular PHA granules and govern biological storage and turnover within microbial cells, rather than the degradation behavior of processed PHA materials in soil (Zhao et al., 2016; Mezzina and Pettinari, 2016; Volova et al., 2017).

From a biodegradation standpoint, this lamellar organization creates a two-speed degradation pathway. In soil, extracellular PHA depolymerases and other hydrolases first attack the polymer primarily in amorphous regions, where chains are less tightly packed, more hydrated, and accessible. Biodegradation, as it proceeds, produces surface roughening and generates oligomers and monomers that microbes can assimilate (Bandopadhyay et al., 2020a; Fernandes et al., 2020). The crystalline lamellae, however, are much more difficult to attack, as they present densely packed helices with minimum free volume (Julinová et al., 2023). Enzymes cannot easily penetrate these dense, ordered regions, and hydrolysis of ester bonds is slower when chains are locked in a crystalline lattice (Koller et al., 2025; Volova et al., 2017; Sridewi et al., 2006). As amorphous material is consumed, the relative proportion of crystalline domains increases, rendering the remaining material progressively more resistant to further degradation (Kim et al., 2023). Consistent with this preferential consumption, many soil burial studies report rapid loss of amorphous phases, while crystalline PHB-rich domains persist as residual fragments in soil for extended periods, sometimes for months particularly under suboptimal moisture conditions or when depolymerase abundance is low Therefore, because of this native microstructure, any parameter that shifts the crystalline amorphous balance, at the molecular level (e.g., PHB: PHV ratio and chain regularity), the processing level (extrusion temperature, cooling rate, annealing, and solvent casting), or formulation level (plasticizers, fillers, copolymers, and surface treatments), can effectively play a role in determining how fast or how completely PHA will biodegrade. A consistent pattern across the literature is that higher crystallinity correlates consistently with slow mass loss and persistence of crystalline micro fragments, as demonstrated in controlled humidity studies (Kim et al., 2023), compost and soil burial experiments (Prudnikova et al., 2024), and structure–function analyses of PHB and PHBV films (Sridewi et al., 2006; Tarazona and Lendlein, 2020; Rivera-Briso and Serrano-Aroca, 2018). Conversely, lowering crystallinity through increased 3-hydroxyvalerate or other hydroxy acids content (Rivera-Briso and Serrano-Aroca, 2018; Koike et al., 2024; Zhuikov et al., 2020; Wei and McDonald, 2016; Abbasi et al., 2022; Alfano et al., 2024; Feijoo et al., 2022), rapid quenching (Mondol et al., 2025), blends or composites (Eesaee et al., 2022; Han et al., 2023), and introducing hydrophilic fillers (Chaber et al., 2024) can reliably increase water uptake, accelerate enzyme access, and enhance biodegradation. Recent integrative reviews similarly highlight crystallinity and chain packing as central predictors of environmental fate (Dulac et al., 2025).

However, crystalline alone does not determine PHA biodegradation in soil or in an agricultural environment. Once PHA enters an agricultural field, its breakdown becomes constrained by multiple external factors, e.g., soil moisture, wet–dry cycling, oxygen availability, temperature fluctuations, soil aggregation and porosity, organic matter content, pH, and most critically, the presence and activity of PHA-degrading microbial guilds (Paloyan et al., 2025; Koller et al., 2025; Fernandes et al., 2020; Lin et al., 2025). These environmental controls interact with intrinsic material properties such as molecular weight, copolymer composition, surface chemistry, film thickness, and processing-derived morphology (Dulac et al., 2025; Kim et al., 2023). As a result, field outcomes diverge even for chemically identical PHAs, as some soils support rapid depolymerization, while others show negligible degradation over months (Koller et al., 2025). This variability underscores that PHA degradation is a multi-parameter systems phenomenon, and not a single property material behavior.

## 4 Factors impacting PHA biodegradation in agricultural soils

### 4.1 Material driven controls

#### 4.1.1 Dependence on PHA polymer composition

PHA biodegradation in soil is fundamentally a surface-mediated erosion process that proceeds from the outer surface inwards. It is initiated by microbial attachment, biofilm formation, and secretion of extracellular PHA depolymerases onto the exposed polymer interface. Consequently, accessibility of the PHA surface, rather than bulk polymer chemistry, acts as the dominant rate-limiting factor governing degradation in soil environments. In this context, the copolymer composition of PHA and the side chain structure of comonomer influence the extent to which ester bonds are accessible to water penetration, enzyme binding, and microbial attack, thereby modulating interactions between PHA and the surrounding soil environment (Paloyan et al., 2025). As discussed in Section 3, copolymer composition is a key determinant of PHA degradability through its influence on polymer structure. In this section, we focus on how variations in polymer composition translate into differences in degradation behavior under soil and microbial conditions.

As outlined in the previous section, increasing side-chain length influences degradation behavior across PHA types. From this structural starting point, one can make mechanistic predictions for agricultural soils, and some of the published soil and microcosm experiments support this picture. For instance, Sevakumaran et al. (2019), reported that among P(3HB) and its copolymers with 4HB at different percentages, P(3HB-*co*-87% mol 4HB) had the highest percentage of degradation at 98.9 ± 1.8% followed by P(3HB-*co*-47% mol 4HB) at 84.2 ± 2.9%, P(3HB-*co*-14% mol 4HB) at 44.8 ± 1.8%, and P(3HB) at 56.7 ± 2.3%, respectively, at the end of the 5 weeks of burial in the simulated soil during laboratory test. Similar results had also been reported in yet another study, where in an agro-transformed field zone, biodegradation of PHA polymers was reported in the order following P(3HB/4HB) > P(3HB/3HHx) > P(3HB/3 HV) > P(3HB). The authors explicitly mentioned P(3HB/4HB), which had the lowest degree of crystallinity (50%), and showed the highest degradation rate (1.63 mg/day); while the most highly crystalline (78%) P(3HB) was degraded with the slowest rate (0.61 mg/day) (Volova et al., 2017). Similarly, studies also confirm that mcl-PHAs containing 3HHx units, such as P(3HB/3HHx), degrade substantially faster, often by up to a factor of two, than P3HB homopolymers or P(3HB/3 HV) copolymers under comparable soil and sediment conditions (Volova et al., 2017; Sridewi et al., 2006).

However, this trend is not universal. Li et al. demonstrated in controlled enzymatic assays that while P(3HB-co-19%-3 HV) degraded faster than PHB, copolymers with even longer side chain monomers, such as P(3HB-co-3HHx) and P(3HB-co-3HO), degraded more slowly despite exhibiting lower crystallinity (Li et al., 2007). This suggests that steric hindrance from extended side chains can impede depolymerase binding and override gains from increased amorphousness. Supporting this non-linear relationship, Arcos-Hernandez et al., in a soil burial study using PHBV with HV contents ranging from 12 mol% to 72 mol%, showed that PHBV films achieved maximum soil mineralization at intermediate HV contents (47%), whereas very high levels of HV(72%) led to reduced degradation (Arcos-Hernandez et al., 2012). Therefore, at high comonomer contents, non-linear degradation behavior is often observed under soil conditions.

In most agricultural soils, microbes are far more familiar with scl PHAs such as PHB, which resemble common intracellular storage polymers. In contrast, microorganisms capable of efficiently degrading mcl-PHAs or highly substituted P(3HB/3 HV) copolymers appear to be less abundant. Consequently, observed differences in PHA degradation across studies often reflect variation in the depolymerase repertoire of the resident microbial community rather than intrinsic resistance of the polymer itself. Because PHA depolymerases exhibit strong substrate specificity, only polymers that match locally available enzyme sites are rapidly cleaved (Batista et al., 2010). Complex microbial populations secrete diverse pools of depolymerases. In heterogeneous soil communities, multiple depolymerases may coexist, but their relative abundance and activity may ultimately govern degradation rates and pathways. This finding underscores the importance of the indigenous microbial community, soil bacteria, and fungi that adapt to prevalent carbon sources. If a soil has PHA degraders primed for a particular PHA type, that polymer will vanish astonishingly fast. In practical terms, this hints that agricultural soils with a history of PHA or similar biomass (e.g., plant waxes and polyesters) exposure may show accelerated degradation of PHA-based inputs.

#### 4.1.2 Formulation driven controls

The blending of PHAs with chemical additives or naturally decomposable materials, such as cellulosic fibers or agro-residues, is a common approach used to adjust the mechanical properties of PHA (Fernandes et al., 2020). However, from an environmental standpoint, it is equally important to understand how such formulation strategies influence the susceptibility of PHA to microbial degradation in soil. When the studies summarized in Table 1 are evaluated collectively, a consistent pattern becomes evident that the reported mass loss or mineralization of PHA-based materials spans from <5% to nearly complete degradation within similar timeframes, largely as a function of formulation architecture. Certain biodegradable fillers or porous natural fibers can accelerate apparent biodegradation by introducing structural heterogeneity, increasing surface roughness, enhancing water uptake, and creating discontinuities that facilitate microbial colonization and enzyme penetration (Batista et al., 2010; Wu, 2013; Wu, 2014; Zaidi et al., 2019; Chan et al., 2019; Lammi et al., 2019; Thomas et al., 2020; Joyyi et al., 2017; Madbouly et al., 2014). Similarly, in soil burial systems, electrospun PHB nanofibers exhibit rapid and near-complete weight loss within weeks, whereas solvent-cast PHB films degrade substantially more slowly under comparable conditions, reflecting the transition from surface-limited erosion to bulk-accessible degradation in porous structures (Altaee et al., 2016).

**Table 1:** Formulation-driven controls on soil biodegradation of PHA-based polymers, blends, and composites.

Study	Test setting and verification design	Climate and soil conditions	Matrix and filler/additive/copolymer/plasticizers	Thickness	Mineralization (% weight loss)	Notes on mechanism
Kim et al. (2023)	Laboratory soil burial between mesh nets. Verification by visual observation, FTIR, DSC, tensile testing, SEC, and DFT support.	Potting mix soil at pH 6.6–6.7 and 25 °C. Compared saturated soil at 100% RH with soil held at 40% RH.	PHB and PHBV with 8 wt% HV.	PHB 15 ± 5 μm. PHBV 10 ± 2 μm.	In saturated soil at 100% RH, both PHB and PHBV were completely degraded within 2 weeks. In soil at 40% RH, only a limited change occurred over 6 weeks.	Water availability strongly controlled abiotic hydrolysis and subsequent microbial metabolism. Once hydrolysis reduced the molar mass, microorganisms rapidly consumed the fragments.
Jeszeová et al. (2018)	Indoor; agricultural topsoil burial: 350 days	pH 7.5; moisture ~85%	PLA/PHB films (unfilled, carbon-black filled, weathered black)	Melt extruded films: 0.1 mm thick	White PLA/PHB: 57%; weathered black: 55%; black: 42%.	Carbon black reduces surface wettability and microbial accessibility; photoweathering introduces surface defects that partially restore access.
Fernandes et al. (2024)	Indoor; agricultural topsoil burial: 180 days	pH 7.5; moisture ~90%	PHB; PHB/PBAT: 55/45 wt%	Melt extruded mulch films: 35 um thick	PHB (~100%) > cellulose (~75%) > PHB/PBAT (~47%) > PBAT (< 30%).	PBAT dilutes PHB and slows mineralization via hydrolysis-dominated pathways; PHB-rich domains initiate microbial attack.
Zaidi et al. (2019)	Outdoor natural soil burial in a trench for 112 days. Samples were buried 20 cm deep in triplicate. Verification by weight loss, optical microscopy, and SEM	Sandy Botany Sand in Sydney is under natural rainfall. Outdoor exposure began on 28 April 2017.	PHBV with 3 mol% HV. Unidirectional flax at 30 vol. Toughened with PBAT or ENR 50 at 30 vol%.	Composite laminate thickness approximately 1.5 mm.	After 112 days, neat PHBV lost 0.5% mass, PHBV flax lost 6%, PHBV PBAT flax lost 9%, and PHBV ENR flax lost 17%.	Flax and toughener interfaces created paths for microbial ingress. Reduced crystallinity increased accessibility. ENR likely added a further biodegradable phase that soil microbes could attack.
Altaee et al. (2016)	Outdoor garden soil burial for 6 weeks. Samples were cut into 1 × 1 cm pieces, placed in mesh bags, and buried at a depth of 10 cm.	Fertile garden soil at 7.30 pH, 30 °C, 80% humidity.	PHB cast films and electrospun PHB nanofibers. PHB TiO₂ composites. UV-treated and untreated formulations.	Cast film not reported. Nanofiber diameter approximately 500 nm for PHB and 550 nm for PHB TiO₂.	Electrospun PHB nanofibers reached 100% weight loss within 3 weeks. After 6 weeks, the cast PHB lost approximately 62%, the UV-treated PHB approximately 68%, the PHB TiO₂ approximately 51%, and the UV-treated PHB TiO₂ about 56%.	High surface area drove rapid degradation of nanofibers. UV pretreatment increased susceptibility. TiO₂ slowed degradation relative to neat PHB. Degradation was attributed to microbial depolymerase activity and surface erosion.
Rani-Borges et al. (2016)	Indoor; garden soil; 8 cm deep burial; 90–120 days	Specific conditions not reported	PHBV/PP-co-PE (80/20 wt%) and PHBV/PP-co-PE/add (80/19/1 wt%) with pro-oxidant additive	Melt extruded films: 80–100 μm thick	PHBV (90–100%) > PHBV/PP-co-PE/Add (~88%) > PHBV/PP-co-PE (~77–93% > PP-co-PE (<0.3%)	PHBV acts as the degradable phase; PP-co-PE remains inert; pro-oxidants increase surface defects, restoring microbial access to PHBV domains.
Kuntanoo et al. (2013)	Outdoor; Garden Soil; 20 cm deep burial; 45 days	pH 6.7	P(3HB) with natural rubber (NR)	Melt extruded films: 1.6 mm thick	PHBV (~20%) > PHBV/NR (~20%) > NR (~10%)	Natural rubber phase persists; PHB degradation is limited by phase dilution and restricted accessibility.
Muniyasamy et al. (2016)	ASTM D5988 soil respirometry in biometer flasks over 200 days. Blank and cellulose control. Triplicate testing. Verification by CO₂ trapping and back titration	Agricultural field soil from South Africa. pH 7.2, 23–25 °C, total organic carbon 3.4%, C: N ratio 28:3	PHBV with 12% HV and 10% citric acid plasticizer. PLA PHBV blend at 70 to 30. PLA control.	Solution cast films about 10–11 μm.	After 200 days in soil, PHBV reached 35% mineralization, and PLA PHBV reached 32%. PLA reached 4%.	Biodegradation proceeds by hydrolytic and enzyme-catalyzed chain scission. PHBV promoted some breakdown of PLA-rich blends, but soil conditions remained unfavorable for strong PLA mineralization.
Batista et al. (2010)	Indoor; mixed fertile soil: 150 days	pH 6.5–7.5; 20–30% moisture	PHBV + peach-palm particles (0–25 wt%)	Melt extruded films; 1.6 mm thick	PHBV loss ↑ from ~20% (90/10) to ~35–40% (80/20)	Lignocellulosic fillers generate interconnected microvoids (≈2.5–10 μm), increasing water uptake and microbial entry points.
Mousavioun et al. (2012)	Indoor; garden soil: 20 cm deep burial, 360 days	12–27 °C; 20% moisture; pH 6.7	PHB/lignin blends (0–10 wt%)	Melt extruded films; 100 um thick	PHB (45 wt%) > PHB + 10 wt% lignin (12 wt%)	Lignin forms dense hydrogen-bonded networks with PHB, limiting water uptake and microbial colonization.
Wu (2013)	Indoor; alluvial farm top soil; 12–15 cm deep burial: 360 days	~35% moisture	PHA-g-MA/crosslinked tea plant fiber (t-TPF) composites (20 wt%)	Melt extruded films; 1.0 mm thick	PHA-g-MA/t-TPF (> 30%) > PHA t–t TPF ~ 30% > PHB > PHA-g-MA	MA grafting improves fiber dispersion; ester linkages enhance interfacial connectivity and microbial accessibility when not over-crosslinked.
Wu (2014)	Indoor; alluvial farm top soil: 12–15 cm deep burial, 360 days	~35% moisture	PHA/rice-husk and acrylic-acid-grafted PHA/RH	Melt extruded films; 1 mm thick	PHA/RH (~96%) > PHA-g-AA/RH (90%) > PHA	Rice husk improves interfacial wetting and disrupts PHB packing; acrylic grafting partially seals interfaces despite increased polarity.
Zaidi et al. (2019)	Outdoor; soil burial: 112 days	Specific conditions not reported	PHBV/flax; PHBV/PBAT/flax; PHBV/ENR/flax (30 wt% flax)	Compression-molded films; 1 mm thick	PHBV ~0.5%; flax composites 7–17%	Flax fibers create interfacial cracks and water pathways; fiber biodegradation opens the matrix; ENR recruits rubber-degrading microbes.
Chan et al. (2019)	Outdoor; > 7 cm deep soil burial: 360 days	~19.8°C; moisture ~80%	PHBV + wood flour (0–50 wt%)	Melt extruded films; 1.6 mm thick	PHBV/50 wt% WF (~13%) > PHBV (~2.6%)	Increasing filler content increases surface area, moisture retention, and microbial colonization, linearly enhancing degradation.
Barkoula et al. (2010)	Outdoor; Garden Soil; 25 cm deep burial: 360 days	Specific conditions not reported	Flax-reinforced PHB/HV (8 wt% HV, 20 wt% flax)	Injection molded Pellets	Not quantified	Pellet geometry and thickness impose diffusion limitations; fiber reinforcement promotes only localized surface erosion.
Lammi et al. (2019)	Indoor; bulk soil mixture: 120 days	~28 °C; moisture ~80%	PHBV/Olive Pomace (OP) (85/15 wt%)	Particles 1–2 mm	PHBV/OP (~100%) > PHBV (~91%)	Olive pomace supplies bioactive carbon, retains moisture, and creates interfacial defects that stimulate microbial activity.
Thomas et al. (2020)	Outdoor; Agro-transformed soil: 2 cm deep burial: 35 days	~25 °C; moisture ~50%	P(3HB) with natural fillers (peat, birch wood flour, and clay)	Cold-pressed pellets and granules	(a) Pellets: P(3HB)/peat ~44% > P(3HB)/clay ~36% > P(3HB)/wood flour ~33% > P(3HB) ~ 32%. (b) Granules: P(3HB)/peat ~54% > P(3HB) ~ 32% > P(3HB)/clay ~26 > P(3HB)/wood flour ~23%	Peat enhances degradation via moisture retention and microbial stimulation; clay provides physical water pathways; wood flour shields PHB surfaces.
Gonçalves et al. (2018)	Indoor; Red Clay soil amendment with 0–10 wt.% sugar cane bagasse biochar: 30 days	~25°C; moisture ~50%	P(3HB) with silver nanoparticles (AgNP)	Solvent-Cast Films	At 10% biochar: PHBV (no AgNP): ~25–30% loss > PHBV + AgNP: ~14–17% loss AgNP reduces biochar-driven degradation by ~40–50%	Biochar increases moisture retention and microbial habitat; AgNPs suppress microbial activity, reducing biochar-driven degradation.
Joyyi et al. (2017)	Indoor; Oil palm cultivation soil: ~ 42 days	30 ± 2 °C; and moisture 81 ± 2%	mcl-PHA P(3HB-co-3HHx) with 30 wt% kenaf fibers	Compression molded pellets; 130 × 12 × 3 mm3	PHBHX-KF composite ~ 10–14% > neat PHBHX (~6%)	Fiber inclusion promotes water ingress and interfacial debonding; degradation initiates fiber-matrix defects and progresses inward.
Madbouly et al. (2014)	Outdoor; Horticulture ground; 10 cm soil burial: 168 days	~21 °C; moisture ~50%	PHB with distiller’s dried grains with solubles (DDGS) (90/10 wt%)	Injection-molded plant containers	~6 × higher mass loss for PHB/DDGS vs. neat PHB	DDGS acts as a nutrient-rich, hydrophilic filler, increasing microbial colonization and accelerating PHB breakdown.
David et al. (2019)	Aerobic soil respirometry adapted from ASTM D5988. Blank and cellulose control. Triplicate testing. Curves modeled from cumulative CO₂ evolution	Topsoil at pH 7.5 and C: N ratio 3:9. Incubation at 28 ± 1 °C. Water content adjusted to 80% of water holding capacity	PHBV with 1–3 mol% HV. Filled with 20 wt% pure vine shoots or exhausted vine shoots after polyphenol extraction	Composite tested as milled particles with a median size of about 350 ± 20 μm.	Neat PHBV reached 98% mineralization. PHBV with 20 wt% pure vine shoots reached 102%, and PHBV with 20 wt% exhausted vine shoots also reached 102%. Plateau was reached by about 55 days.	Fillers slightly accelerated PHBV by increasing water diffusion and creating interfacial gaps. Polyphenols in virgin filler suppressed filler biodegradation, while extraction improved it. Milling also increased accessible surface area.

PHA, polyhydroxyalkanoate; PHB, poly(3-hydroxybutyrate); PHBV, poly(3-hydroxybutyrate-co-3-hydroxyvalerate); PBAT, poly(butylene adipate-co-terephthalate); PLA, poly(lactic acid); PP, polypropylene; PE, polyethylene; EVA, ethylene–vinyl acetate; PDLLA, poly(D, L-lactic acid); NR, natural rubber; PEG, polyethylene glycol; LAP, Laprol plasticizer; MA, maleic anhydride; PHA-g-MA, maleic anhydride–grafted PHA; TPF, tea plant fiber; t-TPF, treated (crosslinked) tea plant fiber; RH, rice husk; ENR, epoxidized natural rubber; OP, olive pomace; DDGS, distillers dried grains with solubles; AgNP, silver nanoparticles; WF, wood flour.

Across natural fillers, fibers, and agro-derived residue mixed PHA systems, degradation trends consistently scale with the ability of fillers to create hydraulically and biologically connected interfaces and porosity within the polymer matrix (Salim et al., 2012). For example, PHBV composites containing peach palm fibers showed an increase in mass loss from approximately 20% to 35–40% as filler content increased from 10 wt.% to 20 wt.% over 150 days in fertile soil (Batista et al., 2010). Similarly, PHBV blended with wood flour exhibited a near fivefold increase in degradation relative to neat PHBV (≈13% vs. ≈2.6%) after 360 days in moist outdoor soil (Chan et al., 2019). Comparable enhancements were observed with olive pomace, where PHBV/OP composites reached ~100% mass loss within 120 days, compared to ~91% for neat PHBV under identical conditions (Lammi et al., 2019). Similar trends have been observed with vine shoot fillers, where mineralization rates increased in PHBV systems, and removal of polyphenolic extractives further improved biodegradability by eliminating inhibitory effects on microbial activity (David et al., 2019). In outdoor sandy soil conditions, neat PHBV exhibited minimal degradation (~0.5% mass loss after 112 days), whereas PHBV-flax composites showed increased mass loss (~6%), which further increased in PHBV/PBAT-flax (~9%) and PHBV epoxidized natural rubber (ENR) (~17%) systems due to enhanced interfacial defects and microbial accessibility (Zaidi et al., 2019). In PHB systems reinforced with treated tea-plant fibers, composites containing maleic-anhydride-grafted PHA (PHA-g-MA/t-TPF) exhibited >30% degradation after 360 days, while ungrafted PHA-g-MA alone showed the lowest degradation (Batista et al., 2010). A similar pattern was observed in rice-husk composites, where PHA/rice husk films reached ~96% mass loss after 360 days, whereas acrylic acid grafting reduced degradation to ~90% despite increased polarity, indicating interfacial sealing (Wu, 2014). Madbouly et al. showed that incorporating distillers dried grains with solubles (DDGS) into PHB (90/10 wt%) increased mass loss by approximately six-fold relative to neat PHB over 168 days under outdoor burial conditions. The acceleration was attributed to DDGS acting as a hydrophilic, nutrient-rich filler that enhanced microbial colonization and promoted localized PHB breakdown (Madbouly et al., 2014). This ordering of studies here suggests that across fiber-filled systems, degradation acceleration arises when fillers convert the polymer matrix from a limited diffusion solid into a network of interconnected, water-filled interfacial zones that support sustained microbial residence and enzyme transport to PHA-rich domains. Modifications, e.g., graftings, that seal or isolate these interfaces, reintroduce transport limitations and suppress biodegradation despite increased polarity.

While fiber incorporation generally accelerates PHA degradation in thin films, this effect is reduced in pelletized or thick-section geometries due to transport limitations. Thomas et al. (2020) showed that neat P(3HB) pellets exhibited ~32% mass loss over 35 days, with only modest increases in peat (~44%), clay (~36%), and wood-flour (~33%) filled systems. In contrast, when processed as granules, degradation increased substantially, with P(3HB)/peat reaching ~54% mass loss, highlighting the dominant role of geometry in controlling biodegradation. Similar behavior was observed in flax-reinforced PHB/HV systems studied by Barkoula et al. (2010) where injection-molded pellets showed slow and spatially heterogeneous degradation over 360 days despite containing 20 wt.% flax fibers. In pelletized and thick-section PHA systems, fiber reinforcement does not translate into uniform bulk degradation because material geometry imposes strong transport constraints, and exposed surface area and diffusion length become the dominant controls on degradation rate. High thickness and dense packing limit water penetration into the interior, restricting microbial colonization and enzymatic activity to regions near the exposed surface. Consequently, measured mass loss in pelletized systems reflects degradation confined to surface accessible zones, while the interior remains largely unaffected over typical soil burial durations.

Formulation-induced changes in crystalline structure influence not only the extent of biodegradation but also the lag phase and mineralization kinetics. However, these intrinsic structural effects are strongly modulated by environmental conditions. For instance, under saturated soil conditions at 25 °C, both PHB and PHBV films have been reported to undergo complete degradation within 2 weeks, whereas the same materials showed only limited degradation over 6 weeks in soils maintained at lower relative humidity (~40%), highlighting the dominant role of moisture in regulating degradation kinetics (Kim et al., 2023).

When PHA is blended with other polymers, a consistent trend is observed across studies, where biodegradation is often phase-specific, and biodegradability is not necessarily transferred across phases. In blends of PHA with inert polyolefins such as Polypropylene (PP) or PP-co-PE (Polyethylene), the PHA phase typically exhibits high degradation (~90–100%), whereas the polyolefin phase remains essentially unchanged (<0.3%), resulting in partial material breakdown and persistent residues (Gonçalves et al., 2009; Rani-Borges et al., 2016). Similar behavior has been reported for blends containing ethylene-vinyl acetate (EVA) and other non-hydrolysable polymers, which form stable microdomains that persist in soil and restrict access to adjacent PHA regions (Fernandes et al., 2024). In contrast, when PHA is combined with other biodegradable polyesters, degradation behavior becomes more interactive. In these blend systems, PHA does not degrade as an isolated component, and blend architecture, phase continuity, crystallinity, and interfacial accessibility strongly influence the overall degradation outcome. PHA can facilitate the formation of localized defects that enhance microbial access to adjacent polymer domains. In PLA/PHB films, total mass loss reached 55–57%, which was higher than the degradation rate of PLA or PHB films taken individually (Jeszeová et al., 2018). In PHB/PBAT 55/45 wt.% blends, mineralization declined from near-incomplete degradation of neat PHB film to ~47% for a PHB/PBAT film (Fernandes et al., 2024). Although neat PBAT exhibited limited mineralization under the same conditions, the identification of PBAT-containing fungi, including *Purpureocillium* and *Aspergillus* species, indicates that PBAT degradation occurred only after initial microbial access was established through PHB domains (Fernandes et al., 2024). In ASTM D5988 soil respirometry systems, plasticized PHBV achieved approximately 35% mineralization over 200 days, while a PLA/PHBV (70:30) blend reached ~32%, indicating that blending and plasticization can modulate degradation depending on phase structure and environmental conditions (Muniyasamy et al., 2016). These results illustrate that plasticizer identity alone can shift PHB degradation kinetics, independent of soil biological activity (Bibers et al., 2001). In PDLLA (PLA (including poly(l-lactide) and poly(d,l-lactide)) containing systems, PEG addition restored partial biodegradability (~20 wt% loss) compared to PEG-free blends (<5%), therefore, also demonstrating that hydrophilicity and water transport can override otherwise unfavorable polymer chemistry (Wang et al., 2008).

Overall, formulation-driven effects on PHA degradation are highly context-dependent and do not operate uniformly across environments. The same material may degrade rapidly in moist, biologically active soils but persist in dry, coarse-textured, or microbially limited systems. Thus, degradation outcomes are jointly governed by soil conditions, including moisture, texture, microbial activity, and oxygen availability, alongside material design, and cannot be generalized without considering both factors.

#### 4.1.3 Molecular weight as a determinant

While PHA degradation in soil is predominantly surface-mediated, changes in molecular weight can influence material structure and indirectly affect degradation kinetics. Polymer molecular weight influences the progression of PHA degradation in soil, but should be distinguished from the primary surface-mediated mechanism that governs mass loss. In soil environments, reductions in molecular weight can occur prior to detectable mass loss, often due to limited abiotic hydrolytic chain scission within accessible regions of the polymer matrix. These scission events reduce average chain length and weaken the material mechanically, leading to embrittlement and microcrack formation (Boyandin et al., 2012). However, such changes do not directly result in bulk material removal. Instead, observable mass loss remains dependent on extracellular depolymerase activity at the polymer soil interface, where oligomers and soluble monomers are generated and subsequently assimilated. Thus, molecular weight reduction is best interpreted as a secondary, structure-modifying process that can facilitate surface erosion by increasing accessibility, rather than as the primary driver of degradation in soil (Dulac et al., 2025; Rajan et al., 2017). This sequence of early Mw decay followed by delayed mass loss is a recurring feature for biodegradable polymers (including PHAs) degradation in soils (Boyandin et al., 2012; Zambrano et al., 2020; Bonartsev et al., 2012), and explains why short-term studies have often captured molecular changes without corresponding weight loss.

However, as PHA degradation progresses beyond the initiation phase, molecular weight becomes a secondary factor, and crystallinity and lamellar morphology exert stronger control over long-term degradation kinetics. By the time degradation is evaluated at later stages, where mass loss is measurable, the polymer has already undergone extensive chain scission, and differences in molecular weight have largely converged. As a result, studies focusing on mass loss or long-term endpoints often fail to detect relationships that are mechanistically relevant but confined to early stages of degradation (Bandopadhyay et al., 2020b; Volova et al., 2017; Sridewi et al., 2006; Sintim et al., 2019a, 2019b, 2020). Most available studies documenting molecular weight decline do not isolate its effects from other variables such as processing history, film morphology, blend formulation, or mechanical properties. In addition, systematic evaluations under field or field-realistic soil conditions using PHAs engineered with controlled molecular weight ranges remain lacking. These gaps highlight the need for carefully designed studies that disentangle molecular weight from other structural variables to determine its significance in commercial agricultural applications.

#### 4.1.4 Influence of thickness, surface area, and geometry

PHA biodegradation in soils (and other environments) is fundamentally surface-mediated, meaning thickness, particle size, porosity, roughness, and surface area all govern how soil microorganisms colonize and depolymerize the material. Laboratory data consistently show that materials with the highest surface-area-to-volume ratio, such as in their powders and microparticle form, degrade substantially faster than films, while compact pellets exhibit the slowest degradation (Meereboer et al., 2020; Numata et al., 2009; Ong and Sudesh, 2016). This trend reflects not only differences in exposed surface area but also constraints on water penetration, enzyme diffusion, and microbial colonization within thicker or densely compacted geometries. Multiple studies illustrate this pattern clearly. In practical agricultural settings, this means that a mulch film hundreds of micrometers thick will degrade substantially more slowly than a fine powder or thin fiber of the same polymer composition.

In the work by Gutierrez-Wing et al. (2010) PHB powder achieved ~68% biodegradation within 12 days, whereas melt-pressed plates of PHB with thicknesses of 0.24–5.0 mm required a minimum of 19 days to reach comparable levels of biodegradation (Meereboer et al., 2020). Similarly, in soil microcosms, PHB microparticles lost mass ~4–5 times faster than corresponding films or pellets, owing to a more rapid moisture penetration, better oxygen availability, and easier enzymatic access (Volova et al., 2017). Another frequently cited comparison shows that PHB powder buried in soil was completely mineralized in under 90 days, whereas a 0.6 mm thin compression molded film lost only ~10% of its mass over the same test period, despite being of the same polymer grade (Tarazona and Lendlein, 2020). In the Boyandin et al. (2013) study, thin films degraded far more rapidly than compact pellets, and P(3HB) consistently degraded faster than P(3HB/3 HV) copolymer. After approximately 12 months, P(3HB) exhibited near-complete mass loss (~98%), whereas pellets showed only partial degradation (8–55%). These findings imply that practically thinner PHA films or structured geometries that increase effective surface area offer a more reliable route to field degradation than compositional tuning alone. Although thin films can gradually accelerate their own degradation through pitting, cracking, and edge erosion induces increased surface area over time, these processes typically occur only after an initial induction period and remain restricted to the outermost layers of the material.

The processing route can further modify geometry-driven degradation behavior. In contrast to native intracellular granules, once PHAs are extracted from cells, the granule-associated proteins and endogenous depolymerases are removed, thereby eliminating the biological constraints that normally limit crystal growth. When the polymer is subsequently melted, extruded, or solvent-cast, the chains are fully mobilized and then recrystallized on cooling. In this unconstrained state, PHA chains readily assemble into larger, thicker lamellae and more fully developed spherulites than those found *in vivo*, resulting in degradation behavior governed primarily by physicochemical structure rather than biological interfaces. In this unconstrained state, PHA chains readily assemble into larger, thicker lamellae and more fully developed spherulites than those found *in vivo* (Tarazona and Lendlein, 2020). Consequently, processed PHAs typically exhibit 55–80% crystallinity and relatively higher hydrophobicity, with the exact value governed by cooling rate, molecular weight distribution, and HV content (Mondol et al., 2025; Eesaee et al., 2022). These processing-induced morphological changes have direct implications for soil biodegradability. Thicker lamellae and well-developed spherulites limit water ingress, slow the diffusion of depolymerases, and raise the energetic barrier for ester bond cleavage. In a study by Jain and Tiwari (2015), solvent-cast films of PHB in chloroform were shown to lose only approximately 64% of their mass in a 6-month garden soil experiment, despite being chemically simple and nominally biodegradable. Sevakumaran et al. (2019) could also only achieve a mass reduction of about 43% in a solvent case of P(3HB) films (varying thickness from 0.3 to 6 mm) in the tropical soils and environment of Malaysia.

Conversely, processing routes that generate the sheer abundance of accessible surface or less ordered surfaces (e.g., quenching, electrospinning, or high shear extrusion) can initiate bulk disintegration, induce amorphocity, facilitate deep microbial colonization, promote faster moisture uptake, and accelerate the onset of enzymatic depolymerization. In a recent comparative soil burial study, it was demonstrated that there was a > 90% loss of mechanical strength in electrospun PHB after short-term soil exposure, compared to ~49% loss in a solvent case PHB film (Gasparyan et al., 2023). Specifically, the crystallinity of the PHB film decreased from 58.5 to 28% over 90 days, and although embrittled, the film remained macroscopically intact. In contrast, an electrospun PHB mat of the same thickness exhibited only a modest crystallinity decrease (56.3–45%) after just 8 days, yet the material had already fragmented into small pieces. The electrospun sample lost all its tensile strength (to 0.16 N) and underwent catastrophic fragmentation, whereas the film retained >6 N of strength before testing and remained far more robust throughout exposure (Gasparyan et al., 2023). Together, these results indicate that electrospun nanofibers of PHA with open porosity between strands, and intrinsically very rough surfaces, can convert a surface-initiated mechanism into a rapid, volume-wide disintegration process.

### 4.2 Abiotic factors affecting biodegradation

#### 4.2.1 Moisture (soil water content)

**Figure 3:**
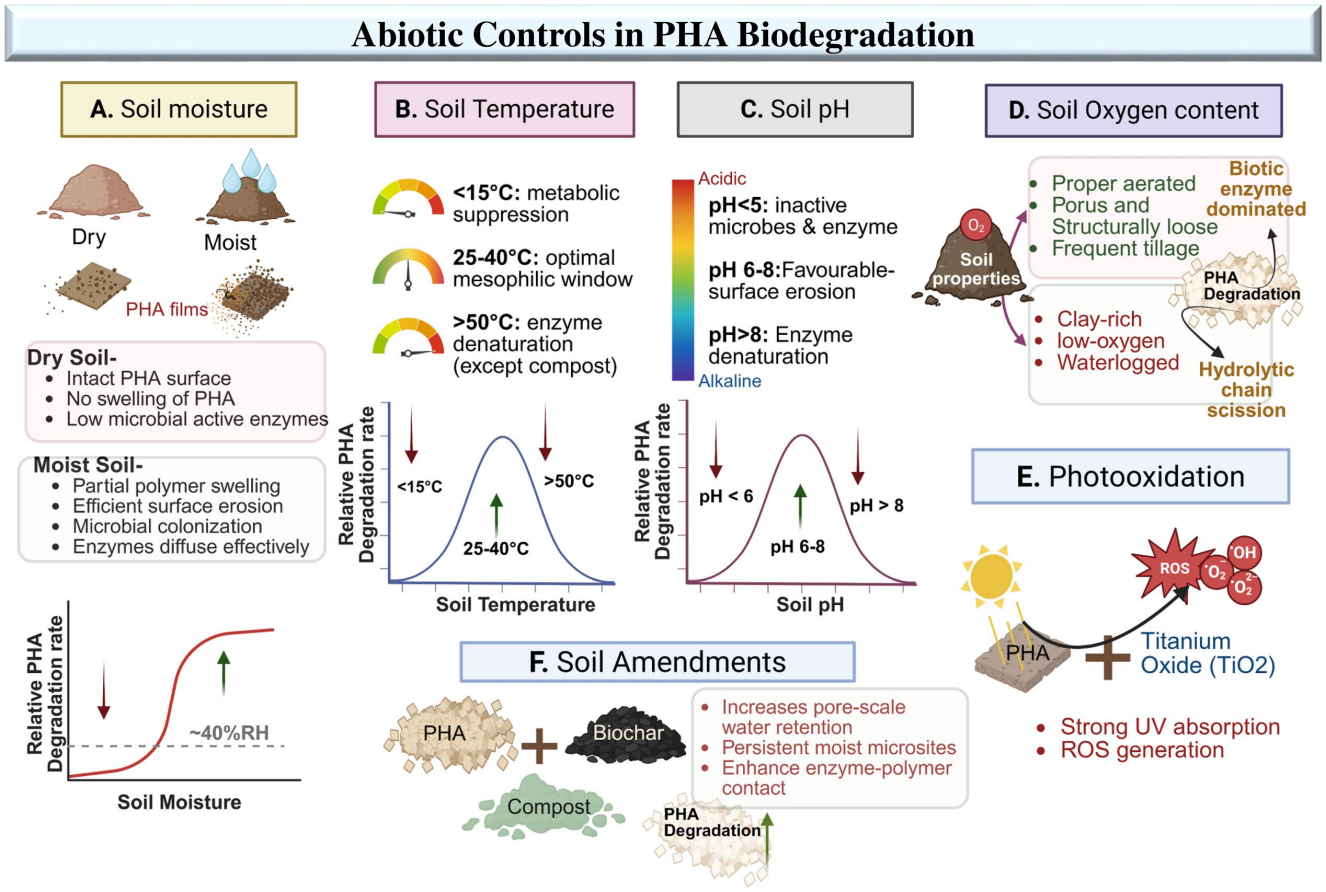
The figure highlights how the abiotic factors, such as soil moisture **(A)**, temperature **(B)**, pH **(C)**, oxygen **(D)** availability, light exposure **(E)**, and soil amendments **(F)**, influence the PHA degradation. These abiotic conditions regulate polymer swelling, enzyme activity, and degradation pathways, thereby modulating the rate and extent of PHA breakdown in soil environments.

Soil moisture is perhaps the single most influential factor for PHA degradation (Figure 3A). Microbial activity and hydrolysis require water. Several studies document that saturated or wet soils accelerate PHA breakdown, while dry soils greatly impede it. In a well-designed microcosm study, Kim et al. (2023) exposed PHB and commercial PHBV films to agricultural soil held at 25 °C under two humidity regimes, 100% relative humidity (RH) (water saturated level) and 40% relative humidity. Under saturated conditions, both polymers lost mechanical integrity within 3 days and were essentially completely degraded within 2 weeks, with a sharp decrease in molecular weight and modest changes in crystallinity over time. Under 40% RH, by contrast, neither PHB nor PHBV showed appreciable changes in mass, melting behavior, or molecular weight over 6 weeks. In long-term field tests in Vietnam, PHB films in the wetter site lost >90% in 10 months, whereas at a drier site, only ~47% mass was lost in 12 months (Boyandin et al., 2013). Another comparative analysis of P(3HB) films degradation in two contrasting soils—humid, organic-rich Siberian chernozem soil, and the much drier, nutrient-poor ferralitic soil in India, demonstrated how strongly soil moisture governs PHA breakdown. P(3HB) films buried in the moist chernozem degraded almost twice as fast (half-life of ~65 days) as those placed in the dry red soil (half-life of ~126 days), even though both soils contained microbes capable of producing PHA depolymerizing enzymes (Prudnikova et al., 2024). Practically, this means PHA mulches or implants will persist much longer in arid conditions, and below a critical moisture threshold, PHA can remain almost inert on an agronomic timescale, even when degradable and surrounded by microbes. Hydrophilic additives and blend partners can partially circumvent this moisture barrier by increasing water uptake and accelerating chain scission. Freier et al. (2002) showed that citrate esters increased water uptake of PHB and speed up their hydrolytic molecular weight loss, particularly in the early stages of degradation.

In agricultural soil, moisture is not constant, and there are drying-rewetting cycles, spatial heterogeneity, and differences in texture and pore structure (Kim et al., 2023). Under dry conditions, the already low water uptake of crystalline-rich PHA films becomes even more limiting, and the polymer surface does not swell; enzymes cannot diffuse effectively, and microbial colonization is reduced. Even under moist soil conditions, PHA fragments may become embedded within microaggregates or pore regions where oxygen diffusion is limited, which further restricts aerobic depolymerase activity. As a result, the lamellar structure that was originally advantageous for intracellular carbon storage now acts as a kinetic barrier to biodegradation in soil, especially where PHA-degrading microbes or specific depolymerases are not abundant. For agricultural systems, this has clear management implications. PHA mulch films are far more likely to degrade predictably in well-irrigated vegetable beds, high-organic-matter soils, or fine-textured loamy soils that retain moisture. However, coarse, sandy, or drought-prone soils may support only slow, pulsed degradation linked to rewetting events. More generally, decomposition ecology studies suggest that fungi are relatively more important decomposers under lower soil moisture conditions. In contrast, bacteria become more active and competitive under higher moisture regimes (with the added fungal activity). This trend could be highly applicable to the breakdown of PHB or its composites and a critical determining factor for the slower rates of degradation in low moisture content soils (Ullah et al., 2023).

Although moisture clearly regulates enzymatic access to PHA surfaces, most evidence derives from simplified laboratory systems that fail to capture the coupled effects of soil structure, oxygen diffusion, and hydrological variability in agricultural fields, limiting field-scale extrapolation. One of the few studies to examine PHA degradation under natural soil hydrological regimes is by Lim et al. (2005). Although conducted in non-agricultural tropical soils, this *in situ* burial study provides critical field-relevant insight into how moisture interacts with polymer properties. Under identical wet conditions, including stream adjacent and water saturated mangrove soils, scl-PHB films fragmented extensively, whereas mcl-PHA films exhibited only limited surface erosion and minimal molecular change (Lim et al., 2005). This contrast demonstrates that increased moisture alone does not guarantee rapid PHA degradation. Instead, moisture amplifies degradation only when polymer morphology and mechanical properties permit fragmentation and enzymatic access. This characteristic is significant for translating moisture effects in agricultural soils, where irrigation or rainfall may accelerate degradation of some PHA formulations but not others.

#### 4.2.2 Soil temperature

Temperature is one of the strongest abiotic controls on PHA biodegradation because it can simultaneously govern microbial metabolism, enzyme activity, and water dynamics (Figure 3B). Under composting conditions (≥ 50 °C), PHAs can degrade rapidly, often within 6–12 weeks. A few of the long-term field trials of soil-biodegradable films now confirm that this discrepancy matters. For example, Griffin-LaHue et al. (2022) modeled EN 17033-compliant mulch films under Mediterranean field conditions at Washington State University’s Northwestern Washington Research and Extension Center in Mount Vernon, WA (48°43′24″N, 122°39′09″W, elevation above sea level 6 m). With an Average soil temperature at a 5-cm depth from May to November of 16 °C (range 4–22 °C) and from December to April of 8 °C (range 1–16 °C), the study estimated that 90% biodegradation of a PHA/PLA blended mulch film would require roughly 21–58 months, i.e., systemically longer than the 24-month requirement under constant 20–28 °C test conditions. This estimate reflects the behavior of a blended system and may not be representative of pure PHA materials, whose degradation kinetics can vary depending on composition and structure. These findings highlight an important limitation of current ISO/ASTM soil-biodegradation tests, which do not fully capture realistic agricultural temperature regimes or seasonal variability. As a result, standardized test conditions may overestimate degradation rates for certain PHA-based materials under field conditions. Integrating temperature cycling and anchoring laboratory data to multi-season field trials will be essential for generating more realistic predictions of PHA degradation in soil environments.

#### 4.2.3 Soil pH

Soil pH regulates PHA degradation primarily by controlling microbial activity and extracellular depolymerase function, rather than by promoting direct chemical hydrolysis under typical soil conditions (Figure 3C). Although ester bonds in PHAs can theoretically undergo acid or base-catalyzed hydrolysis, experiments show that in sterile buffer conditions, PHA films are remarkably resistant to non-enzymatic hydrolysis under neutral and mildly acidic aqueous conditions, even over extended incubation periods of up to 98 days (Volova et al., 2010). In contrast, PHB is highly susceptible to degradation under strongly alkaline conditions (e.g., pH 13), where hydroxyl anions effectively break the ester bonds (Yu et al., 2005). This again indicates that rapid PHB degradation in natural environments such as soil and compost is driven primarily by microorganisms and their associated PHA depolymerases, rather than by biotic hydrolysis alone.

PHA depolymerases generally exhibit narrow pH optima, most commonly under neutral to mildly alkaline conditions (pH 7–8), with activity declining sharply at acidic (<6) or strongly alkaline (>9) conditions (Jendrossek and Handrick, 2002). When soil pH shifts outside this optimal range, the depolymerases may lose catalytic activity, and the colonizing microbes may reduce their metabolic activity. As a result, PHA films in acidic peat or highly alkaline calcareous soils degrade more slowly, even when microbial abundance is high (Patil et al., 2023). This trend is further supported in the study by (Prudnikova et al. (2024) where PHB degradation in neutral Siberian chernozem soil (pH 7.0) occurred nearly twice as fast as in slightly acidic Indian ferralitic red soil (pH ~ 5.2).

In addition to these bulk-soil pH effects, PHA degradation may also generate localized pH feedback at the polymer-soil interface. As PHB or PHBV chains are enzymatically cleaved, degradation products such as 3-hydroxybutyric acid and other organic acids can be released (Meereboer et al., 2020). In soils with low buffering capacity, these acidic intermediates may lower the pH locally by up to 1–1.5 units, even when measured bulk soil pH remains near neutral. This localized acidification can temporarily reduce depolymerase activity and slow further degradation, creating microsites where PHA fragments persist under rainfall, irrigation, diffusion, or bioturbation that redistributes the accumulated acidity.

#### 4.2.4 Oxygen and redox conditions

Oxygen availability is a primary environmental determinant of PHA biodegradation in agricultural soils because PHA depolymerase and downstream metabolic pathways are overwhelmingly associated with aerobic heterotrophs (Figure 3D). Under well-aerated conditions, extracellular depolymerases cleave ester bonds to release 3-HAs, which are rapidly oxidized through *β*-oxidation and the TCA cycle, yielding CO_2_ and water (Jendrossek and Handrick, 2002). Consequently, aerated, structurally loose soils, those with high microporosity, frequent tillage, or strong faunal activity, consistently show PHA mineralization faster than compacted or waterlogged soils. In contrast, low-oxygen or anaerobic environments demonstrate slow degradation, not because PHA is chemically recalcitrant under reduced conditions, but because the microbial guild capable of anaerobic polyester depolymerization is both taxonomically limited and metabolically slower. Recent investigations confirm that polyhydroxyalkanoates (PHAs), including PHB and PHBV, exhibit significantly restricted biodegradation under strict anaerobic soil conditions compared to aerobic environments (Quecholac-Piña et al., 2020). In oxygen-deprived soil or aquatic systems, the initial enzymatic hydrolysis required to cleave the polymer backbone becomes the primary rate-limiting step, leading to negligible mass loss compared to the rapid fungal-driven degradation observed in aerated controls. This environmental sensitivity underscores that the presence of oxygen is a critical determinant of PHA degradation kinetics, regardless of identical polymer chemistry. Even when anaerobic breakdown does occur under controlled conditions in anaerobic digestion systems, PHA degradation can still be modest. For instance, Gómez and Michel (2013) proclaim that during anaerobic digestion for 50 days, only 20–25% of PHB/PHV was converted to biogas (CH_4_ + CO_2_), confirming that anaerobic methanogenic consortia can depolymerize PHAs, but with low efficiency relative to aerobic pathways.

Weng et al. (2013) examined the degradation behavior of extrusion case P(3HB,4HB)/PLA blend films buried in pond soil under planted conditions (fescue) for 4 months, explicitly contrasting aerobic (20 cm) and anaerobic (40 cm) burial depths. Under aerobic conditions, degradation followed a clear PHA content-driven trend, wherein PHA-100 [P(3HB,4HB)] > PHA-75 [P(3HB,4HB)/PLA, 75/25] > PHA-50 [P(3HB,4HB)/PLA, 50/50] > PHA-25[P(3HB,4HB)/PLA, 25/75] > PLA. In contrast, under anaerobic conditions, the trend reversed, and PLA material degraded more than either PHA or any of the PHA/PLA blends. SEM and FTIR analysis confirmed that PHA degradation proceeded primarily via microbiologically mediated surface erosion, yielding low molecular weight acids, whereas PLA degradation under anaerobic burial was dominated by non-biological bulk hydrolytic chain scission. These results in this study also indicate that PHA degradation slows under reduced microbial activity at depth (Weng et al., 2013).

Overall, it is evident that differences in soil redox conditions can fundamentally shape how PHA-based materials degrade under agricultural conditions. PHA mulch films are likely to degrade predictably in aerated, intermittently tilled, or drip-irrigated systems, but may persist longer in compacted soils, clay-rich fields with poor drainage, or seasons dominated by waterlogging. Current ISO/ASTM tests, conducted under highly oxygenated conditions, do not capture these redox dynamics. A more complete understanding will require long-term field trials that explicitly monitor oxygen diffusion, soil structure, and water table fluctuations as co-variables influencing PHA turnover. In addition, the behavior of PHAs in flooded agricultural systems remains insufficiently explored; environments such as rice paddies or periodically saturated soils are likely to exhibit slower or pulsed degradation, with breakdown occurring primarily during transient aerobic intervals.

#### 4.2.5 Ultraviolet (UV) exposure and photooxidation

Although soil burial limits light exposure, PHAs used in agricultural films frequently experience partial or intermittent sunlight during deployment, making photo-oxidation a relevant modifier of degradation behavior (Figure 3E). UV irradiation can induce chain scission in PHAs via Norrish type I and II reactions and peroxide radical formation, thus weakening the polymer matrix before or concurrent with soil contact (Sridewi et al., 2006; Jeszeová et al., 2018; Yew et al., 2006). In some of the research articles, the authors have recommended the incorporation of titanium dioxide (TiO_2_) as a photocatalyst into P(3HB) films to accelerate photodegradation under sunlight (Sridewi et al., 2006; Altaee et al., 2016; Yew et al., 2006). However, field-relevant evidence indicates that TiO_2_ introduction does not uniformly accelerate PHA degradation but instead introduces a context-dependent shift in degradation control. In the outdoor burial study by Yew et al. (2006), PHB-TiO2 films degraded more rapidly than neat PHB under high-irradiance, low microbial conditions (62,400–115,300 lux; ~ 13,000 CFU g^−1^). This trend was reversed in shaded, microbially active soils (2,530–11,800 lux; ~ 27,000 CFU g^−1^), where neat PHB outperformed PHB-TiO_2_ (Yew et al., 2006). Importantly, soil pH was comparable across sites (pH 4.4–4.6), isolating light exposure and microbial abundance as the dominant controlling variables. These results demonstrate that TiO_2_ promotes photocatalytic chain scission when photon flux is high but suppresses biologically mediated depolymerization under conditions where microbial colonization would otherwise dominate degradation. Mechanistically, this suppression arises from two coupled effects. First, under UV or visible light, TiO2 generates reactive oxygen species (ROS) at the polymer surface, including hydroxyl radicals and superoxide species, which can oxidize microbial cell membranes and extracellular enzymes, reducing microbial viability and enzymatic activity at the polymer soil interface. Second, TiO_2_ incorporation alters surface physicochemical properties of PHA films, including surface energy, roughness uniformity, and wettability, which can reduce stable microbial adhesion and biofilm establishment, processes that are prerequisite for effective enzymatic depolymerization (Sridewi et al., 2006; Altaee et al., 2016). As a result, rather than acting as a universal accelerator, TiO_2_ introduces a switching behavior between abiotic and biotic degradation regimes. This trade-off is particularly relevant for agricultural mulch films that transition between exposed and buried states.

An opposing strategy involves UV stabilizers, which are widely used to extend the functional lifetime of polymer films during field exposure. For example, benzotriazole-based UVA absorbers (UV stabilizers) effectively limit photo-oxidation, but they can also slow biodegradation. Tian and Wang (2020) provided a systematic study, where PHA/PBAT mulch films containing UV stabilizers retained structural integrity longer under sunlight but exhibited slower mass loss following soil incorporation. This work also provided a similar understanding that UV stabilizers protect PHAs against UV irradiation, but at the cost of slowing down the soil degradation of the film (Wang et al., 2022). Together, these studies reinforce that photooxidation does not act independently of soil biology. This therefore highlights the need to treat PHA not as uniformly biodegradable materials, but as systems whose environmental performance is jointly governed by formulation of chemistry, exposure history, and placement.

#### 4.2.6 Soil amendments

Nutrient availability, particularly nitrogen and phosphorus, plays an underappreciated but conceptually important role in determining PHA biodegradation in soils. PHAs are essentially pure carbon-energy storage polymers; their depolymerization and subsequent mineralization require microbes to invest in fresh biomass, extracellular depolymerases, and sustained active respiration, all of which demand adequate supplies of N, P, and micronutrients (Sridewi et al., 2006; Sevakumaran et al., 2019). Consequently, PHA inputs can locally elevate soil C: N ratios beyond the range (~20–30%) typically favorable for microbial growth and plant nutrition. Under such a stoichiometric imbalance, depolymerization may initiate but fails to propagate, leading to long induction phases and incomplete degradation even for intrinsically labelled PHA formulations (Brtnicky et al., 2022). This mechanism explains why slow degradation is often observed in poor nutrient agricultural soils despite favorable temperature and moisture.

Evidence from agricultural soil studies indicates that application of nitrogen-containing organic amendments, such as mature composts, digestates, or manure-derived materials, can partially buffer PHA-induced nutrient bottlenecks that otherwise suppress enzyme synthesis and microbial turnover (Figure 3F). These amendments can supply both mineral and organically bound nitrogen, supply large and more functionally diverse microbial communities (beyond just introducing a PHA specialized degrader), generally increase soil pH, and possibly counterbalance the effect of 3-HB, therefore converting PHA from an isolated carbon source into one embedded within a metabolically functional soil matrix. In a study reported by Brtnicky et al. (2025a), compost and digestate added to soil consistently reduced residual PHA fractions in soil from 0.37% in unamended soil to ~0.22–0.23%. In this sense, amendments act less as accelerators and more as enablers of continuity, which allows degradation to proceed beyond the initiation phase. However, Witt et al. (2024) demonstrated that both polymer composition and polymer-to-urea ratio strongly govern nitrogen release kinetics in a sandy soil-based column. Pure urea released ~100% of its N within 24 h, whereas PBAT containing 33% urea released ~70% within 24 h and reached ~100% by day 3. In contrast, PHA containing 12% urea and PBAT containing 20% urea showed slower, biodegradation-mediated release, reaching only 30 and 50% respectively, after 21 days in soil, consistent with sustained N availability and the absence of any initial abrupt release.

In any field or soil microcosm, soil organic matter (SOM) can amplify this effect by further stabilizing moisture and sustaining decomposer populations between wetting events. Typically, SOM-rich soils have been reported to host larger and more diverse decomposer communities, including *Pseudomonas, Bacillus, Streptomyces,* and fungi (the phylum level dominated by *Ascomycota*), all capable of synthesizing extracellular PHA depolymerases (Šerá et al., 2020). In a 35-day empirical soil burial experiment by Volova et al. (2017) involving agro-chernozem soil (SOM-rich heavy loam), solvent-casted films of P(3HB), its copolymer (3HB/3 HV, and P(3HB/4HB), as well as blends containing mcl-PHA (P(3HB/3HH×), all achieved ≥90% mass loss. This rapid mass loss of PHAs coincided with the strong enrichment of organotrophic degraders, including ~1.6 × 10^7^ CFU g^−1^ of copiotroph bacteria, presence of 36 PHA-degrading strains with dominance of *Pseudomonas, Stenotrophomonas,* and Var*iovorax*, a ~ 3-fold increase in fungi, expansion of prototrophs (↑ 1.8 x), and suppression of oligotrophs (↓8.3×). Together, these shifts indicate that SOM-rich soils mobilize microbial guilds that are capable of exploiting PHA-derived carbon and compress degradation timescales from months to weeks.

Biochar has been widely studied in agricultural systems as a structural and soil conditioning amendment. Biochar’s aromatic, recalcitrant, and hydrophobic structure makes it largely unavailable for microbial metabolism, but from a PHA depolymerization perspective, it is highly effective in stabilizing moisture retention at the pore and aggregate scale. In a study by Gonçalves et al. (2018) PHBV biodegradation was examined in soil systems amended with silver nanoparticles (AgNPs), which are known to influence microbial activity and soil–polymer interactions. The inclusion of AgNPs led to a measurable reduction in overall PHBV degradation, consistent with partial suppression of microbial colonization and enzymatic activity in the surrounding soil. To evaluate whether soil amendments could modulate this response, biochar was introduced as an additional variable. Notably, even in the presence of AgNPs, biochar increased PHBV mineralization relative to the unamended control by 2–3 times. Moreover, this enhancement occurred without evidence of increased microbial biomass or nutrient-driven stimulation. Although the mechanisms are not resolved directly in the available studies, they are consistent with well-established soil physical evidence showing that biochar increases water retention, pore connectivity, and the persistence of moist microsites, particularly useful under fluctuating moisture regimes (Edeh et al., 2020). Oxygen-containing functional groups (–OH, –COOH, –C=O) on biochar surfaces increase wettability and promote hydrogen bonding with water molecules. It is worth noting that these effects are expected to be context-dependent. Biochar is most effective as a co-amendment for PHA biodegradation in moisture-limited, coarse-texture, drought-prone, or low-SOM soils, where it can prove itself to be the most effective strategy to stabilize pore-scale hydration and enzyme-polymer contact in soil. In contrast, in fine-textured or organic-rich loam soils where moisture continuity, nutrient retention, and microbial biomass are already high (Koller et al., 2025), the incremental effect of biochar in causing PHA mineralization is expected to be minimal.

### 4.3 Biotic controls on PHA biodegradation

Although PHA chemistry sets the potential for biodegradability, the actual breakdown of PHA in environments is controlled by the presence, abundance, and activity of microorganisms that express extracellular PHA depolymerases (PhaZ) and broader specificity esterases/lipases that can initiate surface erosion under field conditions (Arcos-Hernandez et al., 2012). Traditionally, the presence of this biotic control has been studied at two complementary levels: (i) metagenome scale evidence that soils are major reservoirs of PHA depolymerase genetic potential, and (ii) field and pot soil measurements showing that agriculturally managed soils frequently contain large degrader populations and exhibit short lag phases before measurable mass loss.

In a comprehensive analysis of 3,078 metagenomes, Viljyakainen and Hug predicted 13,869 putative PHA depolymerase genes, with the soil-derived metagenomes having the largest share of 78% in this signal (reported as the greatest densities (78%) amounting to 10,835 predicted PHA depolymerases spanning 24 phyla) (Viljakainen and Hug, 2021). This evidence supports soil as a major reservoir of PHA-degrading capacity. However, a key limitation, highly relevant to agriculture, is that these metagenome compilations do not explicitly resolve cropland versus non-cropland soils. As a result, even though agricultural soils fall within the broader terrestrial category, metagenomic quantification of PHA depolymerases and other polymer-degrading enzymes that are explicitly ‘cropland resolved’ remains largely absent. Unfortunately, direct field resolved characterization of active degrader communities in real agricultural soils also remains limited, and most current knowledge is still derived from targeted isolations, burial assays, or controlled pot experiments (Boyandin et al., 2012).

Briese, Jendrossek, and Schlegel quantified PHB-degrading bacteria ranging from 9.2 × 10_5_ to 1.30 × 106 g^−1^ in garden and field soils. Their numbers were supported with some of the clearest enumerations from Song et al. (2003), Wang et al. (2005) studies, who measured aerobic PHB-degrading microorganisms across distinct agricultural soils. They reported approximately 10^5^ degrader cells per gram of dry soil. Specifically, they reported 4.3 × 10^5^ degraders g^−1^ in fertile garden soil, 5.06 × 10^5^ degraders g^−1^ in paddy soil, and 8.71 × 10^5^ degraders g^−1^ in alluvial farm soil, and importantly showed that mass loss of PHB films strongly correlated with degrader abundance (Song et al., 2003; Wang et al., 2005). Boyandin et al. (2013) further provide rare quantitative estimates of microbial abundance on PHA surfaces, showing that bacterial populations on P(3HB) and P(3HB/3 HV) films and pellets reached ~10^8^–10^9^ CFU g^−1^, which is one to two orders of magnitude higher than counts in the surrounding bulk soil (~10^6^–10^7^ CFU g^−1^), thereby directly demonstrating strong microbial enrichment within the PHA-associated plastisphere.

**Figure 4:**
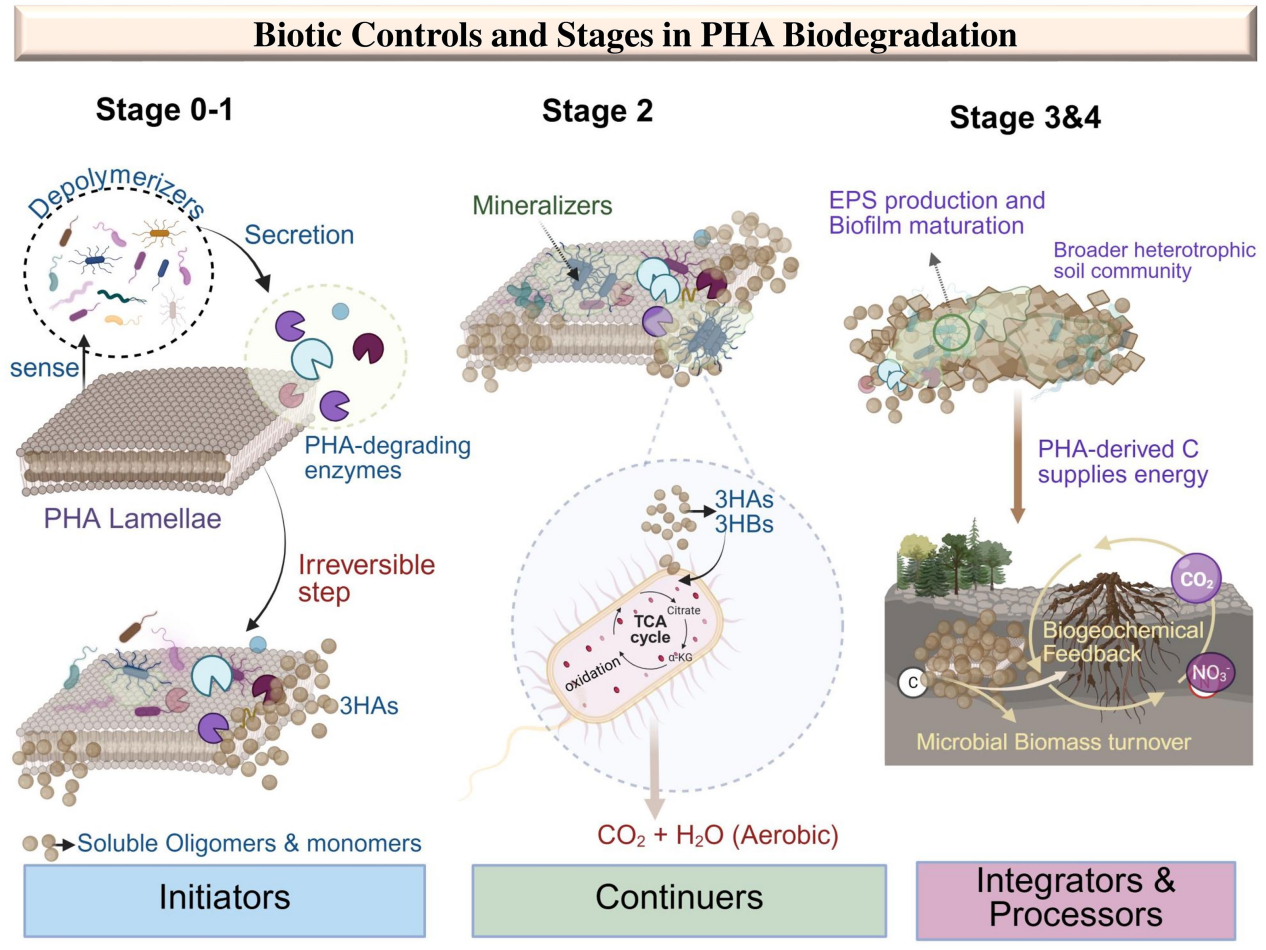
Conceptual, probabilistic model illustrating the staged biodegradation of P(3HB) in agricultural soils, from microbial induction lag through depolymerization, cross-feeding, biofilm maturation, and ultimate carbon mineralization.

Soil surveys and film colonization studies conducted over the past decades consistently show that species belonging to Firmicutes, Actinobacteria, Bacteroidetes, and Proteobacteria commonly emerge as the dominant degradative lineages associated with PHB and PHBV films (Table 2). In here, a recurring set of bacterial and fungal genera is consistently observed in association with PHA degradation, although individual taxa often exhibit overlapping and context-dependent functional capacities (Paloyan et al., 2025; Boyandin et al., 2012; Jendrossek and Handrick, 2002; Sharma et al., 2019; Islam et al., 2025; Chhetri et al., 2025). From a mechanistic perspective, however, it remains useful to provisionally segregate these genera according to the stage at which their activity is most likely to exert the greatest influence on the degradation process, recognizing that such roles are neither exclusive nor static. This conceptual staging of microbial activity is summarized schematically in Figure 4. In this framework, certain genera are most plausibly positioned as:

1. Early-stage contributors or Initiators (Stages 0–1). Initiators are microorganisms that are likely responsible for triggering PHA degradation. Previous studies corroborate the fact that degradation of PHA specimens starts after a lag period (Stage 0), during which microorganisms are assumed to recognize the polymer surface of the specimens, attach and adapt to the PHA surface as the substrate, and induce and excrete extracellular PHA depolymerases (Volova et al., 2017). In Stage 1, they carry out the first irreversible step of degradation at the PHA surface by hydrolyzing the exposed PHA polymer molecules into soluble oligomers and monomers. These microbes are expected to be relatively low in abundance but functionally critical, as they control the onset and initial rate of degradation. For instance, *Pseudomonas* remains a model genus for well-characterized extracellular PHA depolymerases, including enzymes with strong activity on scl-PHAs and, in a smaller subset of lineages, activity on mcl-PHAs. This makes *Pseudomonas* a credible ‘primary depolymerizer’ in many soil systems because it can convert solid polymer into soluble oligomers/monomers that other organisms can then assimilate (Numata et al., 2009; Jendrossek and Handrick, 2002). *Streptomyces* and other filamentous actinobacteria (e.g., *Amycolatopsis, Thermobifida, Mycobacterium, Terrabacter, Terracoccus,* and *Nocardia*) are repeatedly implicated because their filamentous growth and robust extracellular enzyme secretion enable surface colonization and sustained erosion of both scl and mcl-PHAs (often visible as pitting, cracking, and fragmentation in burial assays) (Sharma et al., 2019; Chhetri et al., 2025; Agapov et al., 2025; Gangoiti et al., 2012; Martínez et al., 2015; Tokiwa and Calabia, 2004; Volova, 2015). *Rhodococcus* is another genus whose members are frequently associated with hydrophobic surface adhesion and broad esterase/lipase systems, supporting a role as a persistent colonizer that can exploit polymer-associated carbo and co-metabolize breakdown products (Paloyan et al., 2025; Zampolli et al., 2022). Fungal contributors under agricultural or pot soil conditions are also supported, particularly under moist regimes where hyphal penetration increases polymer enzyme contact. Filamentous fungi such as *Penicillium, Aspergillus, Trichoderma,* and *Fusarium* are reportedly active taxa capable of secreting extracellular esterases, lipases, and cutinase-like enzymes that contribute to PHB surface erosion, and, in some cases, measurable mass loss (Fernandes et al., 2024; Ong et al., 2017; Volova, 2015).
2. Continuers (Stage 2: Cross-Feeding and Community Expansion). Continuers are microorganisms that are unlikely to degrade solid PHA directly but are well adapted to rapidly assimilate the soluble degradation products released by initiators. During Stage 2, these organisms are expected to dominate carbon uptake by consuming oligomers and monomeric 3HAs, leading to rapid population growth and microbial succession. Continuers likely include copiotrophic heterotrophs with high substrate uptake rates and short generation times, such as *Burkholderia*, *Cupriavidus* (*Ralstonia*), Var*iovorax*, *Comamonas*, *Delftia*, *Achromobacter*, *Acinetobacter*, *Alcaligenes*, *Ensifer*, *Mitsuaria*, *Roseateles*, and *Roseomonas* (Paloyan et al., 2025; Volova et al., 2017; Boyandin et al., 2012; Fernandes et al., 2024; Jendrossek and Handrick, 2002; Sharma et al., 2019; Volova, 2015; Ong et al., 2017). Their activity sustains and amplifies overall carbon flux from PHA by preventing the accumulation of degradation products and maintaining favorable thermodynamic conditions for continued depolymerization. Importantly, this division of labor framing is consistent with many soil plastic colonization studies, bringing forth the fact that only a fraction of the biofilm community is true depolymerizers, while a large fraction are secondary consumers sustained by released oligomers and monomers.
3. Integrators (Stage 3: Biofilm Maturation and Coupled Metabolism): As degradation progresses, some microorganisms are expected to function as integrators by contributing to stable biofilm formation and metabolic coupling between depolymerization and uptake. These microbes may include taxa that persist from earlier stages as well as organisms that are particularly effective at surface colonization, extracellular polymeric substance (EPS) production, and spatial organization within biofilms (Gupta et al., 2019). Genera commonly associated with biofilm formation and surface-associated lifestyles, such as *Paenibacillus*, *Chitinophaga*, *Pseudoxanthomonas*, *Xanthomonas*, and *Stenotrophomonas*, are likely contributors to this role (Volova et al., 2017; Mangwani et al., 2021). Their function is not to initiate degradation, but to optimize enzyme retention, local substrate recycling, and microbial interactions that stabilize degradation rates.
4. Processors and Feedback Drivers (Stage 4: Carbon Fate and Soil Feedbacks): In the final stage, a broad suite of soil microorganisms acts as processors, channeling PHA-derived carbon into respiration, microbial biomass turnover, and soil biogeochemical feedbacks (Cagle, 2021). These organisms are expected to mineralize most PHA-carbon to CO₂ during routine metabolism, with only a small fraction transiently incorporated into biomass. Genera such as *Acidovorax* and members of the broader heterotrophic soil community are likely to participate primarily at this stage, where the dominant function is regulation of ecosystem-level carbon and nutrient cycling rather than polymer breakdown itself (Watts and Lloyd, 2012). Under nitrogen-limited conditions, some members of this group may act as feedback drivers by using PHA-derived carbon as an energy source while mining nitrogen from native SOM, thereby inducing positive priming effects.

**Table 2:** Genus-level ecological traits inferred enzymatic capacities and indicative involvement across stages of PHA biodegradation in soil systems.

Genus	Taxonomic group	Domain	Representative species reported	Distinguishing features/notes	Key references
*Absidia*	Mucoromycota	Fungi	*A. glauca*	Saprotrophic filamentous fungus reported in PLA/PHB blends; likely contributes via extracellular esterases and hyphal surface penetration rather than dominant depolymerization (S1–S3).	Jeszeová et al. (2018)
*Acinetobacter*	Gammaproteobacteria (Moraxellaceae)	Bacteria	*A. calcoaceticus, A. baumannii, A. johnsonii, A. radioresistens* *A. lwoffii*	Surface-associated heterotroph; relies on esterases and dehydrogenases to oxidize soluble PHA-derived hydroxyacids rather than intact polymer; downstream assimilation and respiration (S2–S4).	Boyandin et al. (2012), Jendrossek and Handrick (2002), Sharma et al. (2019)
*Actinomucor*	Mucoromycota	Fungi	*A. elegans,*	Reported PLA/PHB blend degrader; enzyme system not fully characterized; likely contributes to early surface erosion in mixed communities (S1–S2).	Jeszeová et al. (2018)
*Achromobacter*	Betaproteobacteria	Bacteria	*A. xylosoxidans*	Copiotrophic soil bacterium adapted to soluble substrates; utilizes low-molecular-weight degradation products via dehydrogenases and *β*-oxidation pathways (S2).	Volova et al. (2017)
*Acidovorax*	Betaproteobacteria	Bacteria	*A. facilis, A. temperans*	Widespread heterotrophs involved in organic carbon oxidation; enzyme repertoire dominated by central carbon metabolism rather than extracellular hydrolases, suggesting participation mainly in terminal mineralization of PHA-derived carbon; Probable role: S4.	Volova et al. (2017)
*Alcaligenes*	Betaproteobacteria	Bacteria	*A. faecalis, A. latus*	Classic PHB degraders; heterotroph capable of utilizing diverse low-molecular-weight substrates. Likely expresses esterases and organic-acid utilization enzymes. Its role is not polymer attack but secondary processing of degradation products via acetyl-CoA and TCA pathways. Probable role: S2–S4.	Volova (2015)
*Agrobacterium*	Alphaproteobacteria	Bacteria	*A. tumefaciens; A. radiobacter*	Exceptional surface conditioning and biofilm formation via exopolysaccharides; Likely facilitates polymer wetting and microbial succession rather than driving depolymerization itself; Probable role: S1.	Volova (2015)
*Amycolatopsis*	Actinobacteria	Bacteria	*A. mediterranei, A. orientalis*	Filamentous actinobacterium known for extracellular hydrolases and surface colonization; cutinase- or esterase-like activity may support early depolymerization of solid PHA substrates; Probable role: S1.	Joyyi et al. (2017), Tokiwa and Calabia (2004)
*Arthrobacter*	Actinobacteria	Bacteria	*A. oryzae*	Stress-tolerant soil bacterium enriched during later degradation stages; primarily assimilates soluble breakdown products (S2–S3).	Jeszeová et al. (2018)
*Aureobasidium*	Dothideomycetes	Fungi	*A. pullulans,*	Polymorphic saprotrophic fungus with esterase and lipase activity; contributes to surface colonization and intermediate degradation (S1–S3).	Jeszeová et al. (2018)
*Aspergillus*	Eurotiomycetes	Fungi	*A. fumigatus*, *A. niger, A. teereus; insuetus*	Saprotrophic filamentous fungus with strong secretion of esterases, lipases, and cutinase-like enzymes; hyphal growth facilitates access to solid polymers, making it relevant to early depolymerization and later surface-associated degradation; Probable role: S1–S3.	Fernandes et al. (2024), Tokiwa and Calabia (2004), Ong et al. (2017)
*Bacillus*	Firmicutes	Bacteria	*B. megaterium*, *B. subtilis, B. pumilus, B. cereus, B. licheniformis, B. thuringiensis, B. flexus, B. coagulans*	Fast-growing, spore-forming soil bacterium with extracellular enzyme secretion; EPS production and biofilm formation; depolymerase-like esterases and biofilm retention likely support both polymer attack and sustained degradation; Probable role: S1-S3.	Volova et al. (2017), Jeszeová et al. (2018), Volova (2015)
*Brevibacillus*	Firmicutes	Bacteria	*B. brevis; B. borstelensis; B. laterosporus*	Strong extracellular enzyme secretion coupled with spore-forming resilience; One of the few Firmicutes repeatedly linked to active PHA depolymerase production in soils; Probable role: S1.	Volova (2015)
*Bjerkandera*	Basidiomycota	Fungi	*B. adusta*	Saprotrophic white-rot fungus producing oxidative enzymes (laccases, peroxidases); reported in PLA/PHB blends. Likely modifies polymer surfaces and matrix structure, enabling secondary microbial access rather than direct PHA depolymerization (S2–S3).	Jeszeová et al. (2018)
*Burkholderia*	Betaproteobacteria	Bacteria	*B. cepacia* complex*, Paraburkholderia xenovorans*	Highly versatile soil bacteria; capable of both intracellular PHA cycling and extracellular degradation; often enriched in rhizosphere-associated soils, suggesting a role in rapid assimilation of soluble PHA breakdown products during community expansion; Probable role: S2.	Joyyi et al. (2017), Jendrossek and Handrick (2002)
*Chitinophaga*	Bacteriodetes	Bacteria	*C. pinensis*	Polymer-surface colonizer; contributes via extracellular hydrolases in mixed consortia rather than dominant depolymerization; Probable role: S3.	Volova et al. (2017)
*Cladosporium*	Dothideomycetes	Fungi	*C. subcinereum*	Common soil saprotroph reported in PLA/PHB blends; likely contributes to secondary surface erosion (S2–S3).	Jeszeová et al. (2018)
*Clonostachys*	Sordariomycetes	Fungi	*C. rosea*	Reported in PHB/PBAT systems; contributes to secondary attack following initial PHA access (S2–S3).	Fernandes et al. (2024)
*Comamonas*	Betaproteobacteria	Bacteria	*C. testosteroni, C. acidovorans*	Known polyester degraders; efficient utilization of short-chain hydrolysis products; commonly co-occurs with other Betaproteobacteria on biodegradable plastics, specialized in extracellular polymer turnover; hydrolases and EPS interactions suggest relevance in biofilm-mediated processing rather than initial depolymerization; Probable role: S3.	Volova (2015)
*Cupriavidus (Ralstonia)*	Betaproteobacteria	Bacteria	*C. necator* (*R. eutropha*)*, C.basilensis, C. pauculus*	Well known for intracellular PHA metabolism; presence of PHA synthase/depolymerase systems supports efficient routing of monomers into central metabolism and storage-mobilization cycles; Probable role: S2–S4.	Jendrossek and Handrick (2002), Ong et al. (2017), Volova et al. (2017), Volova (2015)
*Delftia*	Betaproteobacteria	Bacteria	*D. acidovorans*	Frequently enriched in soil burial studies; often part of mixed consortia rather than dominant degraders; efficient scavenger of PHA oligomers; Probable role: S2.	Volova et al. (2017), Boyandin et al. (2012)
*Duganella*	Betaproteobacteria	Bacteria	*D. zoogloeoides; D. phyllosphaerae*	Rapid responder to sudden increases in soluble carbon; Probable role: S3.	Volova (2015)
*Ensifer*	Alphaproteobacteria	Bacteria	*E. meliloti*	Rhizosphere-adapted heterotroph with flexible carbon metabolism; likely contributes to assimilation and respiration of soluble PHA-derived carbon; Probable role: S1–S4.	Volova et al. (2017)
*Fusarium*	Sordariomycetes	Fungi	*F. solani, F. oxysporum*	Plant-associated fungi contributing to PHA breakdown in crop soils, saprotrophic fungus producing extracellular esterases and oxidative enzymes; hyphal penetration supports early surface erosion and later biofilm-like interactions; PLA/PHB blend degrader Probable role: S1–S3.	Fernandes et al. (2024), Jeszeová et al. (2018), Volova (2015)
*Ilyobacter*	Fusobacteriota (anaerobic bacteria)	Bacteria	*I. polytropus; I. tartaricus*	Strictly anaerobic fermenter capable of converting PHA-derived substrates to short-chain acids; Relevant primarily in buried, compacted, or water-logged soils where oxygen is limiting; Probable role: S4	Volova (2015)
*Lysinibacillus*	Firmicutes	Bacteria	*L. sphaericus, L. fusiformis*	Soil-borne Firmicutes with moderate but persistent polymer hydrolysis; often isolated from agricultural and compost-amended soils; Spore-forming bacterium with extracellular enzymes and EPS production; esterase-like activity and persistence under stress suggest participation in early degradation and biofilm stabilization; Probable role: S1–S3.	Volova et al. (2017)
*Mitsuaria*	Betaproteobacteria	Bacteria	*B. adusta*	Copiotrophic soil bacterium adapted to soluble carbon uptake; metabolic profile supports cross-feeding on PHA-derived monomers; Probable role: S2.	Volova et al. (2017)
*Mortierella*	Mucoromycota	Fungi	*M. alpina, M. elongata*	Saprotrophic soil fungus enriched in agricultural soils; it utilizes soluble oligomers and low-molecular-weight carbon released during PHA degradation. Contributes to downstream assimilation and mineralization rather than primary polymer depolymerization (S2–S4).	Jeszeová et al. (2018)
*Mycobacterium*	Actinobacteria	Bacteria	*M. smegmatis; M. fortuitum; M. gilvum*	Waxy, mycolic-acid-rich cell envelope promotes strong adhesion to hydrophobic polymer surfaces; Exceptional ability to colonize hydrophobic solids; degradation is slow but persistent and surface-controlled; Probable role: S1–S2.	Volova (2015)
*Nocardia*	Actinobacteria	Bacteria	*N. farcinica, N. asteroides*	Slow-growing filamentous actinobacterium with affinity for hydrophobic substrates; oxidative enzymes and hydrolases suggest involvement in early polymer surface degradation; capable of sustained PHB depolymerization over long burial periods; Probable role: S1–S3.	Volova et al. (2017), Jendrossek and Handrick (2002)
*Pseudomonas*	Gammaproteobacteria	Bacteria	*P. lemoignei*, *P. stutzeri, P. fluorescens, P. putida, P. mendocina, P. aeruginosa, P. alcaligenes, P. citronellolis, P. denitrificans*	Model genus for extracellular PHB depolymerases (PhaZ); enzymes are among the best characterized, dominant degraders in many soil systems; strong biofilm/EPS production; capable of contributing across depolymerization and biofilm-mediated degradation phases; Probable role: S1–S3.	Volova et al. (2017), Jendrossek and Handrick (2002), Volova (2015), Ong et al. (2017)
*Pseudoxanthomonas*	Gammaproteobacteria		*P. mexicana*	Surface-associated heterotroph with extracellular enzymes and biofilm affinity; likely involved in enzyme retention and cooperative degradation within biofilms; Probable role: S3.	Volova et al. (2017)
*Paenibacillus*	Firmicutes	Bacteria	*P. polymyxa, P. alvei*	Plant-associated soil bacteria; EPS-producing soil bacterium with extracellular hydrolases; specialization lies in biofilm structuring and localization of enzymatic activity near polymer surfaces; Probable role: S3.	Boyandin et al. (2012), Jeszeová et al. (2018), Tokiwa and Calabia (2004)
*Penicillium*	Eurotiomycetes	Fungi	*P. chrysogenum, P. funiculosum, P. simplicissimum*	Filamentous fungus with strong secretion of esterases, lipases, and cutinases; hyphal systems enhance polymer accessibility during early and intermediate degradation; Probable role: S1–S3.	Zaidi et al. (2019), Fernandes et al. (2024), Jeszeová et al. (2018), Volova (2015)
*Paecilomyces*	Ascomyete	Fungi	*P.* var*iotii*	Cutinase- and esterase-producing fungus; supports early and intermediate degradation (S1–S3)	Zaidi et al. (2019)
*Rhodococcus*	Actinobacteria	Bacteria	*R. ruber, R. erythropolis, R. opacus*	Strong surface adhesion and hydrophobic cell envelopes; prominent plastisphere colonizers; rich esterase/oxygenase repertoire; frequently associated with degradation of solid, hydrophobic polymers; PLA/PHB blend degrader; Probable role: S1.	Jendrossek and Handrick (2002), Volova (2015), Jeszeová et al. (2018)
*Rhodoferax*	Betaproteobacteria	Bacteria	*R. ferrireducens; R. antarcticus*	Flexible redox metabolism, including activity under low-oxygen conditions; Bridges aerobic and micro-oxic zones where PHA breakdown products accumulate; Probable role: S3–S4	Volova (2015)
*Roseateles*	Betaproteobacteria	Bacteria	*R. depolymerans*	Soil bacterium reported in polymer-degrading contexts; esterase-like activity suggests involvement in early depolymerization, though evidence remains limited; Probable role: S1–S2.	Volova et al. (2017)
*Roseomonas*	Alphaproteobacteria	Bacteria	*R. mucosa*	Low-nutrient-adapted heterotroph; metabolic traits align with late-stage respiration of soluble carbon rather than polymer degradation; Probable role: S4.	Volova et al. (2017)
*Saccharothrix*	Actinobacteria	Bacteria	*S. espanaensis*	Filamentous actinobacterium reported in PHB blends; likely surface-associated depolymerizer (S1–S2)	Jeszeová et al. (2018)
*Streptomyces*	Actinobacteria	Bacteria	*S. exfoliatus*, *S. venezuelae, S. griseus, S. lividans, S. thermoviolaceus, S. venezuelae; S. coelicoflavus*	Filamentous growth enables aggressive surface colonization; synthesize widest range of extracellular secreted enzymes; well-suited for degradation of solid biopolymers in early stages; Probable role: S1	Jendrossek and Handrick (2002, Chhetri et al. (2025), Zaidi et al. (2019), Fernandes et al. (2024), Jeszeová et al. (2018), Tokiwa and Calabia (2004), Volova (2015)
*Stenotrophomonas*	Gammaproteobacteria	Bacteria	*S. maltophilia*	Opportunistic soil bacterium with strong extracellular enzyme secretion; frequently detected in PHB clear-zone assays; likely contributes to biofilm-associated processing and late-stage carbon turnover; Probable role: S3–S4	Joyyi et al. (2017), Jendrossek and Handrick (2002), Volova (2015)
*Streptacidiphilus*	Actinobacteria	Bacteria	*S. melanogenes*	Acidophilic actinobacterium reported for mcl-PHA; contributes to surface depolymerization under acidic conditions (S1–S2)	Joyyi et al. (2017)
*Terrabacter*	Actinobacteria	Bacteria	*T. tumescens; T. terrae*	Adapted to carbon-poor soils with efficient recycling of complex organic residues; Likely exploits partially oxidized PHA fragments rather than initiating depolymerization; Probable role: S2–S3	Volova (2015)
*Terracoccus*	Actinobacteria	Bacteria	*T. luteus; T. ginsenosidimutans*	Extremely stress-tolerant oligotroph with low metabolic demand; Survives late-stage, nutrient-depleted conditions after primary degraders exhaust labile carbon; Probable role: S3	Volova (2015)
*Thermobifida*	Actinobacteria	Bacteria	*T. fusca, T. alba*	Thermophilic actinomycetes; active in warm soils and compost-amended agricultural systems; enzymes often thermostable; specialization supports efficient depolymerization of recalcitrant polymers; Probable role: S1.	Tokiwa and Calabia (2004)
*Trichoderma*	Sordariomycetes	Fungi	*T. reesei; T. harzianum,*	Aggressively saprotrophic fungus with diverse extracellular hydrolases; hyphal growth enables effective attack on solid polymers and participation in biofilm-like matrices; Probable role: S1–S3.	Jeszeová et al. (2018), Tokiwa and Calabia (2004)
*Variovorax*	Betaproteobacteria	Bacteria	*V. paradoxus, V. boronicumulans*	Frequently enriched on biodegradable polymer surfaces; can degrade PHA copolymers; specializes in metabolizing low-molecular-weight degradation products; supports continuation of degradation through efficient monomer assimilation; Probable role: S2.	Zhou et al. (2023), Volova et al. (2017), Jendrossek and Handrick (2002), Ong et al. (2017)
*Xanthomonas*	Gammaproteobacteria	Bacteria	*X. campestris*	Plant-associated soil bacterium; EPS-producing, surface-associated bacterium; contributes primarily to biofilm architecture and enzyme immobilization rather than direct polymer cleavage; Probable role: S3.	Boyandin et al. (2012), Jendrossek and Handrick (2002)

Stage designations (S1–S4) refer to conceptual phases of PHA degradation used in this study: S1, early depolymerization of solid PHA; S2, cross-feeding and community expansion via soluble degradation products; S3, biofilm maturation and coupled metabolism; and S4, terminal carbon mineralization and soil feedback. Stage associations are indicative and non-exclusive.

A persistent ecological constraint that matters directly for agricultural deployment is the strong bias of soil communities toward scl-PHA turnover relative to mcl-PHA. In contrast to the extensive literature on scl-PHAs, microbial degradation of mcl- PHAs remains comparatively underdeveloped. Current evidence is concentrated in a limited number of experimentally validated environmental isolates, mainly from soil, compost, activated sludge, and aquatic habitats (Mandic et al., 2019). The most consistently reported mcl-PHA degraders belong to *Pseudomonas*, particularly *Pseudomonas fluroscens* GK13, with additional reports involving *Streptomyces*, Comamonas, *Janthinobacterium, Undibacterium, and related taxa* (Colak and Güner, 2004; Park et al., 2001). Collectively, these studies show that biologically relevant microbial resources for mcl-PHA turnover do exist, but their diversity, ecological distribution, and functional robustness in agricultural soils remain insufficiently defined. Mechanistically, like other PHA molecules, mcl-PHA degradation also proceeds through surface-mediated microbial colonization of the polymer, followed by secretion of extracellular depolymerases or lipase-like hydrolases that cleave ester bonds at the polymer interface (Sharma et al., 2019; Woolnough et al., 2008). This extracellular strategy is necessary because mcl-PHAs are hydrophobic, insoluble polymers that cannot be directly transported into microbial cells (Sharma et al., 2019; Woolnough et al., 2008; Woolnough et al., 2010).

At the molecular level, the best-characterized mcl-PHA depolymerases are secreted serine hydrolases. The extracellular P(3HO) depolymerase from *Pseudomonas fluorescens* GK13 contains the conserved lipase-like motif Gly-X-Ser-X-Gly, with the catalytic serine shown to be essential for activity. This enzyme is strongly hydrophobic, which likely promotes interaction with mcl-PHA surfaces, and it preferentially hydrolyzes medium-chain-length substrates rather than short-chain-length PHAs. Other extracellular PHA depolymerases, such as the PHBH-degrading enzyme identified in *Undibacterium*, contain a signal peptide, catalytic domain, linker region, and substrate-binding domain, highlighting a modular architecture that may improve adsorption to polymer surfaces and catalytic efficiency (Morohoshi et al., 2020). These observations suggest that microbes degrading mcl-PHA combine polymer-surface recognition, extracellular ester hydrolysis, and substrate specificity for medium-chain-length monomers. The hydrolytic products released from mcl-PHA are typically soluble oligomers and monomers, which can then be assimilated as carbon sources by the degrading organism (Morohoshi et al., 2020). For example, the P(3HO) depolymerase of *P. fluorescens* GK13 predominantly generated dimeric products during hydrolysis, whereas the PHBH depolymerase from *Undibacterium* released monomers and oligomers up to pentamers, consistent with at least partial endo-type cleavage of the polymer chain (Morohoshi et al., 2020). Taken together, these studies support a coherent but still narrow model of mcl-PHA degradation in soil: specialized microorganisms attach to the polymer surface, secrete extracellular hydrolases, cleave the chain into soluble fragments, and assimilate the released hydyroxyalkanoates.

The key limitation is not the absence of mcl-PHA-degrading biology, but the limited evidence that such biology is abundant, active, and reliable in agricultural soils. Unlike scl-PHA degradation, which can be supported by a broader microbial reservoir, mcl-PHA degradation appears to depend on a smaller and more specialized microbial resource base. This distinction is critical for agricultural use, because mcl-PHA-rich films may not mineralize predictably unless soils contain suitable degraders or are managed to supply them through compost inoculation, bioaugmentation, or formulation strategies that improve microbial access. Thus, broader isolate discovery, standardized strain level validation, and direct testing in agricultural soils remain essential before mcl-PHAs can be confidently evaluated for field deployment.

Evidence for microbial PHA degradation also exists in thermophilic soil and compost environments, but these systems primarily illustrate mechanistic potential rather than agricultural relevance. Studies have shown that specialized thermophilic fungi and bacteria can rapidly degrade PHB at elevated temperatures (Al Hosni et al., 2019), yet such thermal conditions are rarely encountered in cropland soils and therefore have limited bearing on field performance. In agricultural systems, the dominant controls on PHA degradation remain in mesophilic temperature ranges, soil moisture availability, and microbial accessibility at the soil-polymer interface. Consequently, thermophilic studies are best viewed as boundary cases that define the upper limits of microbial degradation capacity, rather than predictors of PHA behavior in managed agricultural fields.

#### 4.3.1 Role of soil Fauna in the degradation of PHA

Soil fauna needs recognition as an active contributor to the degradation of biodegradable polymers through a combination of physical, chemical, and biological mechanisms that extend beyond those occurring in bulk soil environments. Khaldoon et al. (2022) highlight that earthworms influence polymer breakdown both directly, via ingestion and gut passage, and indirectly, by altering soil structure, aeration, moisture distribution, and microbial community composition in casts and burrow linings (Qi et al., 2018). Experimental evidence has demonstrated that earthworm gut transit can fragment biodegradable plastics such as polylactic acid (PLA), producing smaller particles with increased surface area and altered morphology, thereby enhancing susceptibility to subsequent microbial mineralization (Wang et al., 2022).

Beyond mechanical fragmentation, the physiological conditions within the earthworm gut provide a microenvironment that may significantly accelerate polymer degradation compared to strictly soil-based processes. Earthworm guts exhibit dynamic pH gradients depending on species and gut region, influencing ester bond hydrolysis in aliphatic polyesters. Additionally, earthworm digestive fluids contain an array of endogenous enzymes, including esterases and lipase-like enzymes. While primarily involved in organic matter digestion, these enzymes may contribute to the early-stage initiation in the context of PHA degradation. Additionally, the gut hosts a dense and metabolically active microbiota distinct from surrounding soil communities, broadening the initial scale for breakdown of scl- and mcl-PHA. This gut-associated microbiome has been shown to persist in earthworm casts, which are typically enriched in bioavailable nitrogen and labile organic matter due to digestive processing, thereby forming a localized microcosm of microbial activity that can further degrade PHA following excretion, analogous to the continuation phase discussed before (Khaldoon et al., 2022).

The combined effects of mechanical comminution during gut passage, transient exposure to digestive enzymes, and inoculation with polymer-degrading microbial consortia suggest that earthworm-mediated processes may lower the activation barrier for PHA degradation relative to degradation occurring solely in soil matrices. In agricultural soils with high earthworm activity, repeated ingestion and redistribution of PHA-containing material could therefore substantially enhance degradation rates by coupling gut-mediated preprocessing with accelerated microbial mineralization in casts and surrounding soil.

Despite growing recognition of fauna-mediated effects on biodegradable polymer fragmentation and transformation, these processes have not been systematically studied under controlled laboratory conditions or in realistic agricultural field settings, particularly for PHAs. Most existing studies focus on short-term ingestion experiments or extrapolate degradation behavior from bulk soil assays, without explicitly decoupling gut-mediated mechanisms from soil-only microbial degradation pathways. Investigating these effects in well-defined laboratory systems and validated agricultural soil mesocosms could provide more holistic insights into the environmental fate of biodegradable plastics used in agriculture.

## 5 Assessment of biodegradation methods

According to the International Standardization Organization (ISO) definition, plastics need to undergo significant changes in their chemical structure by the activity of naturally occurring microorganisms to be considered biodegradable. On the other hand, the European Standardization Committee (CEN) is stricter and believes biodegradable plastic needs to be converted into microbial metabolic products (Fernandes et al., 2020). As such, polymers biodegrade differently in industrial composting facilities, soil, freshwater, or marine habitats. Accordingly, the existing test methods focus on aerobic or anaerobic biodegradation in specific environments (Ruggero et al., 2019). Concerning aerobic biodegradation tests in soil/compost, the most used standard testing methods for laboratories are the ASTM D5988-18 (Sintim et al., 2020; D5988, A, 2018), ISO 17556 (17556, I, 2019), and EN 17033 (17033, E, 2018). Indeed, EN 17033 for “Plastics - Biodegradable mulch films for use in agriculture and horticulture Requirements and test methods” has been created using the methodology of ISO 17556. ISO 17556 is the core “soil mineralization” method often pointed to when a material is claimed to be biodegradable in soil, because it measures ultimate biodegradation via carbon conversion to CO_2_ (rather than just fragmentation) (17556, I, 2019). In practice, the polymer is mixed with a defined soil (or soil-like inoculum), incubated under controlled aerobic conditions, and biodegradation is quantified from the cumulative CO_2_ evolved related to the theoretical carbon content of the test material (Zhou et al., 2023). ASTM D5988 is the closest ASTM analogue to ISO 17556 and also quantifies aerobic biodegradation in soil by measuring evolved CO_2_ over time (Briassoulis et al., 2020). Since the polymer deterioration generally occurs through surface erosion, due to microbial activity, scanning electron microscopy (SEM) has often been considered a valuable tool to study colonization and biodegradation of PHA films (Briassoulis et al., 2020).

Because garden soils or compost may hold microorganisms with this ability, it has been considered an environment with an excellent capacity for degrading PHA as well (Fernandes et al., 2020). It is important to note that neither ISO nor ASTM soil biodegradation standards prescribe a single, field-representative soil composition, nor do they require the exclusive use of agricultural soils. Instead, these standards deliberately allow a wide range of soil media, resulting in substantial variability across studies and complicated interpretation for agricultural applications. Under ISO 17556, biodegradation may be assessed using either (i) natural soil collected from a defined location, or (ii) a standardized artificial soil consisting of approximately 70% sand, 10% clay, 16% natural soil, and 4% mature compost (with an organic C: N ratio not exceeding 40:1). This formulation was designed to ensure reproducibility and microbial activity, but it does not resemble most cropped soils in texture, organic matter content, aggregation, or microbial structure. By contrast, ASTM D5988 permits the use of natural soil alone, provided it meets broad compositional criteria (typically ~30% clay, 30% silt, and 40% sand) and maintains a soil organic C: N ratio between 10 and 20 (D5988, A, 2018; 17556, I, 2019). As a consequence, many biodegradation studies that are nominally compliant with ISO or ASTM standards rely on agricultural field soils, garden soils, or amended substrates, often without harmonized reporting of soil texture, organic matter content, microbial biomass, or management history. In addition, although current guidelines permit polymer testing across multiple material formats, they provide limited guidance on critical dimensional parameters, such as film thickness and particle size, that can strongly influence degradation outcomes (Albright III and Chai, 2021). Furthermore, standard test conditions typically impose constant temperatures (often 25–28 °C), continuous aeration, and highly controlled environments, while excluding key field-relevant processes including drying-rewetting cycles, soil compaction, root activity, and seasonal microbial dormancy. Therefore, while this flexibility enables broad applicability, it systematically overestimates the reliability and predictability of biodegradation in real agricultural fields. This gap is not accidental; it reflects a broader historical emphasis on waste management and compatibility of plastics, rather than agronomic fate after field use.

Test duration and termination criteria represent a second major limitation (Albright III and Chai, 2021). Under ISO17556, a test is considered invalid if the positive reference material fails to reach 60% degradation; under ASTM D5988, the threshold is 70%. Both standard reject tests if biological activity in blank reactors varies by more than 20%. Importantly, both standards define test completion based on the attainment of a ‘plateau phase’, rather than a fixed degree of mineralization. ISO 17556 recommends a nominal duration of 6 months but allows extension up to 2 years if degradation continues, whereas ASTM D5988 specifies no minimal or maximum test duration. This approach makes reported biodegradation outcomes highly sensitive to experimental timing. The longer a test is allowed to proceed, the higher the observed degree of biodegradation. This has been demonstrated explicitly for PHB. Briassoulis et al. (2020) reported ˃90% biodegradation of PHB in both natural and standard soils after 120 days, following ISO/ASTM protocols, whereas Sevakumaran et al. (2019) terminating their experiment after 35 days, reported only ~50% biodegradation for the same polymer. These discrepancies arise not from material differences but from differences in test duration and plateau interpretation, severely limiting inter-study comparability. Materials may therefore be classified as biodegradable, despite degrading too slowly to disappear within one or multiple cropping cycles. Critically, both ISO 17556 and ASTM D5998 are silent on soil health, ecological impact, and agronomic consequences of biodegradation. Neither does the standard require assessment of microbial community shifts, nutrient immobilization, phytotoxicity, or impacts on soil biota during or after polymer degradation. This omission is particularly critical for PHAs, whose degradation products can transiently alter soil C: N balance and microbial activity. Thus, current methods can certify biodegradability without evaluating whether degradation compromises soil function.

The European standard EN 17033 represents the most agriculture-oriented regulatory framework currently available for assessing the functional biodegradability of plastic mulch films in soil. Unlike ISO 17556 and ASTM D5988, which are generic soil biodegradation tests, EN 17033 was explicitly developed for plastic films used in agricultural and horticultural applications, and it attempts to address several deficiencies of earlier standards. Methodologically, EN 17033 introduces three major advances. First, it defines a clear biodegradation endpoint that mulch films must achieve ≥90% biodegradation under aerobic conditions within a maximum period of 2 years. This time-bound criterion directly reflects agricultural realities, where residues persisting beyond one or two cropping cycles become agronomically unacceptable. Second EN 17033 mandates the use of natural agricultural or forest topsoil, tested at 20–28 °C, rather than artificial or highly mineral standard soils. Third, as mentioned, it integrates ecotoxicological and soil health assessments, requiring demonstration that biodegradation does not negatively affect plant growth (OECD 208) or soil microbial activity (ISO 15685) (Hayes and Markus, 2018). Thus, by coupling biodegradation performance with ecological safety and a defined timeframe, EN17033 moves closer to agricultural relevance. Importantly, it does not apply to mulch films that are being removed from the fields after use.

However, despite these improvements, EN 17033 still fails to fully capture the complexity and variability of real agricultural fields, and several structural limitations remain. First, although EN17033 requires ‘natural agricultural or forest soil’, it also does not specify soil texture, organic matter content, microbial biomass, nutrient status, or management history. Second, testing at 20–28 °C still approximates mesophilic conditions favorable for microbial activity, but it does not represent the seasonal temperature dynamics experienced by agricultural soils (Meereboer et al., 2020). In many temperate cropping systems, soil temperatures remain below 15 °C for much of the year, while in arid or tropical systems, surface soil briefly exceeds 40 °C. EN 17033 does not account for thermal cycling, diurnal fluctuations, or seasonal dormancy, all of which can slow biodegradation or fragment it into episodic pulses. Even under 17,033 compliance, PHA mulch films may still degrade too slowly in cold, dry, nutrient-poor, or intensively managed soils. Third, the 2-year window, while agriculturally meaningful, still masks degradation kinetics, and it does not mandate reporting of degradation rates, lag phases, or seasonal pauses. Therefore, for instance, a material that degrades steadily over 18 months and one that remains intact for 16 months before rapidly.

mineralizing would both meet the standard, despite vastly different field implications (CSN, 2018). For farmers, prolonged persistence followed by late-stage fragmentation may be operationally unacceptable, even if the final mineralization threshold is eventually reached. Fourth, the inclusion of plant (OECD 208) and microbial (ISO 15685) tests is a major strength of EN 17033, yet these assays are short-term and endpoint focused. They do not capture transient soil effects such as nitrogen immobilization, shifts in microbial functional guilds, suppression of nitrifiers, or changes in rhizosphere interactions that may occur during plastic degradation (Bettas Ardisson et al., 2014). For carbon-rich polymers like PHAs, these transient effects can influence crop nutrition and soil functioning even if no long-term toxicity is observed. Therefore, most critically, EN 17033 does improve confidence that PHAs will not persist indefinitely or harm soil health, but it remains a laboratory-based proxy for field performance that does not ensure field disappearance within a single cropping cycle.

Existing standards, such as ASTM D5988-18, ISO 17556, and EN 17033, provide valuable baseline methods for evaluating biodegradation in soil. However, their ability to predict the field degradation of agricultural PHA materials remains limited because they are primarily designed around controlled laboratory conditions, not the variable conditions experienced for mulch films and other soil contact materials after deployment. For agricultural PHA materials, these tests should be supplemented with field-relevance modules that capture how mulch films and soil compact materials actually experience the field, i.e., the soil-to-soil variability, fluctuating temperature (Griffin-LaHue et al., 2022; Pischedda et al., 2019), wet-dry cycles, changing oxygen diffusion, and biological soil type (Huo et al., 2024), prior surface weathering before burial, heterogeneous microbial communities, and possible persistence of residue or fragments. As summarized in Table 3, these conditions are not intended to replace mineralization-based testing, but to make biodegradation results more predictive of agricultural performance by linking mineralization to soil context, material ageing, and residue fate. Moreover, as summarized in Table 4, even though standards exist and have already been defined, no single standard and no single method is enough to answer all important questions about the biodegradation behavior of polymers (including PHAs) in complex environments such as agricultural soils. Most biodegradation standards quantify mineralization through CO₂ or biogas evolution, whereas physical disintegration, residue persistence, and impacts on plants or soil organisms are evaluated using separate protocols and remain in isolation. This limitation is not a shortcoming of individual tests, but the inherent complexity of biodegradation as a multi-end point process. Table 4 is, therefore, provided to support integrated interpretation by linking common research and regulatory questions to the specific questions required to answer them.

**Table 3:** Proposed field relevance modules to strengthen biodegradation testing of agricultural PHA materials.

Limitation in current testing	Specific modification recommended	How to implement it	What it tells us
Constant laboratory temperature does not represent field soil conditions	Add temperature-cycling treatments or report degradation using thermal time	Test at realistic day–night or seasonal temperature cycles; additionally, report biodegradation per accumulated degree-days above a biologically active threshold	Determines whether slow degradation reflects polymer persistence or reduced microbial activity under cold or fluctuating field temperatures
Fixed moisture does not capture rainfall, irrigation, or drought cycles	Add wet–dry cycling modules	Compare constant optimal moisture with repeated wetting–drying cycles that mimic irrigation/rainfall and drying periods	Shows whether PHA degradation is stable under realistic moisture variation or only under ideal wet laboratory conditions
A single artificial or standard soil cannot represent agricultural variability	Use a minimum panel of contrasting agricultural soils	Include at least three soils differing in texture, organic matter, pH, moisture-holding capacity, management history, and microbial activity; report soil source and basic properties	Identifies whether degradation is broadly reproducible across farmland soils or dependent on a specific soil’s biological and physicochemical capacity
Soil physicochemical properties are reported, but biological degradation capacity is often unknown	Add soil biological capacity measurements	Report microbial biomass or respiration, baseline enzyme activity, and, where possible, PHA-degrader abundance or phaZ/depolymerase indicators	Distinguishes polymer limitation from soil biological limitation; slow degradation may reflect a lack of active degraders rather than poor polymer biodegradability
Mulch films are often tested as pristine samples, although field films are weathered before incorporation	Test pristine and pre-weathered materials	Expose films to UV/heat/mechanical ageing before soil burial and compare with unweathered controls	Determines whether field ageing accelerates or suppresses subsequent soil biodegradation
CO₂ evolution measures mineralization but not residual fragments	Pair CO₂ evolution with residue and fragment tracking	Recover residues at intervals; quantify mass, particle-size distribution, surface chemistry, and residual polyester using appropriate chemical analysis	Distinguishes complete mineralization from fragmentation, delayed degradation, or persistence of polymer residues
A single final biodegradation value or a short 60–90 day window hides degradation dynamics	Require longer time-course sampling	Measure degradation at multiple time points beyond early screening windows rather than only at test termination	Identifies lag phases, rapid early loss, plateaus, delayed degradation, and whether degradation continues or stalls over field-relevant timescales
Results often report total degradation without spatial context	Include placement-specific testing	Compare surface exposure, shallow burial, deeper burial, and mixed-soil incorporation, where relevant to the intended use	Clarifies whether degradation depends on oxygen, light exposure, root proximity, or burial depth
Plant-facing risk is not captured by mineralization alone	Add soil–plant compatibility endpoints for agricultural materials	In parallel tests, measure mineral N/P, germination, early biomass, or crop-relevant phytotoxicity after degradation	Determines whether biodegradation is agronomically compatible, not just chemically complete

These modules would supplement ASTM D5988-18, ISO 17556, and EN 17033 by connecting mineralization data with field temperature and moisture availability, soil biological capacity, material ageing, residue persistence, burial context, and plant-facing outcomes.

**Table 4:** International standards used to assess biodegradation, disintegration, and environmental interaction of polymers (including PHAs) across different test environments.

Primary research question	What must be measured to answer it	Appropriate standard(s)	Scientific basis for standard selection	Additional tests often required
Does this polymer (e.g., PHA) ultimately biodegrade in soil?	Polymer-derived carbon mineralized to CO₂; oxygen demand normalized to theoretical carbon	ISO 17556 or ASTM D5988	Aerobic soil respirometry quantifies ultimate biodegradation independent of fragmentation; microbial assimilation inferred from CO₂ evolution	Residual mass balance; surface morphology (SEM); carbon closure checks
How fast does biodegradation occur in soil?	Time-resolved CO₂ evolution; mineralization rate constants (k, lag time)	ISO 17556 or ASTM D5988 (time-resolved data)	CO₂ accumulation curves capture lag phase, active phase, and plateau behavior typical of microbial polymer utilization	None required for rate alone; residue tracking optional to decouple erosion from mineralization
Is the polymer suitable for agricultural mulch film use?	Soil biodegradation + disintegration + soil safety	ISO 23517 or EN 17033	Standards integrate biodegradation thresholds with soil ecotoxicity and product composition limits; Required or expected for market access	Field exposure trials to capture climate and management effects
Does the material physically disappear or fragment in soil?	Mass loss; disintegration; residue size >2 mm or visual persistence	ISO 23517 (disintegration criteria)	Physical breakdown is assessed separately from mineralization; disappearance ≠ mineralization, carbon conversion	ISO 17556/ASTM D5988 if mineralization is also required
Does biodegradation generate adverse effects for crops?	Seed emergence, biomass, growth inhibition metrics	ISO 11269-1/ISO 11269-2 or OECD 208	Plant assays detect soil toxicity from residues, additives, or degradation products	ISO 17556/ASTM D5988 to relate effects to polymer mineralization
Does the polymer affect soil microbial function?	Changes in C or N transformation rates	OECD 216/OECD 217	Changes in C or N transformation rates	ISO 17556/ASTM D5988 to link function to polymer mineralization
Is the polymer compostable rather than soil-biodegradable?	CO₂ evolution under thermophilic composting	ISO 14855 or ASTM D5338	Elevated temperature and moisture accelerate microbial metabolism; relevant only to composting infrastructure	Soil testing is required if agricultural soil use is claimed
Can the polymer degrade under anaerobic conditions (e.g., digestion or landfill)?	Biogas yield (CH₄ + CO₂) relative to theoretical conversion	ASTM D5511 or ISO 15985	Methanogenic conversion indicates anaerobic biodegradation potential	Aerobic soil testing (ISO 17556/ASTM D5988) if land application is expected
Is the polymer biodegradable in marine environments?	CO₂ evolution in seawater or sediment matrix	ASTM D6691, ASTM D7991, ISO 18830, ISO 19679	Marine standards reflect lower microbial density and slower kinetics than soil	Not transferable to terrestrial claims
Can I claim “biodegradable in agricultural fields”?	No single standard can answer this directly	Combination required: ISO 17556/ASTM D5988 + ISO 23517 + field burial studies	Standards quantify components of performance but do not replicate real fields	Multi-season field burial with residue analysis
Does the polymer leave microplastic residues in soil?	Fragment size distribution; persistence over time	No current ASTM/ISO standard	Microplastic fate remains analytical, not regulatorily standardized	Custom microscopy, FTIR/Raman mapping
Which standard should be cited in a regulatory dossier for soil biodegradation?	Ultimate biodegradation metric	ISO 17556 or ASTM D5988	Widely accepted by regulators	ISO 23517 if agricultural product

ISO, International Organization for Standardization; ASTM, ASTM International; EN, European Norm; OEC, Organization for Economic Co-operation and Development.

## 6 Biodegradation of PHA studied under long-duration field conditions

**Figure 5:**
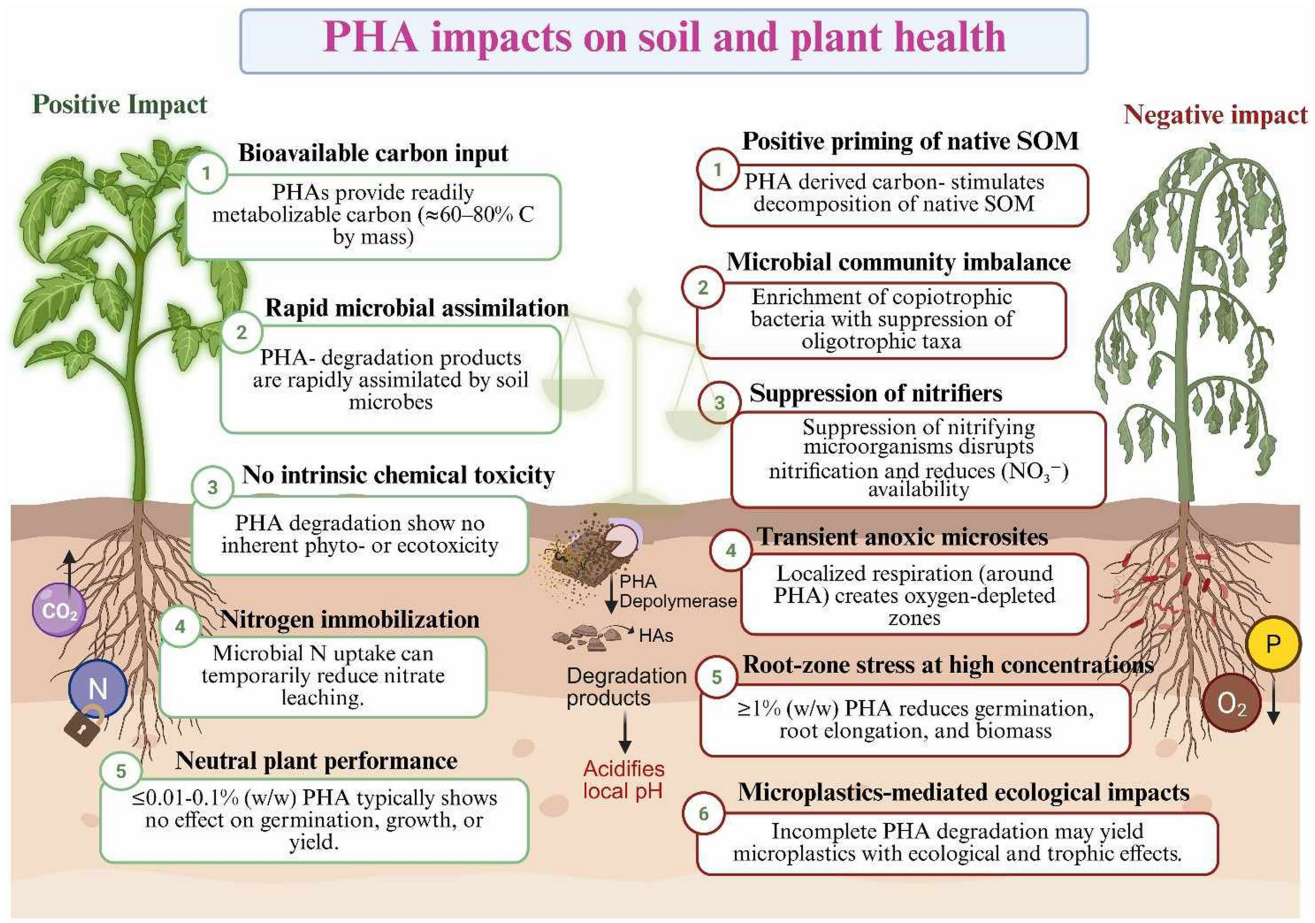
Beneficial and potential adverse effects of PHA inputs in soil systems. At low levels, PHA enhances microbial activity with minimal plant impact, while high concentrations may disrupt microbial processes, stress plants, and contribute to microplastic formation.

Most of what we know about PHA degradation in soil comes from laboratory incubations or field-simulated test beds in greenhouse systems, the carefully controlled systems that are useful but that inevitably smooth out the complexity of real agricultural fields. For example, short-term burial experiments clearly show that PHB is highly susceptible to rapid early degradation in active soils; Chathalingath et al. (2023) observed ~73% mass loss within 3 weeks. Yet such studies capture only the initial phase of polymer–soil interaction and cannot predict longer-term behavior under managed agricultural field conditions. Surprisingly, only a handful of studies have tracked PHA materials under true field exposure, where weather, soil structure, cropping practices, and management disturbance all act simultaneously (Table 5). What these limited fields studies show is, however, important. First, long-term soil burials demonstrate that PHA does biodegrade in agricultural soils, but on substantially longer timescales than those inferred from laboratory microcosms, often extending well beyond a single growing season (Tokiwa and Calabia, 2004). Second, under real field conditions, reported effects of PHA degradation on soil properties, microbial communities, or plant health are typically weak or inconsistent (Figure 5; Lim et al., 2005). Even when changes are observed, they are frequently attributable to dominant environmental or management factors rather than to PHA are frequently attributable to dominant environmental or management factors rather than to PHA degradation itself. Third, unlike laboratory systems, field soils contain multiple carbon inputs from plant residues, root exudates, and organic amendments, which dilute the relative contribution of PHA-derived carbon (Sintim et al., 2020).

**Table 5:** Long-term field and *in situ* studies of polyhydroxyalkanoate (PHA) materials biodegradation in soil environments.

Study reference	*In situ*/*Ex situ* site	Crop/duration	PHA type used: blend/amendment	PHA geometry/features	Temperature (T); pH; moisture % (RH); organic matter (OM%); precipitation (cm)	Observed PHA degradation	Any observed effect on plant health	Any observed nutrient cycling effects	Observed microbial effects
Sintim et al. (2019b)	*In situ*, buried 10 cm deep in soil	Two climates: Tennessee (TN, humid subtropical; Sandy loam) and Washington (WA, cool Mediterranean; silt loam). One full growing season per crop per year; 2015–2016 (2 years); pie pumpkin	PLA/PHA blend mulch film with fillers and additives: 56% PLA, 24% amorphous PHA, 15% CaCO₃, plasticizers/processing aids, 5% carbon black masterbatch (PLA-based).	Films: 33 um thick; 10 × 12 cm; 25gm^−2^; Carbon % 47.5; Biobased content 86%	Y: ~4–30 °C in TN, and 2–25°C at WA; pH: 6.3 ± 0.5 at TN and 6.24 ± 0.11 at WA; OM: 1.43 ± 0.2 at TN and 2.36 ± 0.12; Precipitation: 1313–1,374 mm	Not assessed	Field-scale soil effects of PLA/PHA mulches were minor and transient.	Not assessed	Not assessed
Sintim et al. (2020)	*In situ* surface deployment during crop production, followed by in situ soil burial (10 cm depth of field-weathered mulch material)	Two climates: Tennessee (humid subtropical; Sandy loam) and Washington (cool Mediterranean; silt loam). One full growing season per crop per year; 2015–2018; vegetable crops	PLA/PHA blend mulch film with fillers and additives: 56% PLA, 24% amorphous PHA, 15% CaCO₃, plasticizers/processing aids, 5% carbon black masterbatch (PLA-based).	Films: 33 um thick; 10 × 12 cm; 25gm^−2^; Carbon % 47.5; Biobased content 86%	T: ambient, site- and season-dependent; pH: Not reported; RH: rainfed + managed irrigation; OM: Not reported, Precipitation: natural regional rainfall regimes	0.247 ± 0.009 vs. 0.069 ± 0.012 initial degradation (Knoxville vs. Mount Vernon; ~3.5 × difference); Mw decreased; residue at 600 °C increased to 35 and 28%, respectively.	Not assessed	Not assessed	Not assessed
Bandopadhyay et al. (2020b)	*In situ* surface deployment during crop production, followed by *in situ* soil burial (10 cm depth of field-weathered mulch material)	Two climates: Tennessee (humid subtropical; Sandy loam) and Washington (cool Mediterranean; silt loam). 2 years (2015–16) field trial with vegetable crops.	PLA/PHA blend mulch film with fillers and additives: 56% PLA, 24% amorphous PHA, 15% CaCO₃, plasticizers/processing aids, 5% carbon black masterbatch (PLA-based);	Films: 33 um thick; 10 × 12 cm; 25gm^−2^; Carbon % 47.5; Biobased content 86%	T: ambient, site- and season-dependent; pH: Not reported; RH: rainfed + managed irrigation; OM: Not reported, Precipitation: natural regional rainfall regimes	Not a degradation-rate paper (does not quantify PHA mineralization or mass-loss kinetics as primary outcome; the endpoint is soil microbial response to mulch incorporation). (PMC)	Not assessed	Not assessed	10^9^ g^−1^ dry soil (16S qPCR); abundances increased over time during field exposure and post-season soil burial, but no PLA/PHA-specific enrichment relative to PE or no-mulch controls.
Prudnikova et al. (2024)	*In situ* soil burial (5–10 cm depth)	Multi-latitude field soils (Evenkia, Siberia, Russia: boreal heavy loam soil vs. Sochi region, Kerela, India: subtropical, red ferralitic soil type); 5 months in India and 3 months in Russia.	P3HB solvent cast films	Not mentioned	Siberia: cool summers ~10–20 °C, permafrost soil, pH 7.2, OM 10.7, low humidity. India: subtropical, warm ~20–27 °C, pH 7.2, OM 3.5, high humidity.	Latitude effect: In warm subtropical soil, P3HB degraded ~2 × faster than in Siberian permafrost soil (exact rates not in abstract). Reported 50% mass loss range ~68 days (warm) up to 270 days (cold) for P3HB	Not assessed	Not assessed	Distinct microbiomes drove degradation: Streptomyces and Pseudomonas spp. prevalent in warmer soils vs. more limited actinomycetes in cold soil. Indicates that temperature and native community structure strongly influence PHA depolymerization
Chathalingath et al. (2023)	*In situ* soil burial (10 cm depth)	Fertile garden soil. 3 weeks’ deployment.	P3HB solvent cast films	Solvent cast films; 2 cm × 1.3 cm in diameter with 1 mm thickness.	soil pH 7.45.	73 ± 4.3 reported degradation %. Physical alterations, such as the gradual loss of its parts and color changes, are visible.	Not assessed	Not assessed	Not assessed
Lim et al. (2005)	*In situ*; Buried 2 cm deep in soil	Tropical forest soil (acidic), forest soil near stream (alkaline), and mangrove soil; 112 days.	mcl-PHA films; Control: scl-P3HB films; No polymer blending or additives reported.	Films prepared by solvent casting; Thickness, crystallinity, and molecular architecture not reported	T: 14–27 °C; pH 4.2–8.2; RH: 72–78%; OM: 7–40%	16.7% mass loss (acidic forest soil); 3.0% (alkaline forest soil near stream); 4.5% (mangrove soil); slight Mw decrease only in acidic forest soil; no significant change in Tg, Tm, monomer composition, or polymer structure.	Not assessed	Not assessed	Indirect only; SEM showed surface pits and holes consistent with microbial and detritivore attack; no microbial assays were reported.
Sridewi et al. (2006)	*In situ*; Surface-placed vs. buried at 20 cm depth in sediment	Tropical mangrove ecosystem (intertidal sediment) in Malaysia; 8-week deployment	P(3HB); P(3HB/HV); P(3HB-co-3HHx); P(3HB) + 38 wt% TiO₂ (inorganic-filled composite)	Films prepared by solvent casting; Thickness, crystallinity, and molecular architecture not reported	T: 32 °C; pH 6.0; Permanently water-saturated mangrove sediment; tidal influence at surface	Buried films: ~90–100% mass loss for P3HB, P(3HB/HV); P(3HB-co-3HHx); ~80% mass loss for P3HB + TiO₂ Surface films: slower degradation than buried films Early rapid Mw decrease followed by stabilization; degradation dominated by surface erosion	Not assessed	Not assessed	Microbial CFU counts were not accounted for in burial trials; degradation differences were not explained by bulk microbial abundance
Boyandin et al. (2013)	*In situ*; Buried 15 cm deep in soil	Vietnamese tropical soil; ~12 months deployment.	Melt Pressed; P3HB, and P3HB-HV Films; No blends or additives reported.	Films (30 mm × 0.1 mm; ~73 mg) prepared by solvent casting; pellets (10 mm × 2.5 mm; ~350 mg) prepared by cold compaction of powdered polymer.	T: 26–31 °C; RH: 70–84%; Precipitation: 7–326 cm	P3HB > P3HB/P(3HB/HV); films > pellets; Degradation %: Films: ~98% mass loss; pellets: 8–55% mass loss; Mw decreased by ~15–60% in films and ~60–75% in pellets; crystallinity remained within ~51–71% (change ≤ + 3–10%)	Not assessed	Not assessed	Bacterial counts on PHA surfaces reached ~10^8^–10^9^ CFU g^−1^, exceeding bulk soil levels (~10^6^–10^7^ CFU g^−1^); Isolation yielded 9–48 PHA-degrading bacteria, 4–12 actinomycetes, and 14–48 fungi from PHA specimen surfaces

Only studies involving outdoor deployment or soil burial under natural environmental conditions are included; results from controlled laboratory incubations are considered field-relevant but not equivalent to open-field trials. Here: CFU: Colony-forming unit(s)PLA, Polylactic acid: PHA, Polyhydroxyalkanoate(s); P(3HB), Poly(3-hydroxybutyrate) homopolymer; P(3HB/HV), Poly(3-hydroxybutyrate-co-3-hydroxyvalerate) copolymer; HHx, 3-hydroxyhexanoate; P(3HB-co-3HHx): Poly(3-hydroxybutyrate-co-3-hydroxyhexanoate) copolymer; scl-PHA: Short-chain-length polyhydroxyalkanoates (≤ C5 monomers); mcl-PHA: Medium-chain-length polyhydroxyalkanoates (C6-C14 monomers): OM: Organic matter; TiO₂: Titanium dioxide.

The multiyear field experiments reported by Sintim et al. (2020) represent one of the few long-term studies evaluating PHA behavior under realistic agricultural conditions (Table 5). PHA-based mulch films were tracked across their full lifecycle from surface application during crop production to subsequent soil incorporation over multiple crops, growing seasons (2015–2018), and contrasting field sites (humid subtropical sandy loam in Knoxville, Tennessee, and cool maritime silt loam in Mount Vernon, Washington). After initial field use, weathered mulch samples were buried (~10 cm depth) and monitored over 3 years, with retrieval at 6-month intervals, enabling assessment of multi-seasonal degradation under active agricultural disturbance (tillage, irrigation, and climate variability). Results demonstrated that moisture acted as a primary gatekeeper of degradation, with mulch films degrading approximately twofold faster in the warmer, wetter Tennessee soils (61–83% weight loss) compared to the cooler, drier Washington soils (26–63% after 18 months). Thermogravimetric analysis further confirmed degradation, showing an increase in inorganic residue (~35% in Knoxville and ~28% in Mount Vernon), consistent with progressive loss of organic polymer components during soil exposure. This climate-driven variability is further supported by multi-latitude field studies, where P3HB degradation occurred approximately twofold faster in warm subtropical soils compared to cold boreal soils, with time to ~50% mass loss ranging from ~68 days in warm conditions to ~270 days in colder environments, highlighting the strong influence of temperature and native microbial communities on degradation behavior (Prudnikova et al., 2024; Sintim et al., 2020). For farmers, these studies have a related implication. It means PHA mulch or fertilizer coatings will break down more quickly in rainy season crops or under irrigation, whereas mulch films might persist longer through a drought or winter, potentially impacting the soil the next season. Furthermore, in one of the ‘Sintim groups’, prior studies published in 2019 (Sintim et al., 2019a), the group had reported that PLA/PHA mulches did not produce consistent or sustained changes in bulk soil properties across multiple seasons. Occasional site and season-specific responses were observed, including transient changes in soil hydraulic indicators, SOM, CO_2_-C, and extracellular enzyme activity ratios (C: N and C: P), which showed non-persistent effects over time. The authors note that in open field conditions, where the environment is not controlled, routine management processes, such as tillage, crop growth, rainfall, and seasonal temperature variation, repeatedly re-establish soil conditions (Sintim et al., 2019a). Therefore, in contrast to laboratory conditions where results are controlled and reproducible, open field environments are subject to continuous disturbance and variability. As a result, localized or short-term effects associated with PLA/PHA degradation are often not consistently detectable in field settings (Sintim et al., 2019a, 2019b, 2020).

Another study worth noting is by Bandopadhyay et al. (2020b), which was also conducted as a long-term field experiment in a setting comparable to that of Sintim et al. However, rather than focusing on polymer fate, this study examined soil microbial community structure, abundance, and functional responses following repeated incorporation of PHA-containing mulches. High-resolution 16S rRNA, qPCR, and enzyme assays by Bandopadhyay et al. (2020b) showed that soil microbial dynamics are governed primarily by environmental and seasonal factors rather than mulch composition. Bacterial abundance (~10^8^ gene copies g^−1^ soil) increased across seasons, with enzyme activities (C, N, P cycling) varying strongly with site conditions, tillage, temperature, and moisture. At the Washington site, several enzymes fluctuated seasonally, while cellobiosidase (≈10–22 nmol g^−1^ h^−1^) and leucine aminopeptidase (≈200–375 nmol g^−1^ h^−1^) remained stable. At the Tennessee site, reductions in enzymes such as N-acetyl-glucosaminidase across all treatments (including polyethylene) indicate that microclimate effects, rather than polymer inputs, dominate microbial responses. Although not conducted in managed agricultural fields, the study by Lim et al. (2005) conducted an *in situ* soil burial study comparing mcl- and scl-PHA films across three tropical environments (forest, stream-adjacent, and mangrove soils). Results showed that mcl-PHAs degraded slowly and in a site-dependent manner, with <20% mass loss and minimal structural changes, whereas scl-PHAs exhibited substantial degradation (~73.5% weight loss over 112 days). Similarly, studies in tropical mangrove sediments have shown that burial conditions strongly influence degradation, with buried PHA films exhibiting rapid mass loss (~90–100% within 8 weeks), while surface-exposed films degraded more slowly, indicating that moisture saturation and sediment conditions accelerate degradation processes (Sridewi et al., 2006). These studies are therefore valuable as a conservative, field-relevant benchmark for PHA persistence outside controlled laboratory systems. Boyandin et al. (2013) evaluated long-term *in situ* degradation of P3HB and P(3HB/HV) in tropical forest and mangrove soils under natural conditions. Burial at 15 cm depth showed that degradation proceeds over months, even in warm, moist environments. After ~12 months, P3HB films exhibited 47–98% mass loss, while P(3HB/HV) degraded more slowly and variably (14–61%). Pellets degraded at lower rates (28–55% for P3HB and 8–35% for P(3HB/HV)), with overall rates of ~0.04–0.33% day^−1^ for films and ~0.02–0.18% day^−1^ for pellets. Molecular weight reduction (from ~1,400 kDa to ~360–1,160 kDa after 180 days) with minimal changes in crystallinity indicates preferential erosion of amorphous regions. Microbial abundance on the plastisphere was estimated at ~10^8^–10^9^ CFU g^−1^ soil, confirming active microbial involvement in degradation. Together, all these data establish long-term soil exposure and impart caution. Biodegradation is highly environment specific. A biodegradable plastic that vanishes in one field may persist in another.

## 7 Microbial and soil biological responses to PHA degradation

Soil provides a plethora of agroecosystem functions and services, including carbon (C) sequestration and the provision of food resources (Lal, 2011). Consequently, understanding how PHA biodegradation interacts with soil microbial communities and soil carbon dynamics is critical for evaluating the environmental suitability of PHA-based agricultural materials. One of the most attractive properties of PHA concerning ecology is that PHAs represent an abundant source of concentrated, soluble, and highly metabolizable organic carbon, with carbon contents typically in the range of 60–80% by mass (Szacherska et al., 2021). Following enzymatic depolymerization, PHAs yield low molecular weight intermediates dominated by 3-hydroxybutyric acid, and water-soluble oligomers typically contain fewer than 10 monomer units (Rychter et al., 2006). Quantitative evidence indicates that these.

degradation products are readily assimilated and mineralized by soil microorganisms rather than persisting as chemically inert residues, distinguishing PHA-derived carbon fundamentally from petroleum-based microplastics. When introduced into soil, PHAs therefore function not merely as physical residues, but as metabolizable carbon substrates capable of entering active microbial metabolic pathways, and directly interacting with soil microbial communities, once depolymerization is initiated (Zhou et al., 2023). Previously, neither PHA production nor degradation was reported with intrinsic chemical toxicity (Dhaini et al., 2024). However, biodegradability alone does not guarantee ecological neutrality. Instead, the ecological consequences of PHA degradation depend on how soil microorganisms process polymer-derived carbon, and how the end products feed back into soil function. Within this context, understanding the underlying microbial mechanisms becomes critical, as PHA-related processes in the rhizosphere operate through distinct but interconnected pathways.

Experimental evidence indicates that PHA-related processes in the rhizosphere are governed by two related and distinct mechanisms. The first mechanism is intracellular PHB turnover in plant-associated bacteria, where PHB functions as a dynamic reserve of carbon and reducing power that is accumulated during early colonization and later mobilized to contribute to bacterial fitness and plant growth-promoting capacity under rhizosphere-associated stress conditions. In *Herbaspirillum seropedicae*, genetic disruption of PHB synthesis or depolymerization impaired the bacterium’s capacity to promote plant growth, while root-based reporter assays showed early activation of PHB biosynthetic genes followed by later induction of depolymerase expression, consistent with a storage-then-remobilization strategy during root association (Alves et al., 2019). Closely related findings in *Azospirillum brasilense* further show that PHB mobilization enhances starvation tolerance, oxidative and osmotic stress resistance, and persistence in the rhizosphere, with PHB-metabolism genes expressed during wheat root association, supporting the view that reserve polymer turnover underpins microbial fitness during plant interaction (Kadouri et al., 2003; Edelshtein et al., 2003). By contrast, the second mechanism, which is more directly relevant to exogenous PHA materials added to planted soils, is microbial use of the polymer as a localized carbon source. Soil studies show that PHB and PHBV amendments create carbon-rich microsites that enrich PHA-degrading microorganisms, increase depolymerase-linked markers, stimulate respiration, and elevate enzymes involved in carbon and nutrient acquisition (Brtnicky et al., 2022, 2024). The inhibition of plant growth under such conditions appears to arise from the nutrient imbalance created by this carbon input. Because PHAs contain carbon but no nitrogen, phosphorus, or sulfur, microbial growth on PHA-derived carbon can increase demand for soil nutrients and promote their immobilization in microbial biomass. Under high or localized PHA loadings, this can reduce nutrient availability to plants and intensify plant–microbe competition (Brtnicky et al., 2025a). This mechanism provides a stronger explanation for the growth inhibition reported in pot studies than direct polymer toxicity, for which evidence remains limited. However, these studies largely measure bulk soil responses. Direct rhizosphere scale evidence remains limited, and it is still unclear whether root exudates, root-zone oxygen gradients, or plant signaling directly regulate extracellular PHA depolymerization. Thus, the current evidence supports a microbial carbon demand mechanism in soil, but the specific rhizosphere controls remain unresolved.

### 7.1 Stimulation of soil metabolic activities through formation and utilization of PHA degradation products

Across independent laboratory and field-oriented studies, a consistent enzymatic signature emerges in soils exposed to PHBV and related PHAs, with carbon-acquiring and nitrogen-recycling enzyme responses being among the most reproducible outcomes. For instance, prior published works have reported a substantial increase in *β*-glucosidase, *α*-glucosidase, β-xylosidase, and cellobiosidase activity following PHA amendment in soil, with activities ranging from approximately twofold to an order of magnitude, particularly within polymer-associated microsites relative to surrounding bulk soil (Bandopadhyay et al., 2020b; Brtnicky et al., 2022; Zhou et al., 2021; Withana et al., 2025). This pattern points toward accelerated decomposition of native soil polysaccharides, including cellulose, and is widely interpreted as evidence of positive priming by PHAs, whereby microbes maintain higher access to existing carbon pools to sustain elevated metabolism. In this context, PHA-derived carbon, seemingly, does not substitute itself for SOM utilization but instead simulates broader microbial metabolism, increasing access to pre-existing carbon pools. With higher PHA loadings, this enzymatic response can, however, extend to oxidative and hydrolytic enzymes implicated in the subsequent breakdown of more recalcitrant SOM fractions themselves. Earlier experimental studies by Brtnicky et al. (2022, 2025a) reported that dehydrogenase activity increases in bulk soil during PHA degradation. In contrast, a more recent review by Brtnicky et al. (2025b) highlights elevated levels of N-acetyl-β-D-glucosaminidase (NAG) in soils exposed to PHBV. In plant metabolism, NAG is a key enzyme involved in defense responses against pathogens and is integral to the degradation of chitin, both externally in the soil and internally within the plant (Shobade et al., 2024). However, dehydrogenases are intracellular enzymes associated with microbial respiration; elevated DHA is commonly interpreted as an indicator of increased overall microbial oxidative metabolism rather than a specific nutrient transformation (Velmourougane et al., 2013). Collectively, these studies indicate that PHA inputs into soil systems stimulate rapid microbial degradation of organic matter, resulting in accelerated turnover and transient release of labile, free-form carbon in soil.

This mobilization of extra carbon in soil transiently increases the levels of biologically assimilated carbon in soil through the biomass numbers, but only to be released back to the atmosphere (Sintim et al., 2019a). Incubation studies using agricultural soils report that approximately 80–95% of PHA carbon is respired as CO_2_ (Ding et al., 2021). This portioning is consistent with results from isotope tracing studies. Experiments employing ^13^C-labeled biodegradable polymers have demonstrated that biopolymer-derived carbon is incorporated into microbial biomass (including bacterial and filamentous fungal lipids and amino acids) only transiently, and subsequently mineralized to CO_2_, confirming direct microbial assimilation rather than purely abiotic loss (Mason-Jones et al., 2019; Zumstein et al., 2018). Although this study focused on PBAT, the metabolic logic applies directly to PHAs, which are structurally simpler and biochemically closer to intracellular microbial storage polymers. This rapid bioavailability distinguishes PHAs from both fossil plastics and more complex plant-derived polymers such as lignin. Crucially, there is still a lack of any published or laboratory study supporting the assumption that PHA inputs contribute meaningfully to carbon sequestration over longer periods. No current evidence conclusively demonstrates persistent retention of PHA-derived carbon in stable soil carbon pools, whether mineral-associated organic matter or stable soil aggregates, over seasonal or multi-year timescales. This, therefore, remains a key open question that warrants targeted isotope-enabled field studies, particularly in agricultural systems where PHAs are increasingly proposed as mulch films, coatings, or soil contact materials.

Relative to carbon cycling enzymes, far fewer studies have examined nitrogen, phosphorus, or sulfur cycling following PHA or PHBV inputs, despite their central importance for plant nutrition. Where measured, PHA amendments consistently increase nitrogen-acquiring enzymes such as urease (Brtnicky et al., 2022, 2025a) NAG (Brtnicky et al., 2025b), and leucine aminopeptidase (Zhou et al., 2021), indicating elevated microbial demand for organic nitrogen. In contrast, phosphatase and arylsulfatase responses are weak, variable, or absent. This pattern has been observed in both bulk-soil incubations and PHA-enriched microsites, particularly under higher polymer loadings. In these systems, elevated NAG activity may reflect intensified turnover of microbial necromass and fungal-derived substrates, while increased leucine aminopeptidase activity may indicate enhanced depolymerization of soil proteins to satisfy microbial nitrogen demand. Importantly, these enzymatic responses occur despite the absence of nitrogen in the PHA backbone itself, demonstrating that PHA inputs indirectly signal the onset of microbial nitrogen limitation and stimulate microbial nitrogen mining from existing SOM. Across studies that have explicitly measured enzyme responses to PHA or PHBV inputs, PHA-specific depolymerases (PhaZ) are consistently reported to be elevated relative to unamended controls, particularly at high polymer loading (Brtnicky et al., 2025b). However, none of these studies demonstrate a direct mechanistic linkage whereby PhaZ activity itself drives broader enzyme induction.

Although not focused on PHA, microcosm studies on hydrolysable biodegradable microplastics provide mechanistic insight relevant to PHA behavior in soil. These studies show that polymers containing ester bonds can serve as localized carbon substrates for soil microorganisms, forming polymer-specific “plastisphere” habitats (Schöpfer et al., 2022). While bulk soil microbial abundance, respiration, and extracellular enzyme activities may remain unchanged, enhanced activities of depolymerizing enzymes (e.g., lipases, esterases, and *β*-glucosidases) are detected directly on polymer surfaces, a conclusion further supported by Zhou et al. (2021) who showed enzyme enrichment and intensified microbial activity within PHBV plastispheres. This is because polymer degradation occurs in small, localized hotspots that strongly affect nearby microbes and roots but are masked when soil properties are measured at the bulk scale. Importantly, these findings also suggest that biodegradation may involve transient intermediate stages, including oligomers, low-molecular-weight hydrolysis products, and partially degraded crystalline-rich fragments, that remain poorly characterized in most PHA soil studies, which primarily report bulk endpoints such as mass loss or CO_2_ evolution. Resolving the formation, persistence, and microbial fate of these intermediates is therefore critical for understanding localized carbon release, transient fragment residence, and short-term soil biogeochemical responses. Future studies should therefore prioritize plastisphere-scale processes using spatially resolved methods, such as soil zymography to visualize enzyme activity directly at polymer surfaces, microsensor-based measurements to capture localized respiration and redox conditions, and stable isotope probing (e.g., 13C-labeled polymers) to make these hidden processes visible and explain how localized carbon release can still drive measurable changes in soil function and plant performance.

Nevertheless, despite growing interest in PHA soil interactions, the empirical foundation linking PHAs to soil enzymatic function remains strikingly thin. As summarized in Table 6, only a handful of studies have directly quantified soil enzyme responses to PHAs while concurrently assessing microbial dynamics or plant-level outcomes. Critically, this evidence base is almost entirely restricted to short pot and microcosm experiments using PHB or PHBV formulations, with no field-scale studies that resolve enzymatic responses under agronomically realistic conditions. As a result, a significant research gap exists in understanding how PHA-induced enzymatic shifts propagate through nutrient cycling to influence crop performance, which is largely fragmented, highly context-dependent, and difficult to extrapolate beyond controlled systems.

**Table 6:** Current evidence linking PHA inputs to soil enzyme activity and biological responses in agricultural systems.

PHA material and its physical form used in soil	System/temperature (°C)/relative humidity (%)/light intensity and photoperiod	Soil/crop/zone of study	Enzymes measured	Direction reported (PHA vs. control)	Probable soil biogeochemical implications inferred from enzyme responses.	Inferred soil–microbe–plant implications	Reference
1 wt% P3HB microparticles/powder (<63 μm) mixed into soil/sand gradients.	Pot experiment; 18–20 °C; ~70% RH; ~20,000 lx; 8 weeks	Haplic Luvisol silty clay loam mixed with quartz sand/ lettuce/bulk soil	DHA, ARA, NAG; Urea, GLU; Phos	DHA ↑; Ure ↑; GLU ↓; ARS, NAG, Phos variable	Strong stimulation of microbial respiration and urea turnover indicates rapid microbial growth and nitrogen immobilization; reduced β-glucosidase suggests decoupling of native SOM cellulose breakdown from N release, leading to nutrient imbalance in the root zone	Suppressed lettuce growth linked to reduced plant-available N and P; P3HB degradation not quantified	Brtnicky et al. (2022)
P3HB microparticles/powder (<63 μm) alone+ compost+P3HB; digestate+P3HB	Pot experiment; 18–20 °C; ~70% RH; ~20,000 lx; 8 weeks	Haplic Luvisol + quartz sand; lettuce; bulk soil	GLU, NAG, Phos, ARS, Urease	P3HB alone: DHA ↑, Ure ↑; Compost+P3HB: broad enzyme stimulation except GLU ↓; Digestate+P3HB: DHA ↑, ARS ↑, NAG ↑, Ure ↑ (respiration ↑)	P3HB alone promotes microbial N immobilization; compost accelerates depolymerization but does not restore nutrient balance; digestate supplies mineral N and labile substrates, partially re-coupling C-N cycling and moderating microbial competition	P3HB alone reduced lettuce biomass; compost was ineffective; digestate mitigated growth suppression and accelerated degradation	Brtnicky et al. (2025a)
P3HB microplastics (<80 μm) at 0–10% (w/w)	Pot experiment; 12–20 °C; 45–70% RH; ~20,000 lx; 90 days	Haplic Luvisol; maize (*Zea mays*); bulk soil	NAG and PhaZ	NAG ↑; phaZ ↑ at higher loadings	Elevated phaZ confirms active biological depolymerization; increased NAG reflects intensified microbial necromass turnover and strong microbial N demand under carbon-rich conditions.	Focused on microbial community shifts and N pools; enzyme-plant linkage not directly tested	Brtnicky et al. (2025b)
P(3HB/HV) microplastics (10% w/w)	Soil microcosm/hotspot pot experiment; 14–24 °C; ~40% RH; ~800 μmol m^−2^ s^−1^; 25 days	Topsoil; wheat (*Triticum aestivum*); plastisphere hotspot	β-glucosidase, acid phosphatase, leucine aminopeptidase	Enzyme activities ↑ (up to ~2×) in plastisphere hotspots	Localized stimulation of C, N, and P acquisition enzymes indicates strong positive priming of native SOM at polymer–soil interfaces, driving nutrient mobilization toward microbial demand.	Plant performance not directly assessed; study established plastisphere-scale eco-enzymology	Zhou et al. (2021)
Endogenous P3HB synthesis (transgenic) or exogenous 3-HB	Controlled plant study	Flax (*Linum usitatissimum* L.)	β-ketothiolase (gene expression)	Stress- and chromatin-related pathways ↑	Indicates that P3HB -derived metabolites can act as biochemical signals influencing plant epigenetic and stress-response pathways, distinct from soil-mediated effects.	Reduced growth linked to metabolic and signaling changes	Mierziak et al. (2020)

PHA, Polyhydroxyalkanoate(s); P(3HB), Poly(3-hydroxybutyrate) homopolymer; P(3HB/HV), Poly(3-hydroxybutyrate-co-3-hydroxyvalerate) copolymer; RH, Relative Humidity; NAG, N-acetyl-D-glucosaminidase, and PhaZ, PHA depolymerase; DHA, Dehydrogenase; ARS, Arylsulfatase; Ure, Urease, GLU, β-glucosidase, Phos, phosphatase.

### 7.2 Trade-offs in microbial biomass and community structure

Despite increased metabolic activity, microbial biomass responses to PHAs are not uniformly positive. Several studies report that high PHA loadings reduce total microbial biomass, particularly fungal biomass. Notably, Reay et al. (2025) measured microbial phospholipid fatty acid (PLFA) biomass and found it was significantly (*p* = 0.043) lower in all PHBV treatments vs. control, despite evidence of polymer degradation. Gram-positive and Gram-negative bacteria PLFAs declined most strongly at PHBV concentrations between 1.6 and 3.2%, while fungal PLFAs were significantly reduced at concentrations of ≥ 0.6%. Consequently, the fungal: bacterial ratio declined, indicating a shift toward bacterial dominance.

Community composition analysis from prior studies further reveals that PHAs act as strong ecological selectors in soil, preferentially enriching copiotrophic and polymer responding taxa, while suppressing oligotrophic and functionally specialized groups. In agricultural soils amended with PHBV, Reay et al. (2025) themselves documented a 3–15x increase in bacterial lineages associated with rapid utilization of labile carbon, including *Oxalobacteraceae*, *Comamonadaceae, Sphingomonas, Terrabacter*, and multiple *Alphaproteobacteria* and *Gammaproteobacteria* lineages. These groups are well recognized as fast-growing heterotrophs that dominate short-lived carbon pulses in soil and are frequently implicated in the degradation of biodegradable polymers and other readily assimilable substrates in soil (Fierer et al., 2007; Goldfarb et al., 2011). Their dramatic enrichment indicates that PHBV degradation creates localized “hotspots” of elevated carbon availability that strongly favor opportunistic degraders, consistent with their role as classic copiotrophs that respond rapidly to fresh carbon inputs (Kuzyakov and Blagodatskaya, 2015). This reinforces that PHA biodegradation restructures microbial communities primarily through carbon-driven niche selection rather than toxicity or inhibition. Brtnicky et al. (2025b) further demonstrated that increasing P3HB inputs not only enriched aerobic PHA-degrading groups such as *Actinobacteria* and *Alphaproteobacteria* (with families such as *Microbacteriaceae, Caulobacteriaceae, Pseudomonadaceae,* and *Rhizobiaceae*) but also promoted the appearance of anaerobic *Clostridia*, indicating that intense microbial respiration around PHA particles can generate short-lived, spatially restricted anoxic microsites (tens-hundreds of micrometers). This highlights how PHA degradation can restructure redox conditions at the microscale levels. Despite microscale redox conditions, bulk oxygen availability is maintained, especially in agricultural topsoils that are tilled, irrigated, or otherwise aerated. This phenomenon is well documented for other labile carbon hotspots (e.g., root exudates, particulate organic matter, and compost fragments). These localized changes may also influence the chemical form and bioavailability of redox-sensitive elements such as iron and manganese, which act as cofactors in microbial enzymatic processes.

However, these oxygen-depleted microzones within the PHA plastisphere can transiently become unfavorable for aerobic processes such as nitrogen fixation, as supported by previous studies. For instance, in the studies by Reay et al. (2025) and Brtnicky et al. (2025b) the authors have also reported marked declines (≈2–5x) in taxa that dominate stable soil environments, contribute to nitrogen cycling, and maintain activity under low resource and energy-limited conditions, including *Nitrososphaeraceae, Nitrospiria, Vicinamibacterales, Ca. Udaeobacter, Chloroflexi,* and several *Gaiellales* lineages. Some of the fungal families, reported to be suppressed, *Nectriaceae* and *Plectosphaerellaceae* (Brtnicky et al., 2025b). Moreover, their suppression likely reflects resource-driven competitive exclusion, whereby fast-growing heterotrophic microorganisms perhaps monopolize limiting resources, including available nitrogen, phosphorus, and colonizable microsites. Zhou et al. (2021) reported that the dissolved organic nitrogen decreased by 66% in the PHBV-treated soil compared to the control soil. Sintim et al. (2019a) report that when compared to no mulch controls, the nutrient cycling function was decreased by 11% by mulch film composed of PLA/PHA (Brtnicky et al., 2025b). Furthermore, their suppression likely reflects resource-driven competitive exclusion, whereby fast-growing heterotrophic microorganisms perhaps monopolize limiting resources, including available nitrogen, phosphorus, and colonizable microsites. Zhou et al. (2021) reported that the dissolved organic nitrogen decreased by 66% in the PHBV-treated soil compared to the control soil. Sintim et al. (2019a) report that when compared to no mulch controls, the nutrient cycling function was decreased by 11% by mulch film composed of PLA/PHA. This competitive advantage disfavors autotrophic nitrifiers, which exhibit slower growth rates. Accordingly, the pronounced declines observed in ammonia (NH_4_^+^)-oxidizing archaea (*Nitrososphaeraceae)* and nitrite (NO_2_^−^)-oxidizing bacteria (*Nitrospiria*) indicate that PHA degradation can transiently reduce or disrupt total nitrification, a process that is essential for regulating plant available nitrogen and preventing ammonium accumulation in agricultural soils. Ultimately, suppression of nitrification reduces NO_3_^−^ availability for downstream denitrification, thereby lowering the potential for climate-harmful nitrous oxide (N₂O) production. This mechanistic link explains why some studies have reported no increase in N_2_O emissions following PHA applications, even under conditions of elevated heterotrophic respiration and carbon turnover (Greenfield et al., 2022). In addition, there is also reported evidence that PHAs may further constrain nitrogen transformations through physically sorbing inorganic nitrogen species, including NO_3_^−^ (Zhou et al., 2023). Because NO_3_^−^ is highly mobile in soil, lowering its concentration reduces the risk of nitrate leaching beyond the root zone, which is widely recognized as a major environmental challenge in agriculture. In this respect, PHA-derived carbon behaves similarly to other carbon-based soil amendments, such as cover crop residues or organic matter additions, which are commonly used to stimulate nitrate immobilization and reduce nitrate losses during vulnerable periods. This outcome is generally considered beneficial, provided the timing and dose of PHA applications are adjusted, ensuring the nitrogen remains available to crops when demand is high.

Similarly, reductions in *Bacillus* and other stress-tolerant Firmicutes suggest that these taxa adapted to episodic substrate availability. *Vicinamibacterales, Chloroflexi, Gaiellales,* and *Ca. Udaeobacter,* in particular, are among the most ubiquitous bacteria in cropland soils. They are believed to contribute to the decomposition of recalcitrant plant materials (such as cellulose and hemicellulose), turnover of SOM, and nutrient cycling, including trace gas (H₂) oxidation, thereby supporting overall soil health and fertility across wet–dry cycles (Willms et al., 2020; Huber and Overmann, 2018; Speirs et al., 2019). Together, these changes imply that intense or localized PHA degradation can temporarily weaken microbial functions responsible for nitrogen retention, slow organic carbon processing, and system stability, even as overall microbial activity increases. However, these effects are dose-dependent. Across available studies, low and field-relevant PHA inputs, generally estimated at ≤0.01–0.1% w/w soil, are associated with minimal, weak, or non-persistent changes in bulk soil properties, microbial community composition, and crop performance (Zhang et al., 2025; Schöpfer et al., 2022). In contrast, higher experimental loadings, particularly ≥ 1% w/w, consistently induce strong microbial community shifts, enhanced microbial respiration, nutrient immobilization, reduced mineral nitrogen availability, and impaired plant growth (Brtnicky et al., 2025c). At very high loadings (5–10% w/w), several studies report severe growth suppression, indicating that plant stress is most likely when PHA-derived carbon is applied at higher concentrations (Brtnicky et al., 2022, 2025a, 2025b).

This distinction is important because practical agricultural exposure is unlikely to resemble homogenized high-dose pot experiments. Under field conditions, PHA residues from mulch films or soil-contact materials are typically lower in concentration, spatially dispersed, and exposed to variable moisture, temperature, and microbial activity. Collectively, the available evidence suggests that PHA effects on soil health and plant performance are governed less by polymer presence alone than by the dose, spatial distribution, soil nutrient status, and crop context. Concentrated PHA hotspots can trigger short-term nutrient competition and plant stress, whereas realistic field-level inputs are more likely to produce neutral or limited effects. These threshold ranges are summarized in Figure 5.

As for the fungal diversities, in the study by Brtnicky et al. (2025b) increasing P3HB concentrations did not stimulate the soil fungal community broadly but instead selectively enriched a narrow subset of taxa, including *Tetracladium, Exophilia,* and *Pseudogymnoascus*, capable of exploiting polymer-derived carbon (Kim et al., 2000). *Tetracladium* species encode diverse hydrolytic enzymes, including esterases and lipases implicated in the degradation of complex plant and synthetic polyesters (Lazar et al., 2025). Black yeasts of the genus *Exophilia* are well documented with the capacity to utilize recalcitrant and xenobiotic carbon substrates (Tesei, 2022). *Pseudogymnoascus,* a saprotrophic and often psychrotolerant ascomycete, is well adapted to particulate organic matter and has been implicated in the turnover of biodegradable polymer. It is commonly associated with fertile agricultural soils and contributes to polymer decomposition (Parray and Li, 2025). Together, these patterns support a model in which P3HB inputs selectively recruit a functionally coherent fungal consortium that governs early-stage polymer breakdown and shapes downstream microbial and biogeochemical responses.

### 7.3 Bulk soil physical properties and microenvironmental effects of PHAs

Compared with their well-documented microbial and biochemical effects, the influence of PHAs on bulk soil physical properties remains sparsely investigated. Most available evidence indicates that the addition and degradation of PHA do not substantially alter whole soil texture, aggregation, porosity, or bulk density under agricultural conditions. Instead, reported effects are largely confined to localized microenvironments, the ‘plastisphere’ zones surrounding polymer particles, where transient changes in wettability, aeration, and redox conditions may occur. In a controlled pot experiment, Reay et al. (2025) demonstrated that soils amended with high PHBV concentrations (1.6–3.2% w/w) exhibited significantly increased hydrophobicity relative to controls (*p* < 0.001), in the microsites just surrounding the PHBV particles. These localized hydrophobic zones were hypothesized to impede short-range diffusion of water and solutes without affecting the bulk soil moisture balance. Field evidence supports this microscale interpretation. In comparative mulch trials, conventional polyethylene films reduced soil moisture during wet periods in field trials of winter barley, whereas PHBV mulch films caused no measurable change in soil moisture dynamics under agricultural conditions (Greenfield et al., 2022).

Moreover, since PHAs and their degradation products are less dense (~1.2–1.3 g cm^−3^) than mineral soil constituents (typically ~2.6–2.7 g cm^−3^), their presence may slightly alter pore geometry at the microscale to mesoscale (Dominguez-Candela et al., 2024). When PHA particles are incorporated into soil, they can function as low-density inclusions that displace mineral grains. As microbial depolymerization proceeds, the gradual erosion and disappearance of these inclusions can leave behind transient voids or channels, effectively increasing local pore volume. Studies examining biodegradable polymers in agricultural soil have reported reductions in bulk density and shifts toward larger pore fractions (particularly macropores). Yet, it is important to remember that even these effects are spatially heterogeneous and concentrated around polymer-rich microsites rather than expressed uniformly at the field scale (Zhou et al., 2021; Nayab et al., 2022). Furthermore, beyond purely physical displacement, PHAs can strongly engineer soil structure through biological processes. PHA particles rapidly promote the formation of plastisphere biofilms that produce EPS. EPS acts as a biological binding agent, promoting aggregation and reorganizing soil particles into stable micro- and macro-aggregates, which inherently increase inter-aggregate porosity. While direct quantification of long-term increase in stable macroporosity or aggregate stability following PHA complete mineralization remains unproven, it has been reported for other biopolymers and plastic residues. Studies have reported that the introduction of persistent plastic particles can promote undesirable soil aggregation, reducing microbial access to organic substrates, altering nutrient partitioning and slowing their turnover, causing further losses of SOM, and even disrupting soil food web dynamics (Brtnicky et al., 2022).

## 8 Influence on cytotoxicity and plant performance

The interaction between PHAs and plants represents another critical interface for evaluating the agronomic suitability of PHA-based materials. Across laboratory, pot, and limited field studies, a consistent picture emerges that neither PHAs nor their degradation products (possibly present temporarily in the soil) are intrinsically phytotoxic, but degradation can substantially alter soil nutrient dynamics and microbial competition, producing plant responses that are strangely dose and context dependent (Brtnicky et al., 2022). Early ecotoxicological assessments by Rychter et al. (2006) demonstrated that post-degradation soil (undergoing PHB degradation) showed no adverse effects on seed germination, seedling morphology, or plant biomass dry matter yield when tested using standardized terrestrial plant growth assays. Only minor and temporary changes in soil pH or salinity have been reported during PHA degradation, and these changes remained well within agronomically acceptable ranges. Other studies, inputting PHA at agronomically realistic levels, have also consistently shown neutral effects on plant performance. A barley field trial applying approximately 100 kg ha^−1^ PHBV (≈0.01% w/w soil concentration) reported no changes in crop yield, microbial community structure, or nutrient availability (Greenfield et al., 2022). Similarly, Reay et al. (2025) observed no effects on maize growth, tissue nutrient status, or soil microbial biomass at 0.06% PHBV. Dahal et al. (2020) show that biodegradable materials, including PHBV, can be deployed in fields as low-cost, maintenance-free biodegradable agricultural devices (as a soil moisture sensor in this study) without negative impacts on maize growth, under greenhouse conditions. Their results highlight PHBV as a rapidly degrading option (<60 days) for short-lived soil applications, whereas wax blends provide structural stability for long-term functionality. None of these published papers reported any germination failures due to PHAs. Thus, under expected agricultural exposures (mulch residues, compost contamination), PHA addition appears plant neutral Rychter et al. (2006). Other studies, inputting PHA at agronomically realistic levels, have also consistently shown neutral effects on plant performance. A barley field trial applying approximately 100 kg ha^−1^ PHBV (≈0.01% w/w soil concentration) reported no changes in crop yield, microbial community structure, or nutrient availability (Greenfield et al., 2022). Similarly, Reay et al. (2025) observed no effects on maize growth, tissue nutrient status, or soil microbial biomass at 0.06% PHBV. Dahal et al. (2020) show that biodegradable materials, including PHBV, can be deployed in fields as low-cost, maintenance-free biodegradable agricultural devices (as a soil moisture sensor in this study) without negative impacts on maize growth, under greenhouse conditions. Their results highlight PHBV as a rapidly degrading option (<60 days) for short-lived soil applications, whereas wax blends provide structural stability for long-term functionality. None of these published papers reported any germination failures due to PHAs. Thus, under expected agricultural exposures (mulch residues, compost contamination), PHA addition appears plant neutral Rychter et al., 2006). Other studies, inputting PHA at agronomically realistic levels, have also consistently shown neutral effects on plant performance. A barley field trial applying approximately 100 kg ha^−1^ PHBV (≈0.01% w/w soil concentration) reported no changes in crop yield, microbial community structure, or nutrient availability (Greenfield et al., 2022). Similarly, Reay et al. (2025) observed no effects on maize growth, tissue nutrient status, or soil microbial biomass at 0.06% PHBV. Dahal et al. (2020) show that biodegradable materials, including PHBV, can be deployed in fields as low-cost, maintenance-free biodegradable agricultural devices (as a soil moisture sensor in this study) without negative impacts on maize growth, under greenhouse conditions. Their results highlight PHBV as a rapidly degrading option (<60 days) for short-lived soil applications, whereas wax blends provide structural stability for long-term functionality. None of these published papers reported any germination failures due to PHAs. Thus, under expected agricultural exposures (mulch residues, compost contamination), PHA addition appears plant neutral Rychter et al., 2006). Other studies, inputting PHA at agronomically realistic levels, have also consistently shown neutral effects on plant performance. A barley field trial applying approximately 100 kg ha^−1^ PHBV (≈0.01% w/w soil concentration) reported no changes in crop yield, microbial community structure, or nutrient availability (Greenfield et al., 2022). Similarly, Reay et al. (2025) observed no effects on maize growth, tissue nutrient status, or soil microbial biomass at 0.06% PHBV. Dahal et al. (2020) show that biodegradable materials, including PHBV, can be deployed in fields as low-cost, maintenance-free biodegradable agricultural devices (as a soil moisture sensor in this study) without negative impacts on maize growth, under greenhouse conditions. Their results highlight PHBV as a rapidly degrading option (<60 days) for short-lived soil applications, whereas wax blends provide structural stability for long-term functionality. None of these published papers reported any germination failures due to PHAs. Thus, under expected agricultural exposures (mulch residues, compost contamination), PHA addition appears plant neutral.

Comparatively, in some of the recent studies, the use of high PHA concentrations has been shown to suppress plant growth under experimental conditions. For instance, in a series of controlled pot studies, Brtnicky et al., 2025b, 2025c and Reay et al., 2025 reported drastic reductions in maize biomass (post-emergence) at PHBV or P3HB concentrations ≥1% (w/w), with near complete growth failure at 5–10% loading. Nayab et al.’s (2022) study reported an overall reduction by 65% of the Plant Health Index (PHI) of maize plants in soils mixed with PHB microparticles (*<*120 μm diameter) at a homogenous concentration of 5% (w/w). This study also reported significantly lowered (54%) maize seed germination measured by root growth (average root length, root volume, and total root surface area). Malik et al. (2015) further demonstrated that PHB can influence early plant development stages by affecting seed germination, and that the plant responses to PHB are not limited to post emergence growth or soil-mediated effects. Their study reports a statistically significant reduction in germination percentage and a delay in germination kinetics (*p* < 0.05) following PHB or PHB-derived compound exposure. Collectively, this study extends the scope of PHB–plant interactions to the earliest phases of the plant life cycle and underscores the need to consider germination endpoints in future assessments. Other studies have also reported adverse impacts of PHBV, PHB, and P3HB on crop growth (above-ground plant biomass as well as the root biomass) across multiple species, including maize (*Zea mays* L.) (Brown et al., 2023), tomato (*Lycopersicon esculentum* Mill.), and lettuce (*Lactuca sativa* L. var. *capitata*) (Brtnicky et al., 2022; Serrano Ruiz et al., 2022). In one of the most striking observations to date, PHBV amendment led to complete mortality of wheat (*Triticum aestivum* L.) within 25 days of exposure (Zhou et al., 2021). Some authors do note that sensitive plants like Lepidium or Sorghum can be affected much more by PHB/PHBV monomers at high concentrations than agronomic crops (Liwarska-Bizukojc, 2022). Largely, these results are consistent with previous reports indicating that high concentrations of biodegradable polymers, including PHA-based materials, may negatively affect plant growth under certain conditions (Brodhagen et al., 2015; Zang et al., 2020; Qi et al., 2018, 2020).

Importantly, again here, these effects were not attributed to direct polymer toxicity, but rather to indirect soil-mediated mechanisms that arise during PHA biodegradation (Zhou et al., 2021). In the study by Nayab et al. (2022) PHB addition caused a 90–98% reduction in soil mineral nitrogen and soil available phosphorus, thus resulting in greater plant–microbe competition for nutrient resources, and leading to pronounced declines in plant growth and vigor. Brtnicky et al. (2022) attribute the reduced lettuce growth in their study to preferential microbial utilization of P3HB as a readily available carbon source, which intensified microbial activity and caused nitrogen immobilization in the soil. Undisclosed additives or contaminants in the polymer might also be responsible for induced or observed phytotoxicity (Zimmermann et al., 2020). Zhou et al. (2023) suggested that localized soil acidification associated with the release of 3-hydoxyacids’s during PHA depolymerization may exacerbate stress caused to plants under high polymer loadings. Although empirical evidence indicates that bulk soil pH and salinity changes remain minor. Indeed, Rychter et al. (2006) reported only slight increases in soil pH and salinity following PHA degradation, suggesting that acidification effects, if present, are likely transient and spatially restricted to degradation hotspots. Moreover, it is unlikely that an accumulation of the monomer will occur in soils, where, in fact, these monomers are rapidly metabolized by soil microorganisms (Mason-Jones et al., 2019). Mierziak et al. (2020) report a mechanistically distinct pathway for plant growth inhibition associated with PHB degradation, where accumulation of 3-HB alters plant gene regulation rather than soil nutrient dynamics. In their study, elevated 3-HB in transgenic flax or following exogenous application triggered chromatin remodeling and DNA demethylation, leading to activation of phenylpropanoid biosynthesis. This response reflects stress-induced metabolic reprogramming, suggesting that PHB degradation products can directly influence plant epigenetic and stress response pathways, with implications for attenuating the plant’s ability to resist the harmful impact of reactive oxygen species. Although empirical evidence indicates that bulk soil pH and salinity changes remain minor. Indeed, Rychter et al. (2006) reported only slight increases in soil pH and salinity following PHA degradation, suggesting that acidification effects, if present, are likely transient and spatially restricted to degradation hotspots. Furthermore, it is unlikely that an accumulation of the monomer will occur in soils, where, in fact, these monomers are rapidly metabolized by soil microorganisms (Mason-Jones et al., 2019). Mierziak et al. (2020) report a mechanistically distinct pathway for plant growth inhibition associated with PHB degradation, where accumulation of 3-HB alters plant gene regulation rather than soil nutrient dynamics. In their study, elevated 3-HB in transgenic flax or following exogenous application triggered chromatin remodeling and DNA demethylation, leading to activation of phenylpropanoid biosynthesis. This response reflects stress-induced metabolic reprogramming, suggesting that PHB degradation products can directly influence plant epigenetic and stress response pathways, with implications for attenuating the plant’s ability to resist the harmful impact of reactive oxygen species.

In any case, resultant plants grown under these conditions, therefore, often have been reported to exhibit classical nutrient stress responses, including chlorosis, reduced chlorophyll content, anthocyanin accumulation, and reduced tissue nitrogen concentrations (Brtnicky et al., 2022; Zhou et al., 2021). Brown et al. (2023) have previously reported changes in the shoot metabolome of *Zea mays* L., indicating indirect stress (i.e., nutrient and/or water deficiency), as a result of increasing PHBV-derived microplastics content in soil. Overall, the consensus is that at high, non-typical soil doses (≥1%), PHA-induced plant and biomass stress is a reproducible phenomenon, and they highlight an urgent need to elucidate the governing mechanisms and boundary conditions. Notably, measurements from agricultural soils receiving P(3HB) mulching films indicate that local polymer concentrations can already approach ~0.5–1.5% (w/w), placing them close to the threshold at which negative plant responses have been documented (Palucha et al., 2024). Therefore, as the adoption of PHA-based mulches expands and repeated applications accumulate residues over time, these findings underscore the need to carefully evaluate long-term loading scenarios, residue management, and nutrient balancing strategies to avoid unintended yield penalties. Brown et al. (2023) have previously reported changes in the shoot metabolome of *Zea mays* L., indicating indirect stress (i.e., nutrient and/or water deficiency), as a result of increasing PHBV-derived microplastics content in soil. Overall, the consensus is that at high, non-typical soil doses (≥1%), PHA-induced plant and biomass stress is a reproducible phenomenon, and they highlight an urgent need to elucidate the governing mechanisms and boundary conditions. Notably, measurements from agricultural soils receiving P(3HB) mulching films indicate that local polymer concentrations can already approach ~0.5–1.5% (w/w), placing them close to the threshold at which negative plant responses have been documented (Palucha et al., 2024). Therefore, as the adoption of PHA-based mulches expands and repeated applications accumulate residues over time, these findings underscore the need to carefully evaluate long-term loading scenarios, residue management, and nutrient balancing strategies to avoid unintended yield penalties.

Contrastingly, beyond neutrality or negative impact, there are other bunch of studies that indicate that PHA-based materials can support or enhance agronomic performance when properly formulated. For instance, Tian and Wang (2020) conducted field trials of PHA/PBAT mulch films in maize cultivation. The results showed improved soil temperature regulation compared to polyethylene mulches, along with enhanced seed germination, plant height, and stem diameter. Importantly, the films remained intact during the initial growth period and began to biodegrade only after ~60 days, which aligns with crop protection requirements. In a different application, PHA-conjugated herbicide delivery systems successfully suppressed broadleaf weeds without impairing the growth of faba bean *(Vicia faba),* illustrating the potential of PHAs as controlled release matrices for wide agricultural applications (Kwiecien et al., 2018). Other recently published work also demonstrates that nitrogen-rich organic amendments can partially offset PHA-induced nutrient stress in soils (Brtnicky et al., 2025a). In a pot experiment, Brtnicky et al. (2025a) showed that 1% (w/w) P3HB alone strongly suppressed lettuce growth, whereas co-application with digestate (C: N ~ 8.9) restored aboveground biomass and partially recovered root growth. In contrast, compost (C: N ~ 10.8) accelerated P3HB degradation but did not alleviate growth inhibition. This study is interesting as it clearly indicates that accelerated depolymerization alone is insufficient to prevent adverse biological effects. Instead, nutrient form and availability governed the plant responses. Overall, these studies highlight that PHA impacts on plants are highly dependent on formulation, timing, and doses, rather than polymer identity alone.

## 9 Conclusion

Across laboratory, mesocosm, and field-relevant studies, several conclusions regarding PHA behavior in agricultural soils are now well established. Neat PHAs and accessible PHA-rich domains are biodegradable through microbial extracellular depolymerases, and their low molecular weight breakdown products are generally readily assimilated under the biologically active soil conditions. However, this behavior should not be generalized to all PHA-containing blends or formulated materials, because non-PHA phases, additives, fillers, crystallinity, and phase architecture can substantially alter degradation rates and leave persistent residues. Importantly, reported negative effects on plant growth are not due to intrinsic polymer toxicity but arise from indirect soil-mediated processes, including microbial nutrient immobilization, altered C: N: P stoichiometry, and intensified competition between plants and soil microorganisms. PHA inputs act as a readily available carbon source, stimulating microbial respiration, enzyme activity, and shifts toward fast-growing degraders, while suppressing oligotrophic and nitrifying taxa. These responses are strongly dose-dependent: elevated concentrations (≥1% w/w) consistently induce nutrient limitation and plant growth inhibition, whereas field-realistic inputs generally show neutral effects on crop performance, microbial biomass, and greenhouse gas fluxes. However, as reflected in the SWOT analysis, the limitation is not only a small number of field studies, but also the restricted scope of measurements within those studies. Existing field evaluations of PHA-based agricultural materials have primarily focused on mulch performance, visible degradation, mass loss, crop response, and selected bulk soil properties. However, these studies rarely integrate degradation kinetics with intermediate fragment fate, microbial functional responses, nutrient cycling, greenhouse-gas fluxes, and crop outcomes across repeated seasons. Thus, the current field evidence remains fragmented: it can describe whether PHA-based materials perform agronomically or visibly degrade, but it does not yet resolve how repeated PHA inputs influence coupled soil–microbe–plant processes over time. This gap is reflected in the revised SWOT weaknesses, which now specify a few >18-month field studies and no integrated soil-crop datasets. Overall, PHA degradation in soils is not governed by polymer chemistry alone but emerges from interactions among material properties, soil conditions, and microbial capacity. Accordingly, PHAs should be regarded as conditionally compatible materials, whose environmental performance depends on degradation context, formulation design, and agronomic management.

### 9.1 Research gaps and future directions

Despite these advances, several critical uncertainties limit the reliable deployment of PHAs in agricultural soils. First, the timescale and completeness of degradation under field conditions remain poorly constrained, as most studies are short-term and often show partial degradation that reflects biological and environmental limitations rather than material persistence. Second, current biodegradation standards rely on outcome-based metrics such as CO₂ evolution or mass loss but do not account for microbial capacity or depolymerase availability, leading to inconsistent and potentially misleading interpretations across soils. Third, the formation and fate of intermediate degradation products, including micro- and nano-scale fragments, remain insufficiently characterized, leaving key questions regarding physical degradation pathways unresolved. Fourth, degradation is inherently spatially heterogeneous, yet most studies assume uniform behavior, with limited understanding of rhizosphere-specific processes where plant–microbe interactions directly control nutrient dynamics and plant responses. Fifth, the impact of PHA degradation on greenhouse gas fluxes, particularly methane dynamics under field conditions, remains largely unexplored. Finally, formulation-dependent effects introduce substantial uncertainty, as additives, blends, and processing conditions can significantly alter degradation kinetics and biological responses but are often poorly reported.

Moreover, the formation and environmental implications of intermediate degradation products during PHA breakdown remain insufficiently resolved. PHA degradation in soil typically proceeds through a microbially mediated surface-erosion process, where extracellular depolymerases generate soluble low-molecular-weight products, including monomers (3-hydroxybutyrate, 3-hydroxyvalerate) and short oligomers. Under biologically active conditions in soil, these intermediates are generally rapidly assimilated or mineralized (Gebauer and Jendrossek, 2006). However, degradation rates cannot be generalized across all soil environments because degradation rates are strongly constrained by moisture, nutrient availability, microbial activity, and polymer loading. For example, complete degradation of PHB/PHBV films has been observed within 14 days under water-saturated conditions, but shows minimal change after 43 days under drier conditions (Kim et al., 2023). Similarly, low degradation rates (1.5–5%) over 8 weeks have been reported for PHBV microplastics under nutrient-limited conditions (Reay et al., 2025). Accordingly, these observations suggest that while long-term accumulation of intermediates is unlikely in highly biologically active soils, transient retention of soluble oligomers or particulate fragments under environmentally constrained conditions cannot be excluded. This issue is particularly relevant for PHA-based mulch films because physical fragmentation may occur before complete mineralization, creating the possibility of micro-or nano-sized residues during intermediate stages of breakdown. At present, however, PHA studies rarely track particle-size evolution during degradation. Therefore, whether PHA-based films generate environmentally relevant quantities of nanoparticles, how rapidly such particles mineralize, and whether they produce stronger biological effects than larger fragments remain unresolved. Critically, most studies do not directly quantify intermediate products, relying instead on bulk mass loss or CO_2_ evolution, leaving their formation, transformation, and persistence poorly constrained. Future studies should therefore pair mineralization measurements with particle size tracking, residual polyester recovery, oligomer analysis, nanoparticle detection, and ecotoxicological testing of micro- and nano-sized degradation products.

**Figure 6:**
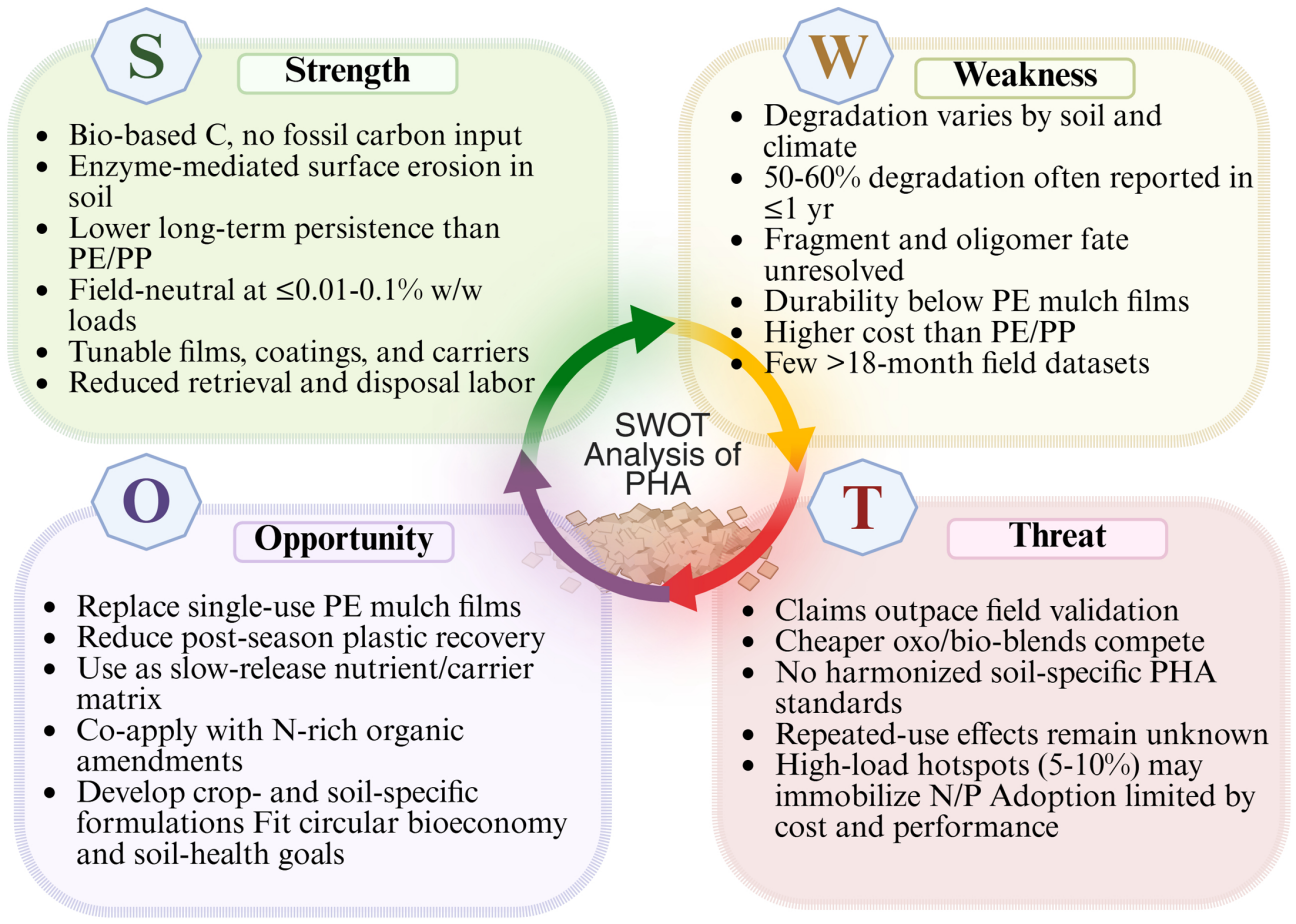
Key strengths, weaknesses, opportunities, and threats (SWOT) associated with the use of PHA in agricultural soils, highlighting material performance, environmental interactions, regulatory context, and adoption challenges.

Taken together, these unresolved issues indicate that PHAs should not yet be regarded as universally benign materials in agricultural soils. Rather, they should be viewed as conditionally compatible, with outcomes that depend strongly on degradation context, formulation design, soil biology, and management practices, as outlined in Figure 6. Addressing these gaps will require long-term, field-based, and mechanistically resolved studies that explicitly link degradation pathways, microbial dynamics, nutrient cycling, and plant responses. Such integrated approaches are essential to move beyond simplified laboratory interpretations and enable confident, context-specific deployment of PHAs in agricultural systems.

## Data Availability

The original contributions presented in the study are included in the article/supplementary material, further inquiries can be directed to the corresponding author.
